# Supplementary material for: Functional diversity among cardiolipin binding sites on the mitochondrial ADP/ATP carrier
Source: EMBO J. 2024 Jun 5;43(14):2979–3008. doi: 10.1038/s44318-024-00132-2 (PMC11251061; doi:10.1038/s44318-024-00132-2)
Supplement: Supplementary file 1 — Appendix [file 44318_2024_132_MOESM1_ESM.pdf]

# Appendix

## Functional diversity among cardiolipin binding sites on the mitochondrial ADP/ATP carrier

Nanami Senoo, Dinesh K. Chinthapalli, Matthew G. Baile, Vinaya K. Golla, Bodhisattwa Saha, Abraham O. Oluwole, Oluwaseun B. Ogunbona, James A. Saba, Teona Munteanu, Yllka Valdez, Kevin Whited, Macie S. Sheridan, Dror Chorev, Nathan N. Alder, Eric R. May, Carol V. Robinson, Steven M. Claypool

**Table of Contents:**

Appendix figures S1-16 Pages 2-17

Appendix tables S1-4 Pages 18-23

References Page 24

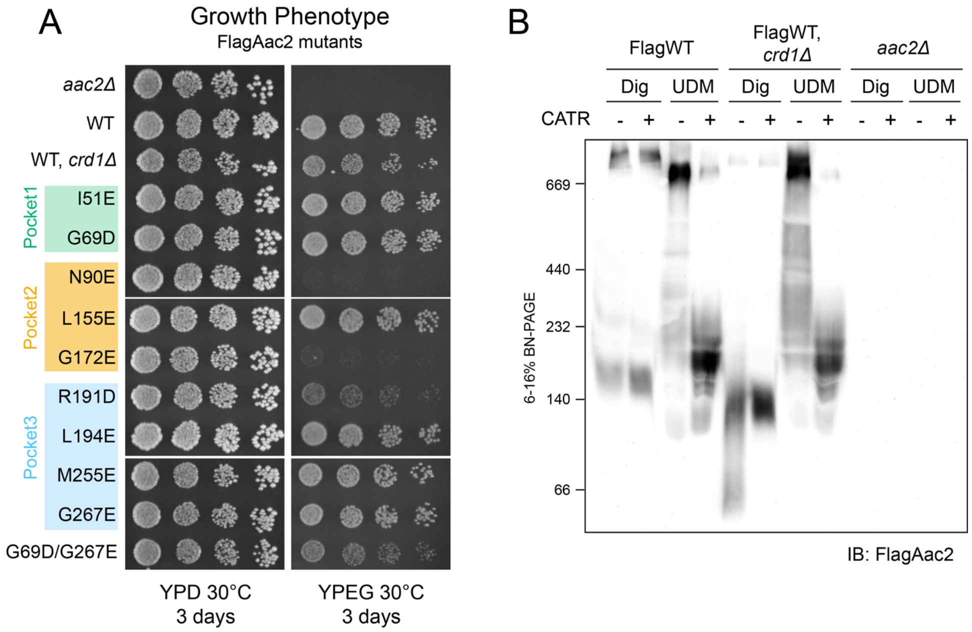


**Appendix fig. S1: Characterization of Flag-tagged WT and mutant Aac2**. (A) Growth phenotype of Flag-tagged Aac2 CL-binding mutants. Serial dilutions of indicated cells were spotted onto fermentable (YPD) and respiratory (YPEG) media and incubated at 30°C for 3 days (n=3, biological replicates). (B) Mitochondria from indicated strains were mock- or pre-treated with 40 μM CATR. The treated mitochondria were then solubilized with 1.5% (w/v) digitonin or 2% (w/v) UDM, resolved by 6 to 16% blue native-PAGE and immunoblotted for Flag. Representative image from the replicates (n=3, biological replicates) is shown.

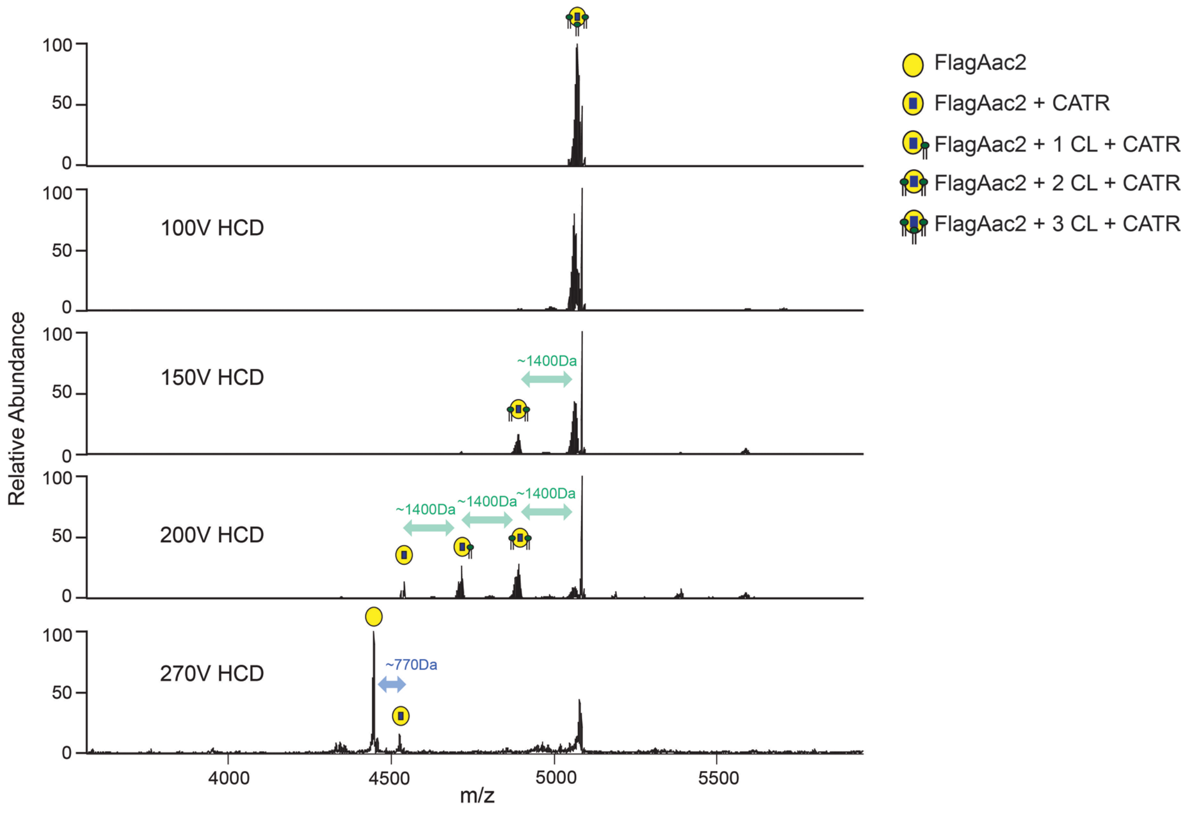


**Appendix fig. S2: Three CL molecules associated with Aac2.** Related to Fig. 2, MSMS performed against Aac2 + 3CL + CATR complex (*m/z* 5069 Da). Increased high collision dissociation (HCD) yielded spectra corresponding to CL (~1400 Da) and CATR (~770 Da).

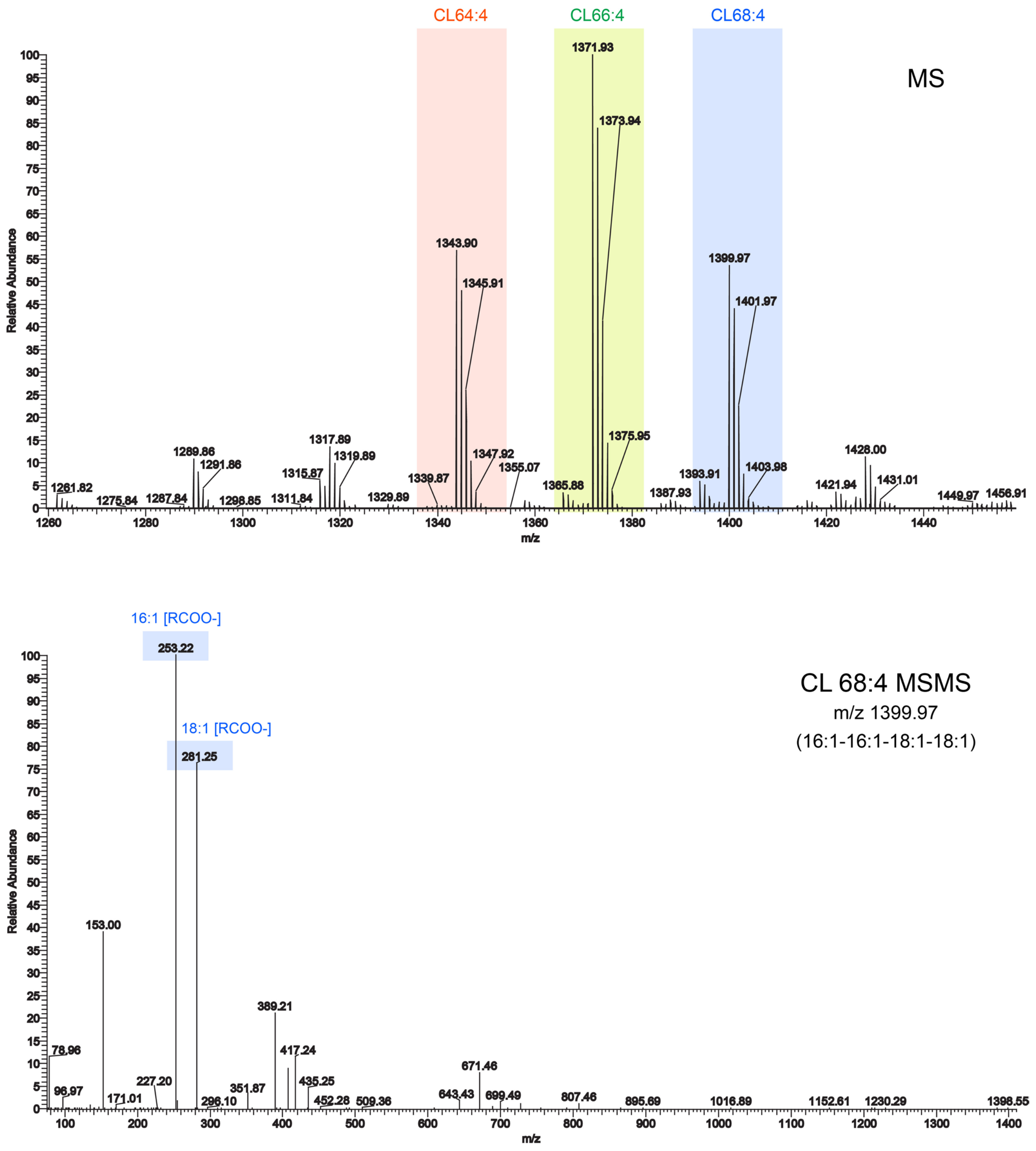


**Appendix fig. S3: CL species interacting with yeast Aac2.** Mass spectrometry (MS) analysis detected three types of CL species that co-purified with FlagAac2 from WT mitochondria (Top). MSMS performed against CL 68:4 yielded unique fragments corresponding to acyl-chains derived from CL (Bottom).

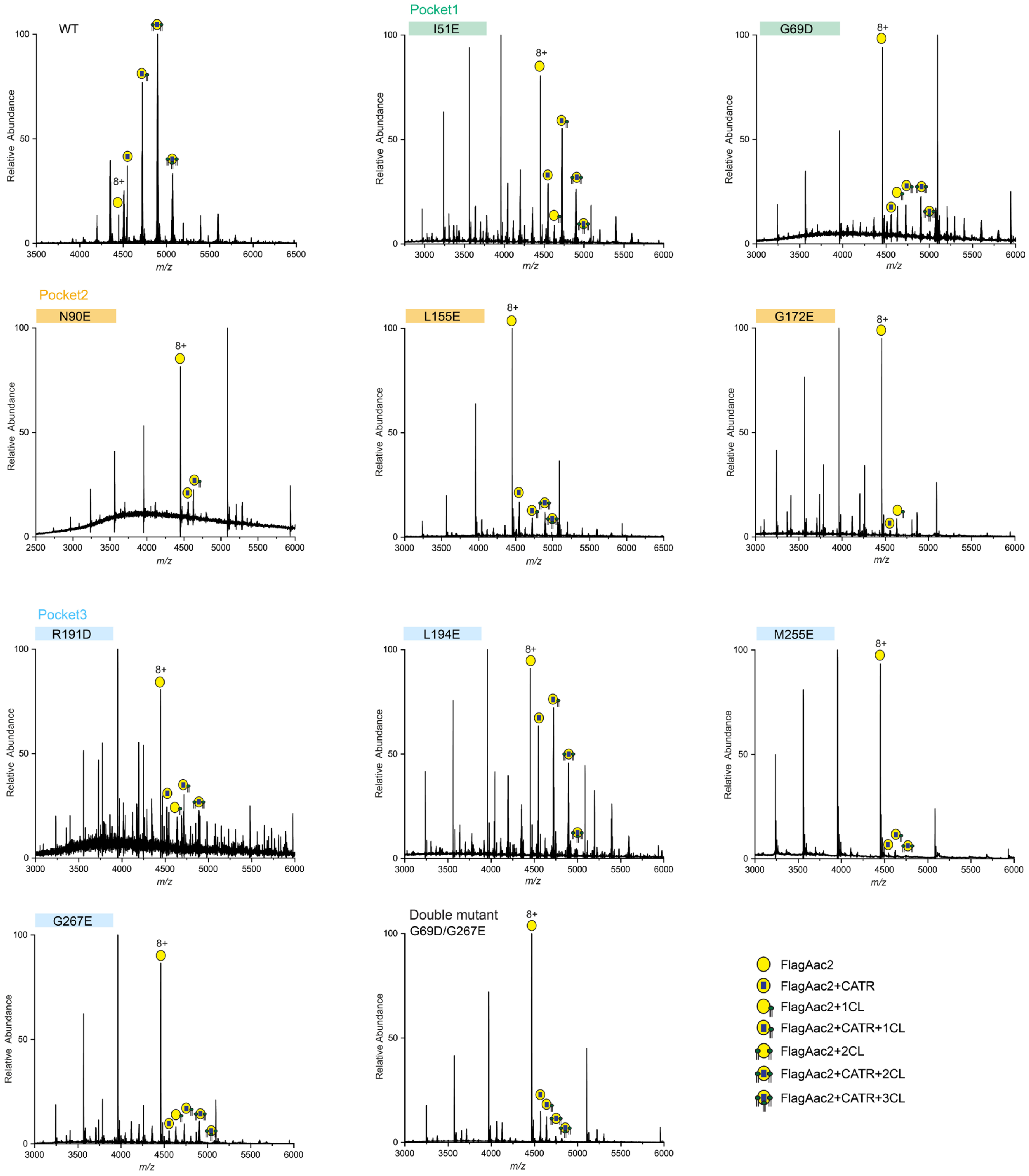


**Appendix fig. S4: MS spectra for the distribution of CL interactions of Aac2 mutants.** Related to Fig. 2, representative MS spectra of indicated Aac2 mutants are shown.

A

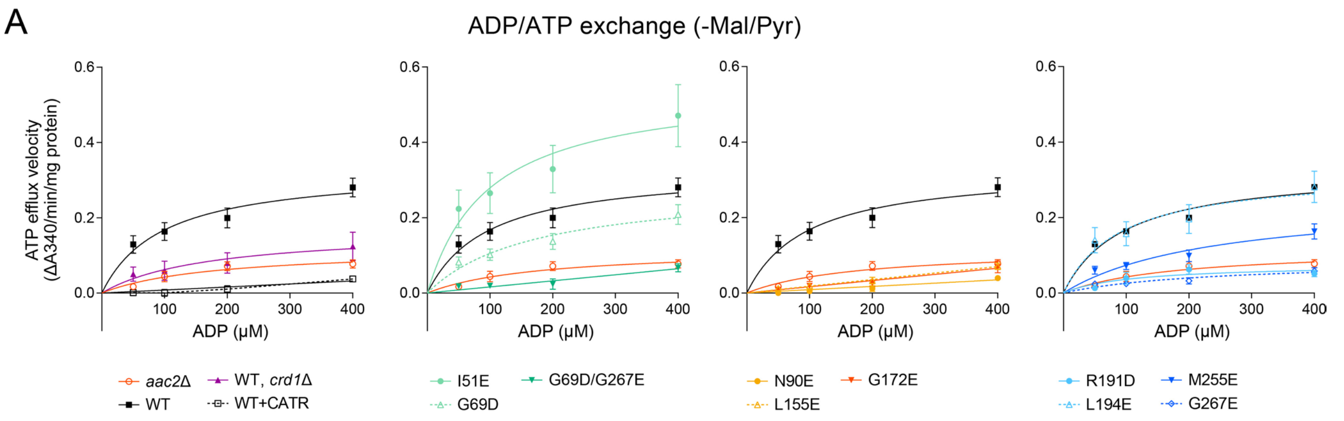


B

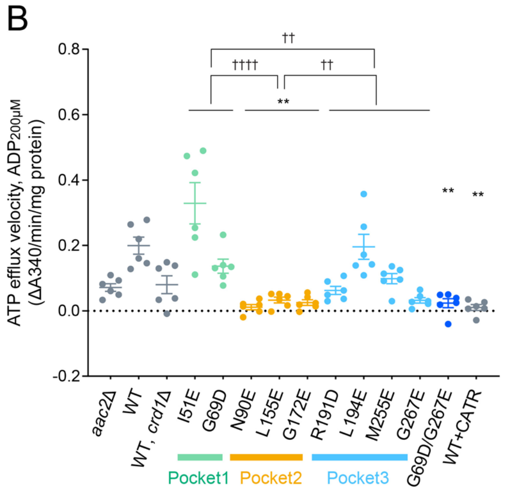


C

| | -Mal/Pyr | | | |
|---|---|---|---|---|
| | Vmax [95% CI] (μM/min/mg protein) | | Km [95%CI] (μM) | |
| *aac2Δ* | 0.1189 | [0.07293–0.3012] | 180.0 | [50.25–894.0] |
| WT | 0.3298 | [0.2442–0.4902] | 96.61 | [34.10–252.2] |
| WT, *crd1Δ* | 0.1682 | [n.d.] | 170.4 | [n.d.] |
| I51E | 0.5506 | [0.3491–1.163] | 97.38 | [14.52–485.5] |
| G69D | 0.2848 | [0.1881–0.5864] | 171.1 | [54.89–640.9] |
| N90E | n.d. | [n.d.] | n.d. | [n.d.] |
| L155E | 2.548 | [n.d.] | 14171 | [n.d.] |
| G172E | n.d. | [n.d.] | n.d. | [n.d.] |
| R191D | 0.07614 | [0.04341–0.1947] | 111.0 | [14.06–707.4] |
| L194E | 0.3212 | [0.2071–0.6405] | 89.75 | [12.82–421.9] |
| M255E | 0.2499 | [0.1548–0.7122] | 240.0 | [75.71–1231] |
| G267E | 0.08628 | [n.d.] | 234.7 | [n.d.] |
| G69D/G267E | n.d. | [n.d.] | n.d. | [n.d.] |
| WT +CATR | n.d. | [n.d.] | n.d. | [n.d.] |

**Appendix fig. S5: ADP/ATP exchange of Aac2 CL-binding mutants without respiratory substrates.** The efflux of matrix ATP was detected with isolated mitochondria as in Fig. 4A-C. The measurement was performed in the absence of malate and pyruvate (-Mal/Pyr). WT + CATR: WT mitochondria were treated with 5 μM CATR prior to the efflux reaction (n=6, biological replicates). (A) The linear part of the initial velocity for the ATP efflux was plotted and curve fitting performed by nonlinear regression (mean with SEM). Plots of *aac2*Δ and WT are repeated in all panels. (B) The initial linear velocity following the addition of 200 μM ADP shown as scatter plots (mean with SEM). (C) Fitted Km and Vmax values were obtained using the Michaelis-Menten equation (mean).

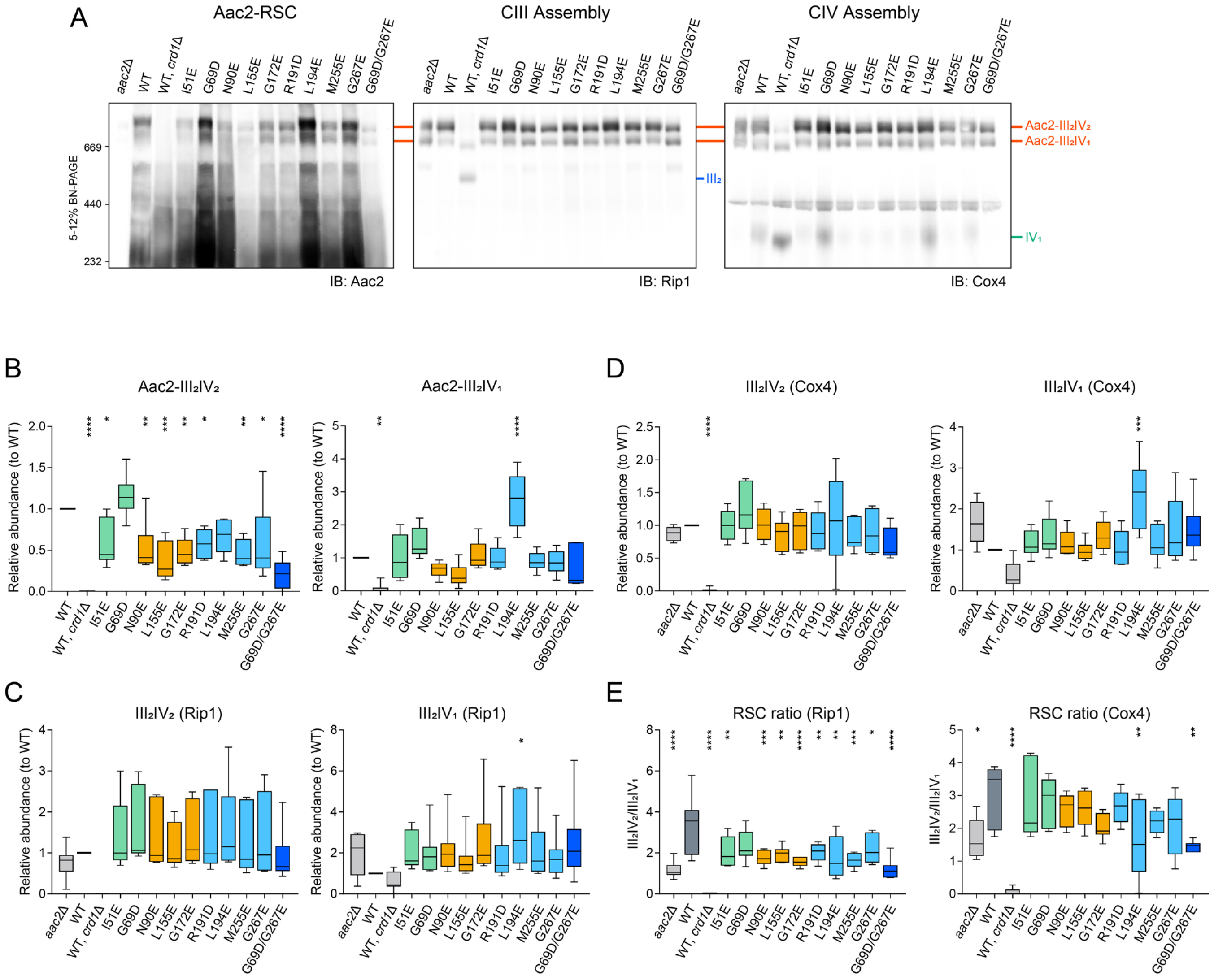


**Appendix fig. S6: Assembly of Aac2 CL-binding mutants and respiratory supercomplexes is modestly altered.** (A) WT and mutant mitochondria were solubilized with 1.5% (w/v) digitonin, resolved by 5 to 12% blue native-PAGE, and immunoblotted (IB) as indicated. RSC, respiratory supercomplex. (B-D) Quantification of assembled Aac2 (B), Rip1 (C), and Cox4 (D) within respiratory supercomplexes. (E) Ratios of respiratory supercomplexes $III_2IV_2$ and $III_2IV_1$ when detected by Rip1 and Cox4, respectively. Data are shown as box-whisker plots with the box extended from 25th to 75th percentiles and the whiskers indicating the min to max range. One-way ANOVA followed by Dunnett's multiple comparison test determined the significance; *p<0.05, **p<0.01, ***p<0.001, ****p<0.0001. Representative images from the replicates (n=5-6, biological replicates) are shown; images have been cropped to exclude the abundant Aac2 monomer to facilitate visualization of the Aac2-RSC complexes.

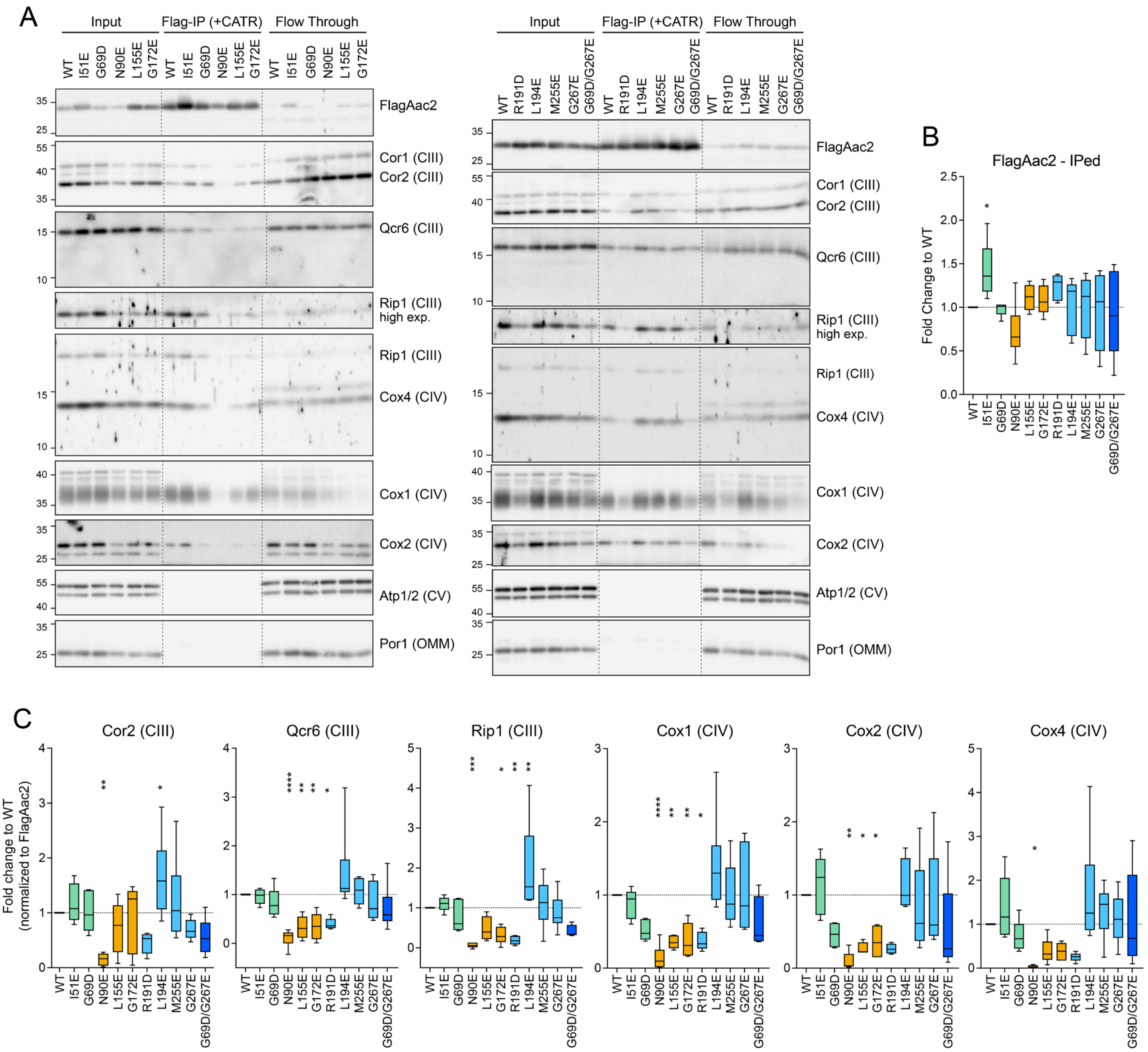


**Appendix fig. S7: Protein-protein interaction between Aac2 and respiratory complex subunits are diminished in Aac2 CL-binding mutants.** (A) Isolated mitochondria from Flag-tagged WT and mutant Aac2 strains were pre-incubated with 40 µM CATR and then solubilized with 1.5% (w/v) digitonin. The mitochondrial extracts were immunoprecipitated (IP) using anti-Flag resin. Co-purified subunits of complexes III and IV were determined by immunoblotting; Atp1/2 and Por1 served as controls. Four percent of input (intact mitochondria) and flow through (unbound) was analyzed. (B) The abundance of FlagAac2 eluted upon IP. (C) The abundance of subunits of complexes III and IV co-purified with FlagAac2 was quantified and normalized. Data are shown as box-whisker plots with the box extended from 25th to 75th percentiles and the whiskers indicating the min to max range. Statistical differences were analyzed by one-way ANOVA followed by Dunnett's multiple comparison test; *$p<0.05$, **$p<0.01$, ***$p<0.001$, ****$p<0.0001$ (vs. WT). Representative images from the replicates (n=4-12, biological replicates) are shown.

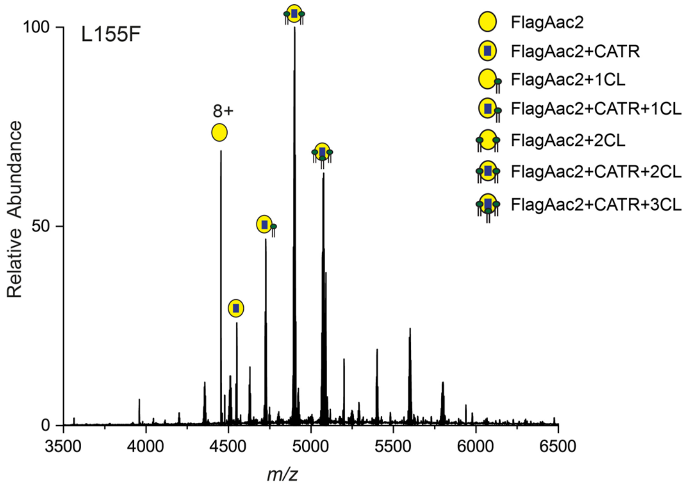


**Appendix fig. S8: MS spectrum for the distribution of CL interactions of Aac2 L155F mutant.** Related to Fig. 5B, a representative MS spectrum is shown.

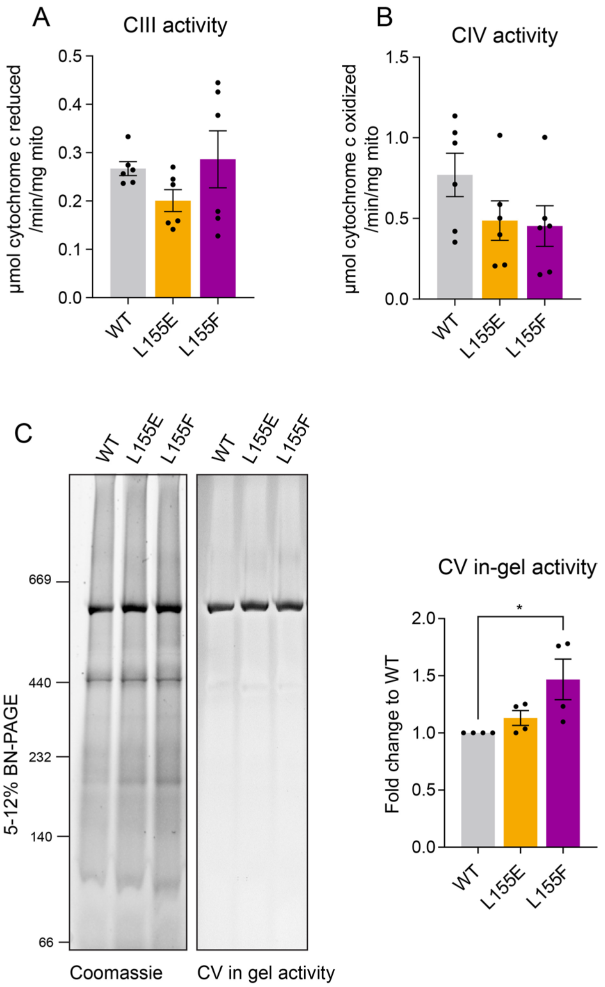


**Appendix fig. S9: Activities of respiratory complexes III, IV, and V of yeast Aac2 L155 mutants.** (A) Complex III activity in 0.5% (w/v) DDM-solubilized mitochondria (n=6, biological replicates). (B) Complex IV activity in 0.5% (w/v) DDM-solubilized mitochondria (n=6, biological replicates). (C) Complex V in-gel activity assay. Mitochondria were solubilized in 1% (w/v) DDM, resolved by 5-12% blue native-PAGE and incubated with the substrate (n=4, biological replicates). Mean with SEM. Statistical differences were analyzed by one-way ANOVA.

A

```
                                                  R106H
P12235|ANT1 ------------MGDHAWSFLKDFLAGGVAAAVSKTAVAPIERVKLLLQVQHASKQISAEKQYKGIIDCVVRIPKEQGFLSFWRGNLANVIRYFPTQALNF 89
P05141|ANT2 ------------MTDAAVSFAKDFLAGGVAAAISKTAVAPIERVKLLLQVQHASKQITADKQYKGIIDCVVRIPKEQGVLSFWRGNLANVIRYFPTQALNF 89
P12236|ANT3 ------------MTEQAISFAKDFLAGGIAAAISKTAVAPIERVKLLLQVQHASKQIAADKQYKGIVDCIVRIPKEQGVLSFWRGNLANVIRYFPTQALNF 89
Q9H0C2|ANT4 MHREPAKKKAEKRLFDASSFGKDLLAGGVAAAVSKTAVAPIERVKLLLQVQASSKQISPEARYKGMVDCLVRIPREQGFFSFWRGNLANVIRYFPTQALNF 101
                                * ** **:****:***:***************** :****: : :***::**:****:***.:*********************

                            R106H                                  148-AGA/SGT-150    Y165T K171R
P12235|ANT1 AFKDKYKQLFLGGVDRHKQFWRYFAGNLASGGAAGATSLCFVYPLDFARTRLAADVGKGAAQREFHGLGDCIIKIFKSDGLRGLYQGFNVSVQGIIIYRAA 190
P05141|ANT2 AFKDKYKQIFLGGVDKRTQFWLYFAGNLASGGAAGATSLCFVYPLDFARTRLAADVGKAGAEREFRGLGDCLVKIYKSDGIKGLYQGFNVSVQGIIIYRAA 190
P12236|ANT3 AFKDKYKQIFLGGVDKHTQFWRYFAGNLASGGAAGATSLCFVYPLDFARTRLAADVGKSGTEREFRGLGDCLVKITKSDGIRGLYQGFSVSVQGIIIYRAA 190
Q9H0C2|ANT4 AFKDKYKQLFMSGVNKEKQFWRWFLANLASGGAAGATSLCVVYPLDFARTRLGVDIGKGPEERQFKGLGDCIMKIAKSDGIAGLYQGFGVSVQGIIVYRAS 202
            ********:*:.**::..*** :* .**************.***********..*:**.  :*:*:*****::** ****: ******.*******:***:

                                       T227V            T247A          A262F
P12235|ANT1 YFGVYDTAKGMLPDPKNVHIFVSWMIAQSVTAVAGLVSYPFDTVRRRMMMQSGRKGADIMYTGTVDCWRKIAKDEGAKAFFKGAWSNVLRGMGGAFVLVLY 291
P05141|ANT2 YFGIYDTAKGMLPDPKNTHIVISWMIAQTVTAVAGLTSYPFDTVRRRMMMQSGRKGTDIMYTGTLDCWRKIARDEGGKAFFKGAWSNVLRGMGGAFVLVLY 291
P12236|ANT3 YFGVYDTAKGMLPDPKNTHIVVSWMIAQTVTAVAGVVSYPFDTVRRRMMMQSGRKGADIMYTGTVDCWRKIFRDEGGKAFFKGAWSNVLRGMGGAFVLVLY 291
Q9H0C2|ANT4 YFGAYDTVKGLLPKPKKTPFLVSFFIAQVVTTCSGILSYPFDTVRRRMMMQSGEA--KRQYKGTLDCFVKIYQHEGISSFFRGAFSNVLRGTGGALVLVLY 301
            *** ***.**:**.**:. :.:*::*** **: :*: ****************.   .  *.**:**: ** :.** .:**:**:****** ***:*****

P12235|ANT1 DEIKKYV------- 298
P05141|ANT2 DEIKKYT------- 298
P12236|ANT3 DELKKVI------- 298
Q9H0C2|ANT4 DKIKEFFHIDIGGR 315
            *::*:
```

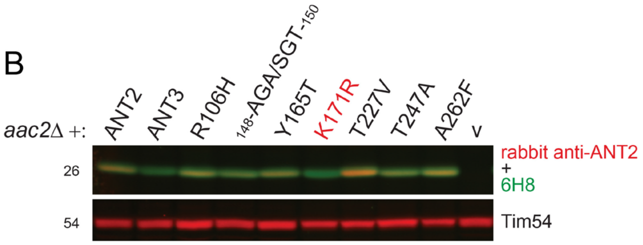


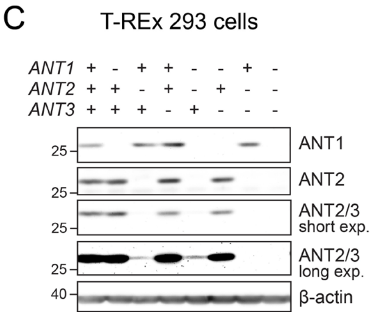


**Appendix fig. S10: Endogenous expression of three ANT isoforms was absent in ant^null cells.** (A, B) Epitope mapping of ANT2 antisera. (C) The expression of three ANT isoforms was detected in whole cell extracts by immunoblot (n=5, biological replicates). The absence of ANT1, ANT2, and ANT3 was confirmed in ant^null cells (right-most lane). Representative images from the indicated replicates in B and C are shown.

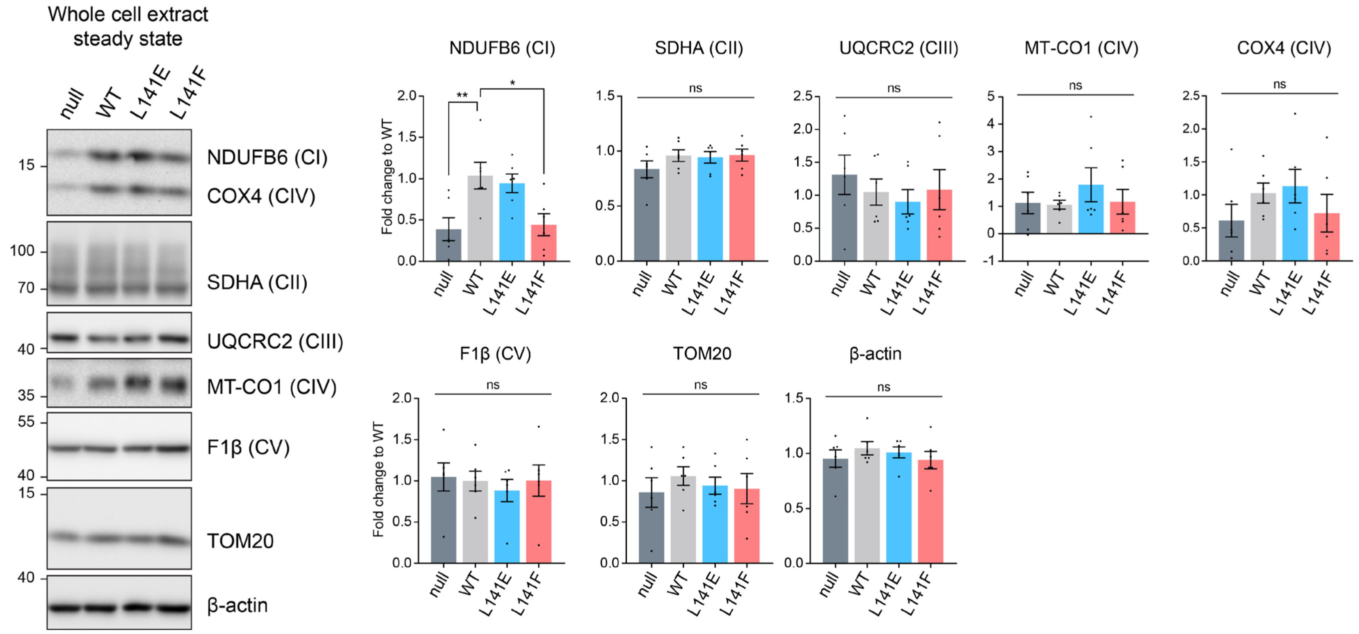


**Appendix fig. S11: Abundance of OXPHOS complex subunits in human ANT1 L141 mutants**. Whole cell extracts from wild type and the indicated ANT1 mutant lines were analyzed by immunoblotting against subunits of respiratory chains and complex V. TOM20 and β-actin served loading controls (n=6, biological replicates). Mean with SEM. Significant differences were obtained by one-way ANOVA with Dunnett's multiple comparisons test (vs. WT); *p<0.05, **p<0.01, ***p<0.001, ****p<0.0001.

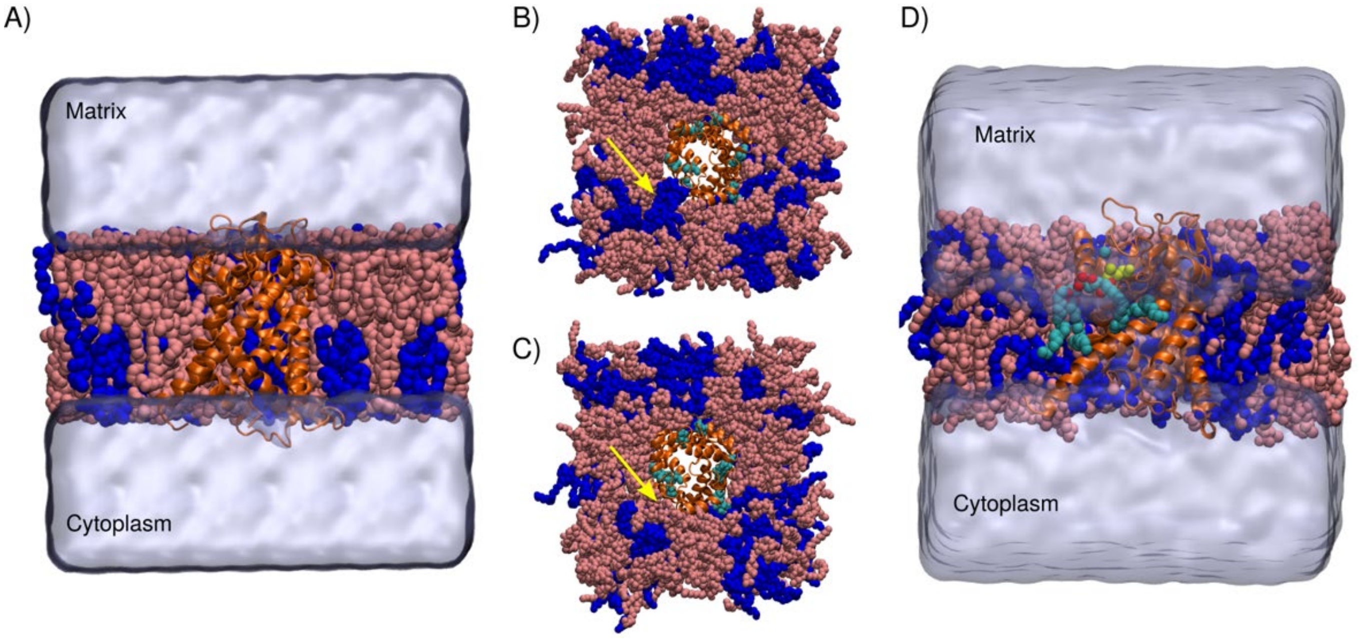


**Appendix fig. S12: Human ANT1 simulation system setup (c-state).** (A) The ANT1 protein (Orange cartoon), POPC (pink), and TLCL2 (tetralinoleoyl-cardiolipin $(18:2)_4$ in di-anionic form) (blue) were solvated in water (iso-blue surface). The top view (matrix view) of the ANT1 system setup for the equilibrium prebound (B) and equilibrium unbound (C) simulations. Yellow arrows point to the presence or absence of CL lipid around pocket 2. (D) Human ANT1 free energy perturbation (FEP) calculation system setup showing the "ligand CL", LIG (head group oxygen atoms in red, phosphorous atoms in green and acyl chain atoms in cyan van der Waals representation). The front portion of the membrane, hydrogen atoms, and the water molecules were removed for clarity.

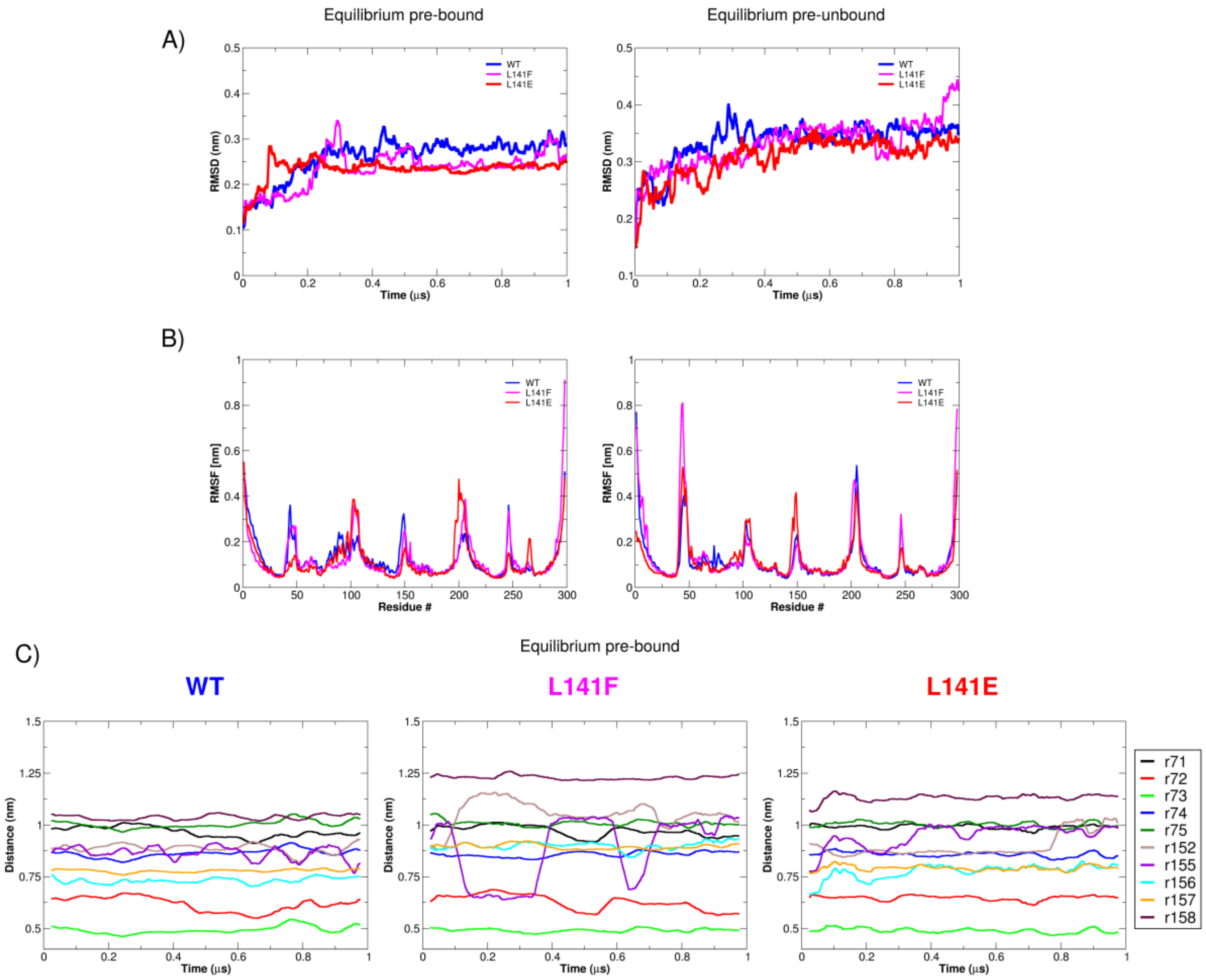


**Appendix fig. S13: ANT1 protein dynamics during MD simulations.** (A) Root-mean-squared deviation (RMSD) in prebound (left) and unbound (right) 1 µs simulations; 100 frame running averaging was performed to smooth the curves. (B) Root-mean-squared fluctuations (RMSF) for prebound (left) and unbound (right) simulations. (C) Calculated distances between the Cα atoms of residue 141 with that of the selected neighboring and pocket 2 binding site residues (residues 71, 72, 73, 74, 75, 152, 155, 156, 157, and 158) during prebound simulations.

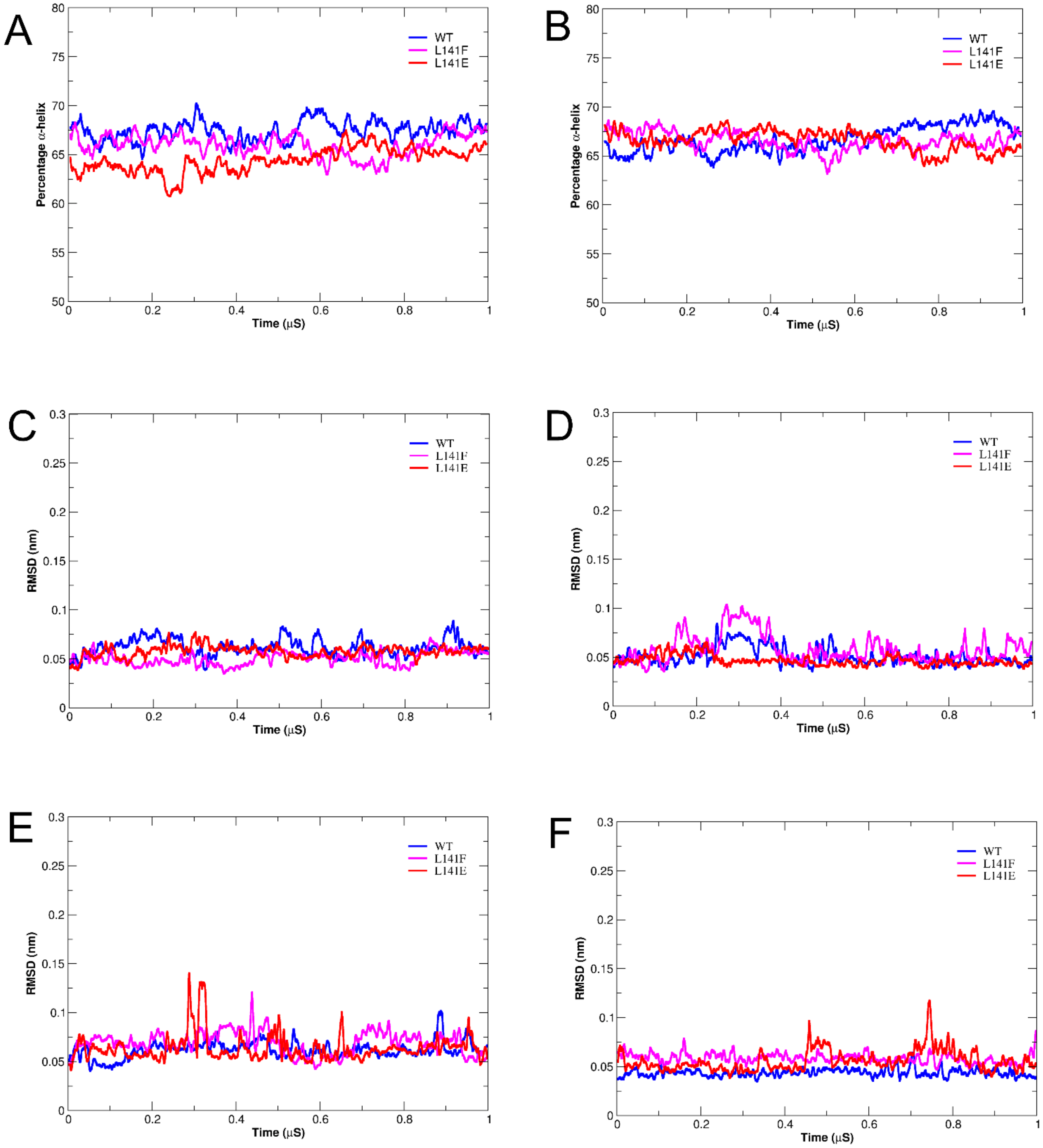


**Appendix fig S14: Global and Local Stability Analysis during MD Simulations.** The total $\alpha$-helical content in ANT1 during prebound (A) and unbound (B) simulations. Local RMSD analysis during prebound simulations around pocket 1 (C) and pocket 3 (D). Local RMSD analysis during unbound simulations around pocket 1 (E) and pocket 3 (F). **Note:** Local RMSD was estimated using the C-alpha residues of **Pocket 1:** 36, 53, 54, 55, 271, 272, 273, 274, 275, and 276; and **Pocket 3:** 251, 252, 253, 254, 255, 174, 175, 176, 177, and 178, respectively. $\alpha$-helical data is displayed using 10 ns running average smoothing.

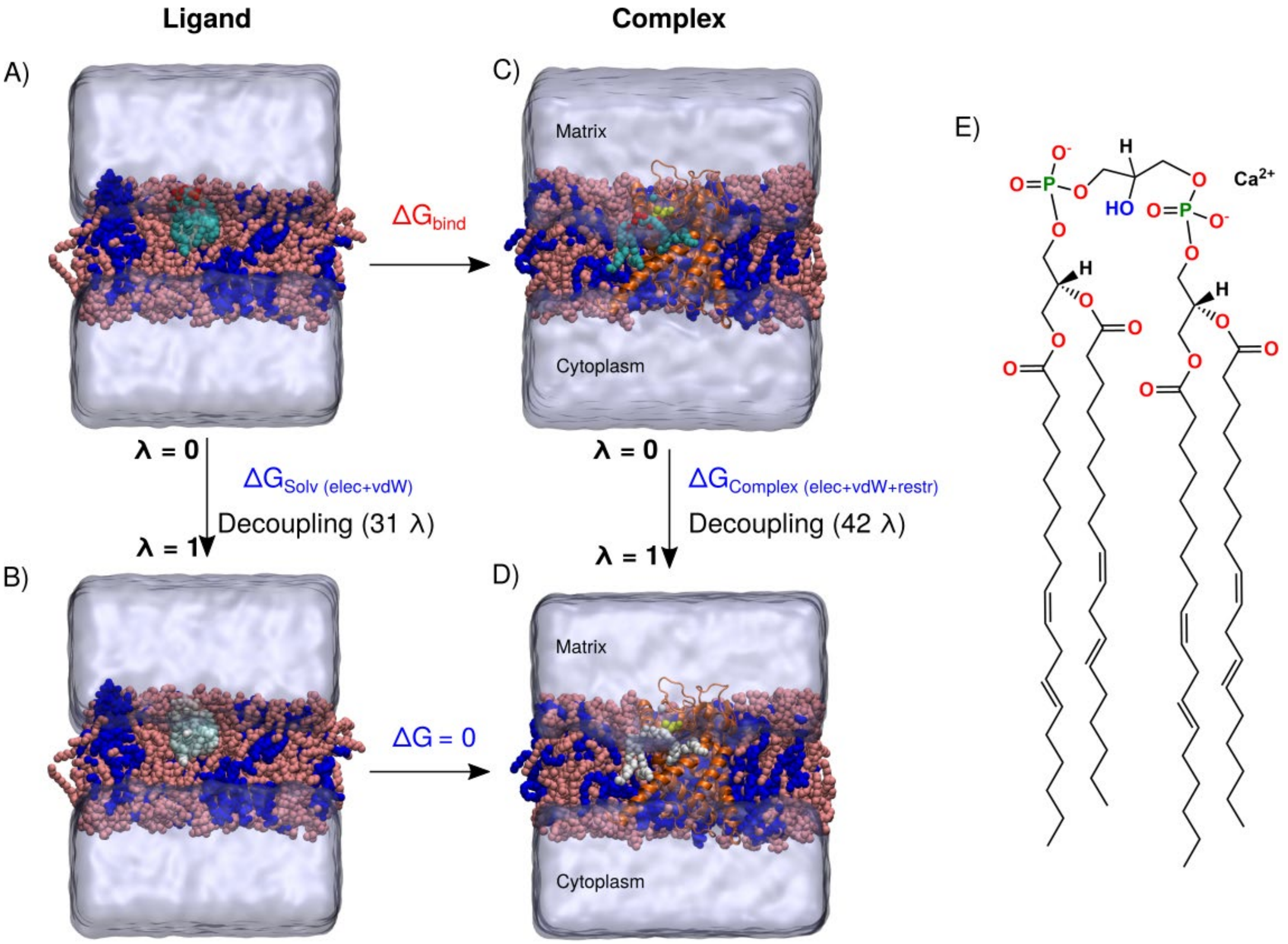


**Appendix fig. S15: FEP thermodynamic cycle.** (A) The fully integrated CL LIG in a bilayer environment is transformed into a completely non-interacting ligand (B, white) during a series of 31 equilibrium simulations in which corresponding electrostatic and van der Waals interactions are scaled to zero. The fully interacting LIG at the top right (C) is transformed into a completely non-interacting ligand (D, white) in the presence of ANT1 membrane protein during a series of 42 equilibrium simulations in which corresponding restraints, electrostatic, and van der Waals interactions are scaled to zero. The ANT1 protein (Orange cartoon), the POPC and TLCL2 membrane lipids (pink and blue van der Waals representation), and ligand LIG (head group oxygen atoms in red, phosphorous atoms in green and acyl chain atoms in cyan van der Waals representation) were solvated in water (iso-blue surface). (E) 2D structure of LIG used in the present study FEP calculations including the $Ca^{+2}$ which was simultaneously decoupled with LIG to maintain charge neutrality.

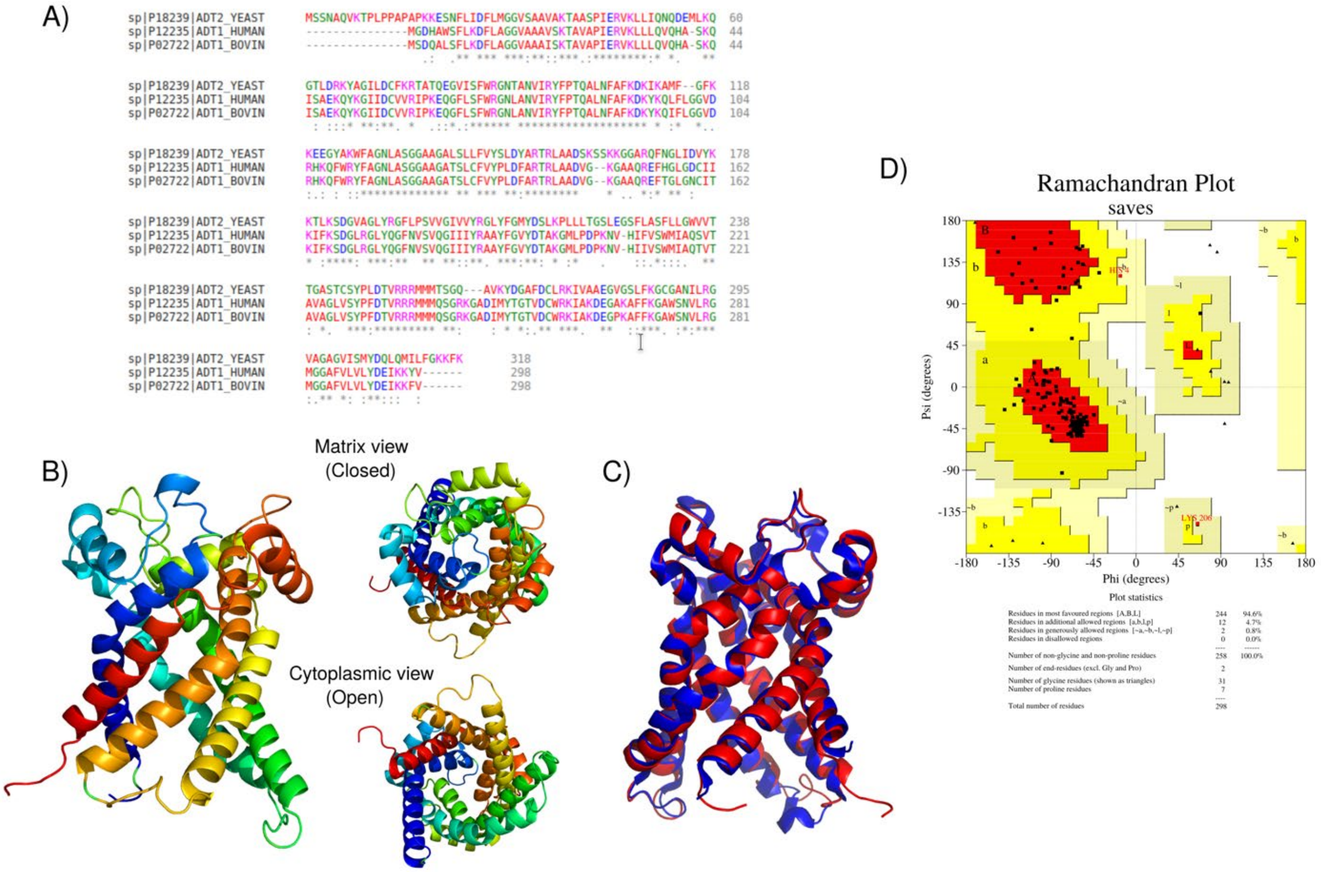


**Appendix fig. S16: Human ANT1 homology modeling.** (A) Sequence alignment of Human ANT1 with Bovine ANT1 and Yeast AAC2. Bovine ANT1 (PDB ID: 1OKC and 2C3E) was used as a template. (B) Cartoon representation of the generated Human ANT1 homology model (97% Quality factor). (C) Overlap structures of Bovine ANT1 (1OKC) and Human ANT1 model proteins. (D) Ramachandran plot of ANT1 model from Human generated by PROCHECK: in which 94.6 % residues in favorable regions; 4.7 % residues in additional allowed regions; 0.8 % residues in generously allowed regions; 0% residues in disallowed regions.

**Appendix table S1: Primers used to generate yeast mutant constructs.**

| Target | Type | Sequence (5'-3') |
|---|---|---|
| Aac2 5' UTR (SalI) | Forward | ACGCGTCGACGAGCACTGTTTCCAATGGAG |
| Aac2 3' End (NotI) | Reverse | GTGGCGGCCGCTCTTATTTGAACTTCTTACCAAAC |
| Aac2 I51E | Forward | ACTTTTGGAACAAAACCAAGATGAAATGTTAAAAC |
| Aac2 I51E | Reverse | ATCTTGGTTTTGTTCCAAAAGTTTAACTCTTTCGATGG |
| Aac2 G69D | Forward | AAAATACGCAGATATCTTAGACTGTTTCAAGAGAAC |
| Aac2 G69D | Reverse | CAGTCTAAGATATCTGCGTATTTTCTGTCCAAAG |
| Aac2 N90E | Forward | GGAGAGGTGAGACTGCTAACGTTATCCGTTATTTC |
| Aac2 N90E | Reverse | GTTAGCAGTCTCACCTCTCCAGAATGAGATAAC |
| Aac2 L155E | Forward | CAAGAACTAGAGAAGCTGCTGACTCCAAGTC |
| Aac2 L155E | Reverse | AGCAGCTTCTCTAGTTCTTGCATAATCCAAAG |
| Aac2 G172E | Forward | GTCAATTCAACGAATTGATCGATGTCTACAAGAAG |
| Aac2 G172E | Reverse | CGATCAATTCGTTGAATTGACGAGCACC |
| Aac2 R191D | Forward | GGTCTTTACGACGGTTTCTTACCTTCTGTCGTTG |
| Aac2 R191D | Reverse | AAGAAACCGTCGTAAAGACCAGCAACACCATC |
| Aac2 L194E | Forward | CAGAGGTTTCGAACCTTCTGTCGTTGGTATTG |
| Aac2 L194E | Reverse | CAGAAGGTTCGAAACCTCTGTAAAGACCAG |
| Aac2 M255E | Forward | AAGAAGAGAGATGATGACCTCCGGTCAAGC |
| Aac2 M255E | Reverse | GAGGTCATCATCTCTCTTCTTCTAACGGTATCCAATG |
| Aac2 G267E | Forward | GTTAAGTACGACGAAGCCTTTGACTG |
| Aac2 G267E | Reverse | AAAGGCTTCGTCGTACTTAACAGC |
| Aac2 L155F | Forward | AAGAACTAGATTCGCTGCTGACTCCAAGTCCTC |
| Aac2 L155F | Reverse | GAGTCAGCAGCGAATCTAGTTCTTGCATAATCC |
| Aac2 N-term Flag | Forward | ATGGATTATAAAGATGATGACGATAAAATGTCTTCCAACGCCCAAGTC |
| Aac2 N-term Flag | Reverse | TTTATCGTCATCATCTTTATAATCCATGGCTATTTGCTTATATGTATGTTAATGT |
| Aac2 K112D | Forward | AAGGACAAGATCGATGCCATGTTTGGTTTCAAG |
| Aac2 K112D | Reverse | CCAAACATGGCATCGATCTTGTCCTTGAAGGCG |
| Aac2 K215D | Forward | CGATTCTTTGGATCCTCTATTGTTGACTGGTTC |
| Aac2 K215D | Reverse | AACAATAGAGGATCCAAAGAATCGTACATACCG |

| | | |
|---|---|---|
| Aac2 A137D | Forward | TGGTGCTGATGGTGCCTTGTCATTACTATTTG |
| Aac2 A137D | Reverse | ACAAGGCACCATCAGCACCACCAGATGCCAAG |

**Appendix table S2: Primers used to generate human mutant constructs.**

| Target | Type | Sequence (5'-3') |
|---|---|---|
| ANT1 5' Flag (HindIII) | Forward | CCCAAGCTTATGGATTATAAAGATGATGACGATAAAATGGGTGATCACGCTTGGAG |
| ANT1 3' End (NotI) | Reverse | ATTTGCGGCCGCTTAGACATATTTTTTGATCTC |
| ANT1 L141E | Forward | GCTAGGACCAGGGAGGCTGCTGATGTGGGCAAG |
| ANT1 L141E | Reverse | ATCAGCAGCCTCCCTGGTCCTAGCAAAGTCCAGC |
| ANT1 L141F | Forward | GCTAGGACCAGGTTCGCTGCTGATGTGGGCAAG |
| ANT1 L141F | Reverse | ATCAGCAGCGAACCTGGTCCTAGCAAAGTCCAGC |

**Appendix table S3: Antibodies used in this study.**

| Antibodies | Source | Identifier |
|---|---|---|
| Flag, mouse monoclonal (M2) | Sigma-Aldrich | F3165 |
| Flag, mouse monoclonal (12C6c) | Developmental Studies Hybridoma Bank (DSHB) | RRID:AB_2890618 |
| Flag, rabbit polyclonal | Sigma-Aldrich | SAB4301135 |
| Aac2, mouse monoclonal (6H8) | (Panneels *et al*, 2003) | 6H8 |
| Aac2, rabbit polyclonal | (Claypool *et al*, 2008) | cmk167 |
| Tom70, rabbit polyclonal | (Riezman *et al*, 1983) | 7305 |
| Atp1/2, rabbit polyclonal | (Maccecchini *et al*, 1979) | UY3-T |
| Por1, rabbit polyclonal | (Daum *et al*, 1982) | 425 |
| Kgd1, rabbit polyclonal | (Glick *et al*, 1992) | 453-3 |
| Cor2, rabbit polyclonal | (Glick *et al*, 1992) | CC2-T |
| Cox1, rabbit polyclonal | (Dowhan *et al*, 1985) | DD2-4 |
| Cox2, rabbit polyclonal | (Poyton & Schatz, 1975) | 173 |
| Cox3, mouse monoclonal (DA5BC4) | Invitrogen | 459300 |
| Cox4, rabbit polyclonal | (Baile *et al*, 2013) | MGB65 |
| Rip1, rabbit polyclonal | (Baile *et al*, 2013) | MGB71 |
| Qcr6, rabbit polyclonal | (Baile *et al*, 2013) | MGB73 |
| Atp6, rabbit polyclonal | (Kabala *et al*, 2014) | N/A |
| Taz, rabbit polyclonal | (Claypool *et al*, 2006) | 4248 |
| Abf2, rabbit polyclonal | (Calzada *et al*, 2019) | 5477 |
| Tim54, rabbit polyclonal | This study | 7303 |
| β-actin, mouse monoclonal | Sigma-Aldrich | A5441; RRID:AB_476744 |
| GRP75, mouse monoclonal | Antibodies Incorporated | 75-127; RRID: AB_2120479 |
| ANT1, mouse monoclonal (1F3F11) | (Lu *et al*, 2017) | N/A |
| ANT2, rabbit polyclonal | (Acoba *et al*, 2021) | 5695 |
| ANT2/3, mouse monoclonal (5H7) | (Panneels *et al*, 2003) | N/A |
| NDUFB6, mouse monoclonal (21C11BC11) | Abcam | ab110244; RRID:AB_10865349 |
| SDHA, mouse monoclonal (2E3GC12FB2AE2) | Abcam | ab14715; RRID:AB_301433 |
| UQCRC2, mouse monoclonal (13G12) | Abcam | ab14745; RRID:AB_2213640 |
| MT-CO1, mouse monoclonal (1D6E1A8) | Thermo Fisher Scientific | 459600; RRID:AB_2532240 |
| COX4, rabbit polyclonal | Abcam | ab16056; RRID:AB_443304 |
| F1β, rabbit polyclonal | Proteintech | 17247-1-AP; RRID:AB_2061878 |
| TOM20, rabbit polyclonal | Proteintech | 11802-1-AP; RRID:AB_2207530 |

| | | |
|---|---|---|
| HRP-conjugated secondary, goat anti-rabbit IgG (H+L) | Thermo Fisher Scientific | 31460; RRID:AB_228341 |
| HRP-conjugated secondary, goat anti-mouse IgG (H+L) | Thermo Fisher Scientific | 62-6520; RRID:AB_2533947 |
| Daylight 650 conjugated secondary, goat anti-rabbit IgG (H+L) | Invitrogen | 84546 |
| Daylight 550 conjugated secondary, goat anti-mouse IgG (H+L) | Invitrogen | 84540 |

**Appendix table S4: Overview of the simulation setup and details.**

| Simulation Methods | System | Simulation length |
|---|---|---|
| Equilibrium<br>Pocket 2 CL prebound | WT | 1 X 1 μs |
| | L141F | 1 X 1 μs |
| | L141E | 1 X 1 μs |
| Equilibrium<br>Pocket 2 CL unbound | WT | 1 X 1 μs |
| | L141F | 1 X 1 μs |
| | L141E | 1 X 1 μs |
| Free Energy Perturbations (FEP) | WT (42 X 15 ns) | 4 X 0.63 μs = 2.52 μs |
| | L141F (42 X 15 ns) | 4 X 0.63 μs = 2.52 μs |
| | L141E (42 X 15 ns) | 4 X 0.63 μs = 2.52 μs |
| | Ligand (31 X 15 ns) | 4 X 0.465 μs = 1.86 μs |
| | | Total = 15.42 μs |

**References:**


Acoba MG, Alpergin ESS, Renuse S, Fernández-del-Río L, Lu Y-W, Khalimonchuk O, Clarke CF, Pandey A, Wolfgang MJ & Claypool SM (2021) The mitochondrial carrier SFXN1 is critical for complex III integrity and cellular metabolism. *Cell Rep* 34: 108869

Baile MG, Whited K & Claypool SM (2013) Deacylation on the matrix side of the mitochondrial inner membrane regulates cardiolipin remodeling. *Mol Biol Cell* 24: 2008–2020

Calzada E, Avery E, Sam PN, Modak A, Wang C, McCaffery JM, Han X, Alder NN & Claypool SM (2019) Phosphatidylethanolamine made in the inner mitochondrial membrane is essential for yeast cytochrome bc1 complex function. *Nat Commun* 10: 1432

Claypool SM, McCaffery JM & Koehler CM (2006) Mitochondrial mislocalization and altered assembly of a cluster of Barth syndrome mutant tafazzins. *J Cell Biol* 174: 379–390

Claypool SM, Oktay Y, Boontheung P, Loo JA & Koehler CM (2008) Cardiolipin defines the interactome of the major ADP/ATP carrier protein of the mitochondrial inner membrane. *J Cell Biol* 182: 937–950

Daum G, Böhni PC & Schatz G (1982) Import of proteins into mitochondria. Cytochrome b2 and cytochrome c peroxidase are located in the intermembrane space of yeast mitochondria. *J Biol Chem* 257: 13028–13033

Dowhan W, Bibus CR & Schatz G (1985) The cytoplasmically-made subunit IV is necessary for assembly of cytochrome c oxidase in yeast. *EMBO J* 4: 179–184

Glick S, Brandt A, Cunningham K & Hallberg RL (1992) Cytochromes c1 and b2 are sorted to the intermembrane space of yeast mitochondria by a stop-transfer mechanism. *Cell* 69: 809–822

Kabala AM, Lasserre J-P, Ackerman SH, di Rago J-P & Kucharczyk R (2014) Defining the impact on yeast ATP synthase of two pathogenic human mitochondrial DNA mutations, T9185C and T9191C. *Biochimie* 100: 200–206

Lu Y-W, Acoba MG, Selvaraju K, Huang T-C, Nirujogi RS, Sathe G, Pandey A & Claypool SM (2017) Human adenine nucleotide translocases physically and functionally interact with respirasomes. *Mol Biol Cell* 28: 1489–1506

Maccecchini ML, Rudin Y, Blobel G & Schatz G (1979) Import of proteins into mitochondria: precursor forms of the extramitochondrially made F1-ATPase subunits in yeast. *Proc Natl Acad Sci* 76: 343–347

Panneels V, Schüssler U, Costagliola S & Sinning I (2003) Choline head groups stabilize the matrix loop regions of the ATP/ADP carrier ScAAC2. *Biochem Biophys Res Commun* 300: 65–74

Poyton RO & Schatz G (1975) Cytochrome c oxidase from bakers' yeast. III. Physical characterization of isolated subunits and chemical evidence for two different classes of polypeptides. *J Biol Chem* 250: 752–761

Riezman H, Hay R, Witte C, Nelson N & Schatz G (1983) Yeast mitochondrial outer membrane specifically binds cytoplasmically-synthesized precursors of mitochondrial proteins. *EMBO J* 2: 1113–1118
