# Supplementary figures and images for: Functional diversity among cardiolipin binding sites on the mitochondrial ADP/ATP carrier
Source: EMBO J. 2024 Jun 5;43(14):2979–3008. doi: 10.1038/s44318-024-00132-2 (PMC11251061; doi:10.1038/s44318-024-00132-2)

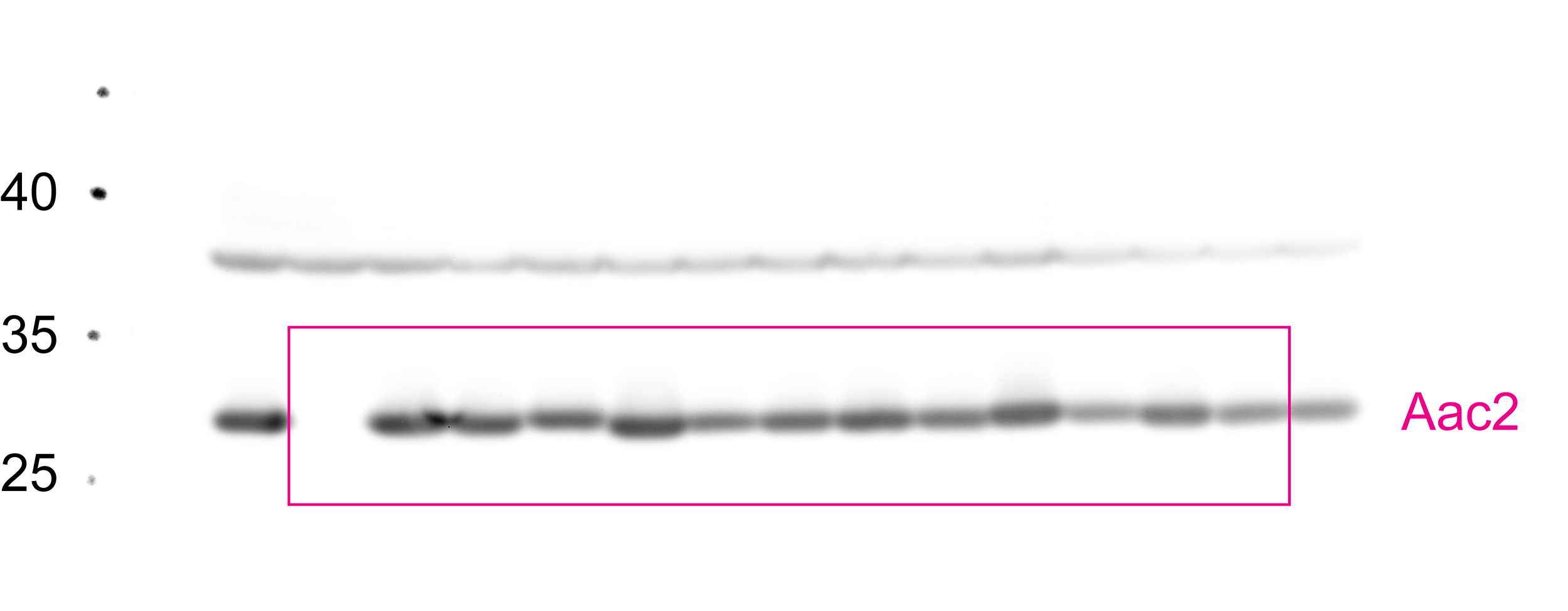

Supplement: Supplementary file 3 — Source data Fig. 1 [file 44318_2024_132_MOESM3_ESM.zip › Fig 1/1C/Aac2.tif]

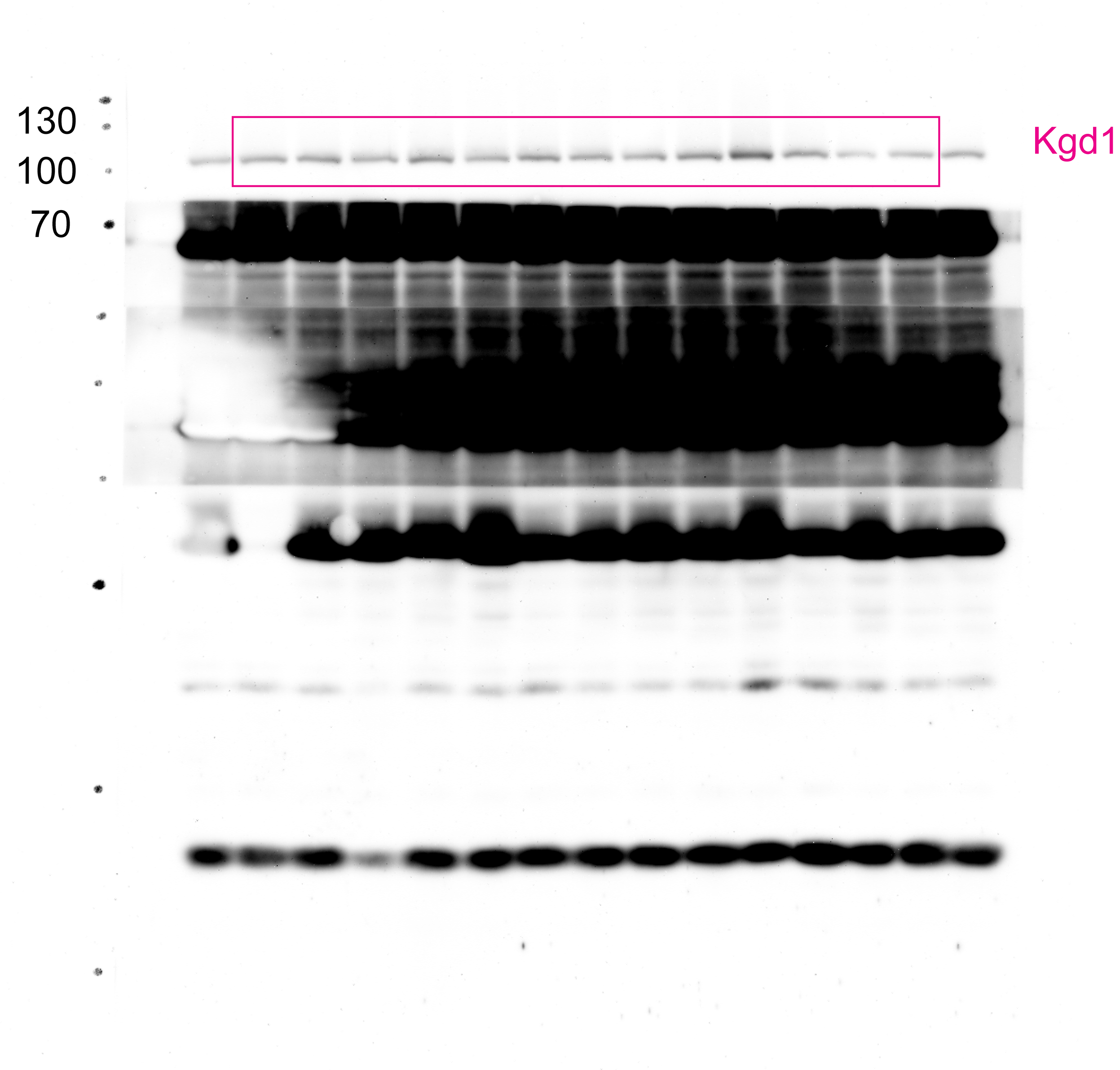

Supplement: Supplementary file 3 — Source data Fig. 1 [file 44318_2024_132_MOESM3_ESM.zip › Fig 1/1C/Kgd1.tif]

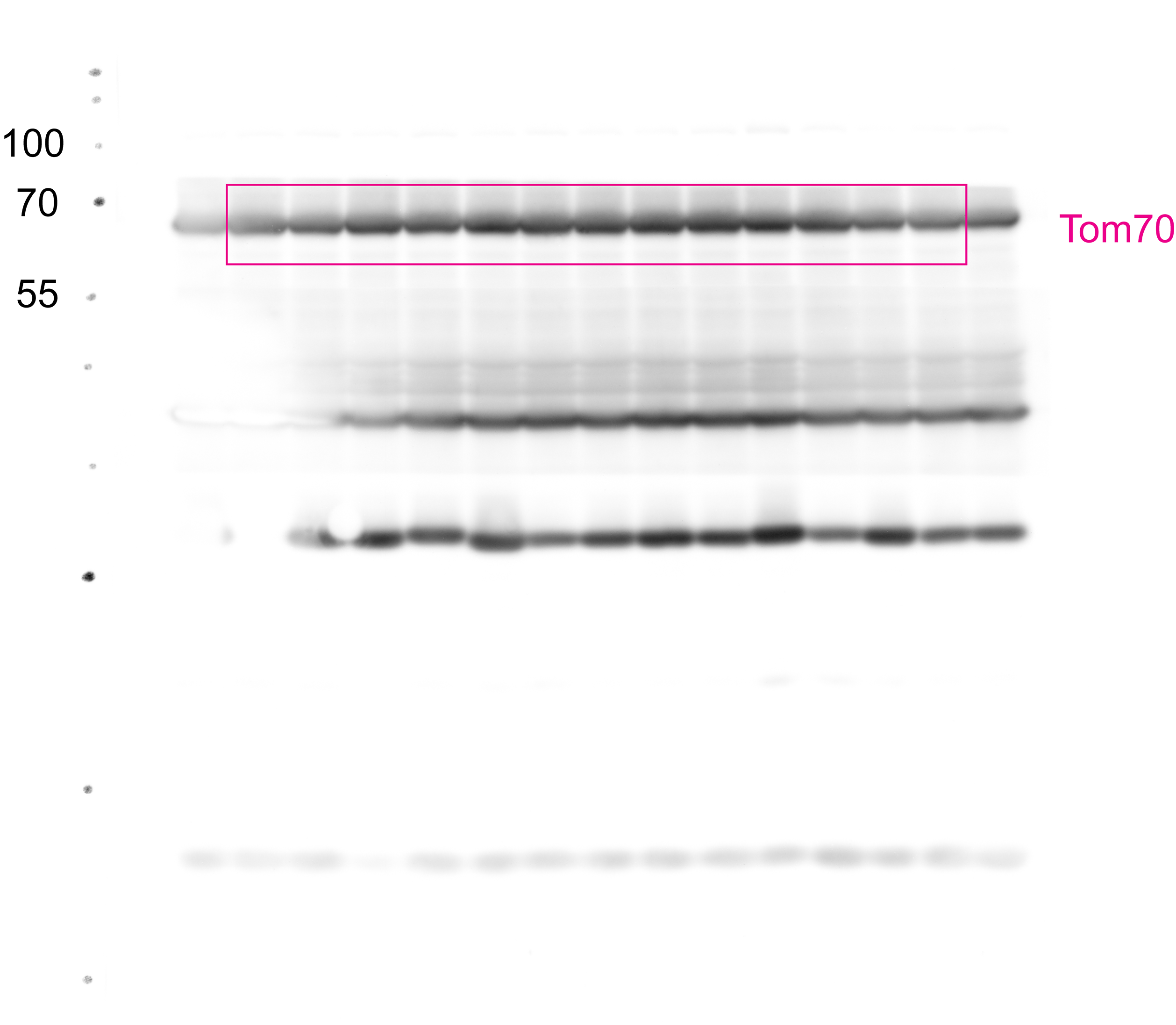

Supplement: Supplementary file 3 — Source data Fig. 1 [file 44318_2024_132_MOESM3_ESM.zip › Fig 1/1C/Tom70.tif]

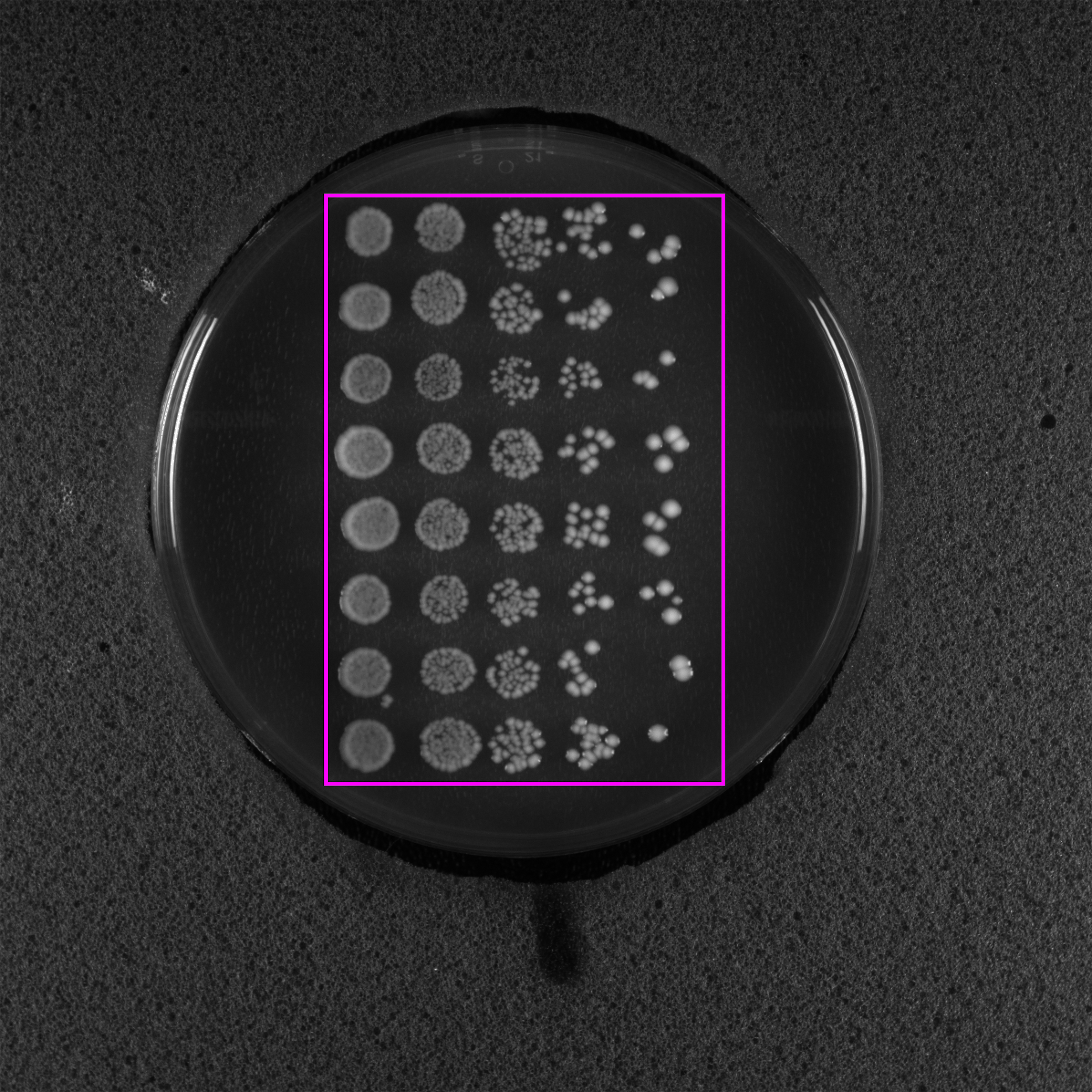

Supplement: Supplementary file 3 — Source data Fig. 1 [file 44318_2024_132_MOESM3_ESM.zip › Fig 1/1D/30C, YPD, 3d (1- aac2-172).tif]

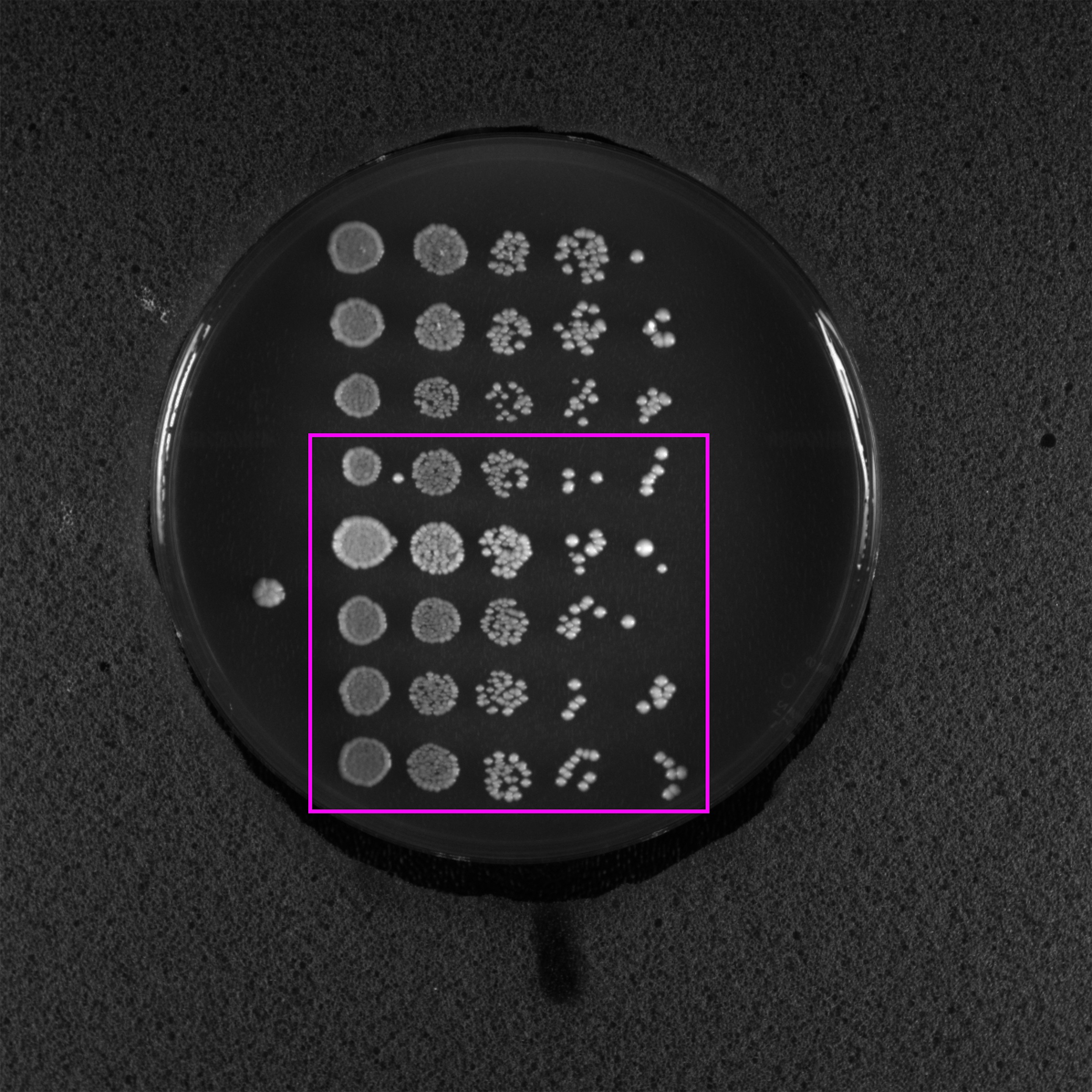

Supplement: Supplementary file 3 — Source data Fig. 1 [file 44318_2024_132_MOESM3_ESM.zip › Fig 1/1D/30C, YPD, 3d (2- 191-69267).tif]

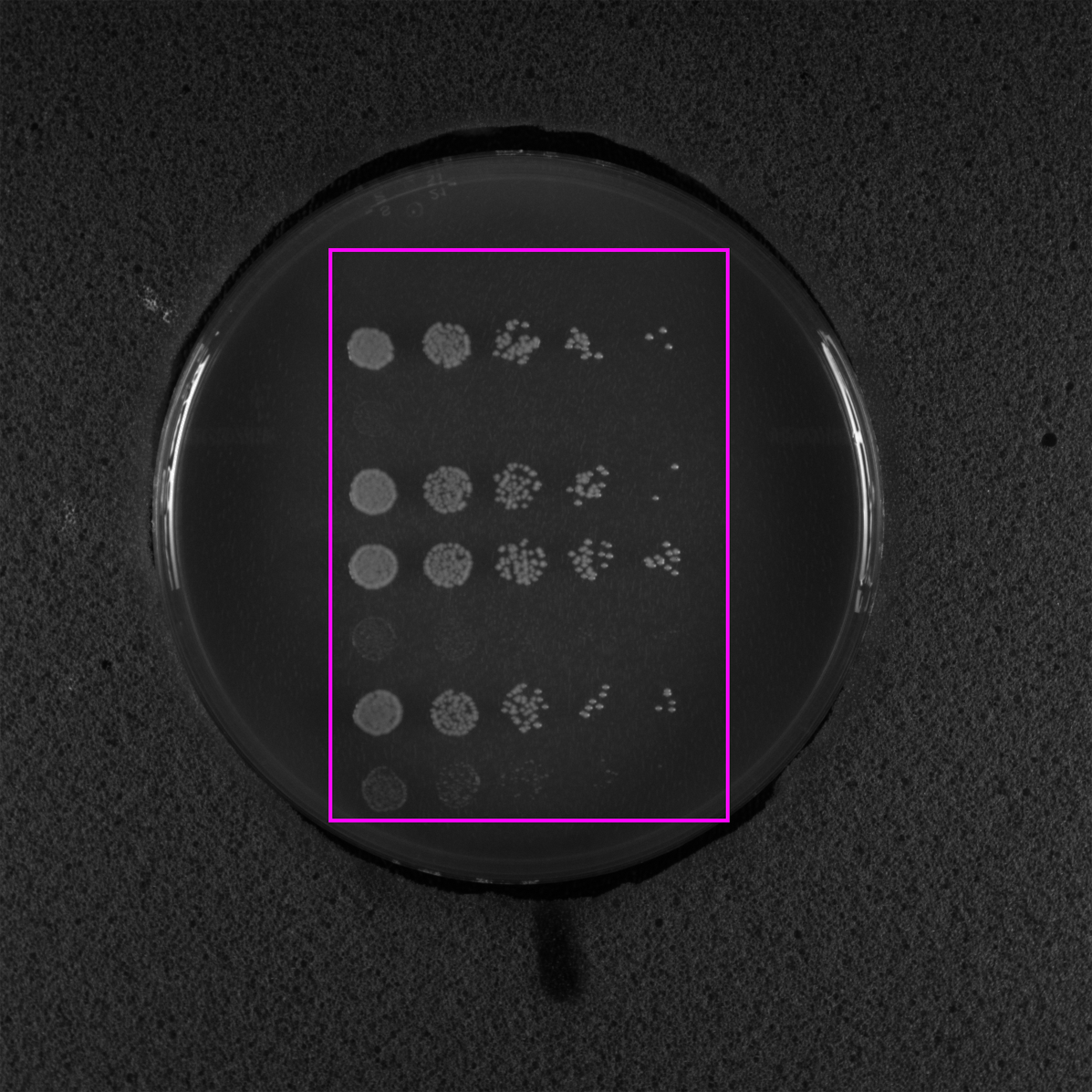

Supplement: Supplementary file 3 — Source data Fig. 1 [file 44318_2024_132_MOESM3_ESM.zip › Fig 1/1D/30C, YPEG, 3d (1- aac2-172).tif]

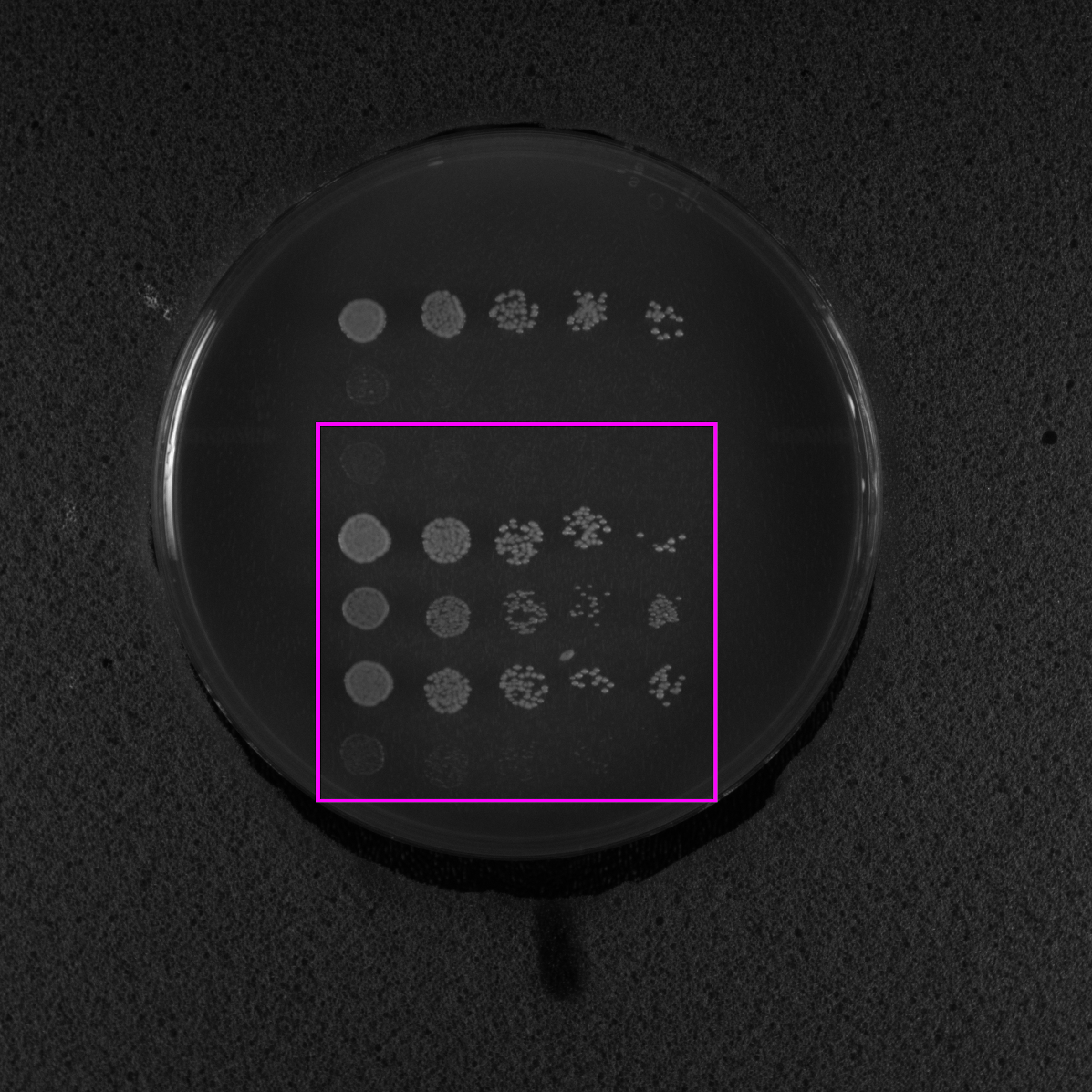

Supplement: Supplementary file 3 — Source data Fig. 1 [file 44318_2024_132_MOESM3_ESM.zip › Fig 1/1D/30C, YPEG, 3d (2- 191-69267).tif]

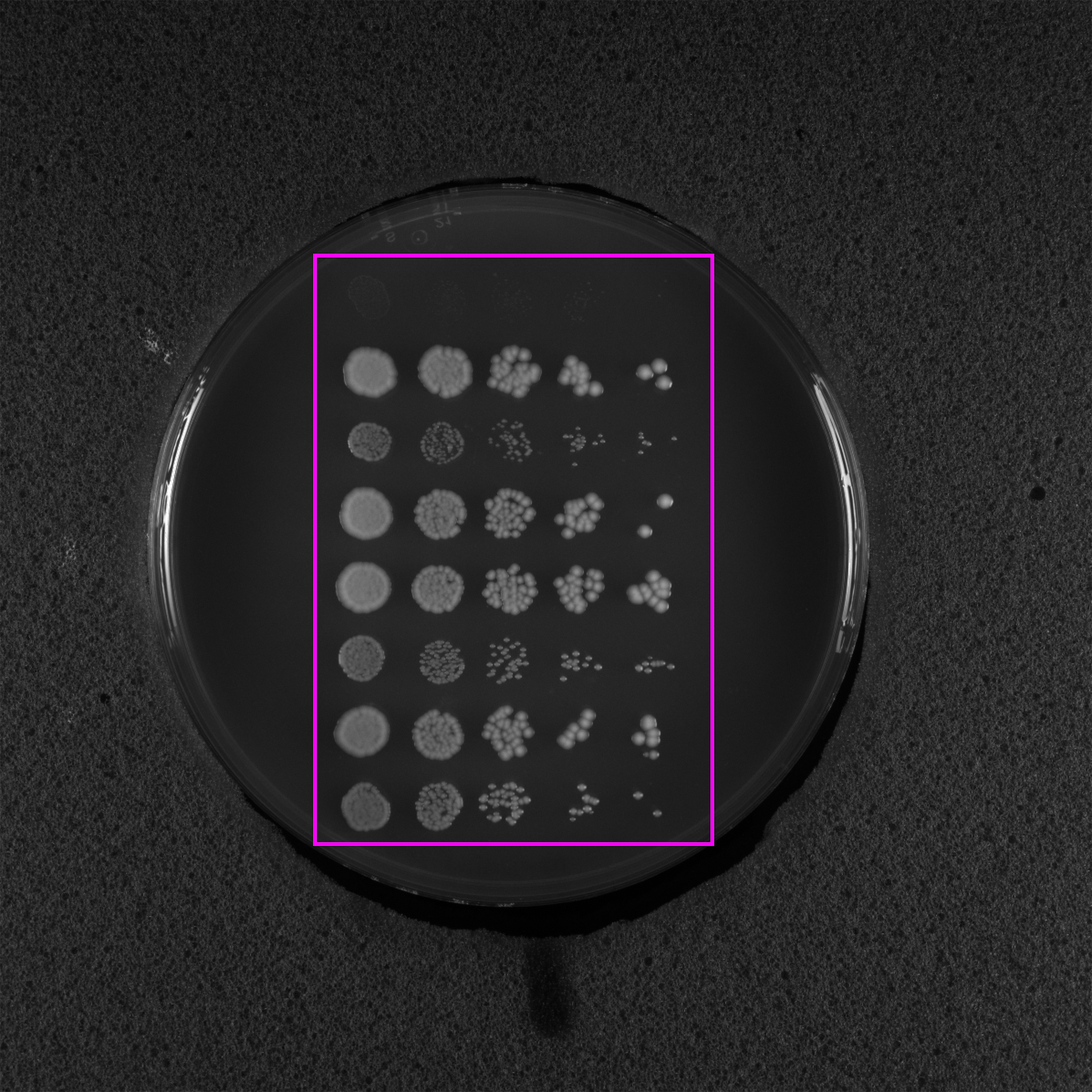

Supplement: Supplementary file 3 — Source data Fig. 1 [file 44318_2024_132_MOESM3_ESM.zip › Fig 1/1D/30C, YPEG, 5d (1- aac2-172).tif]

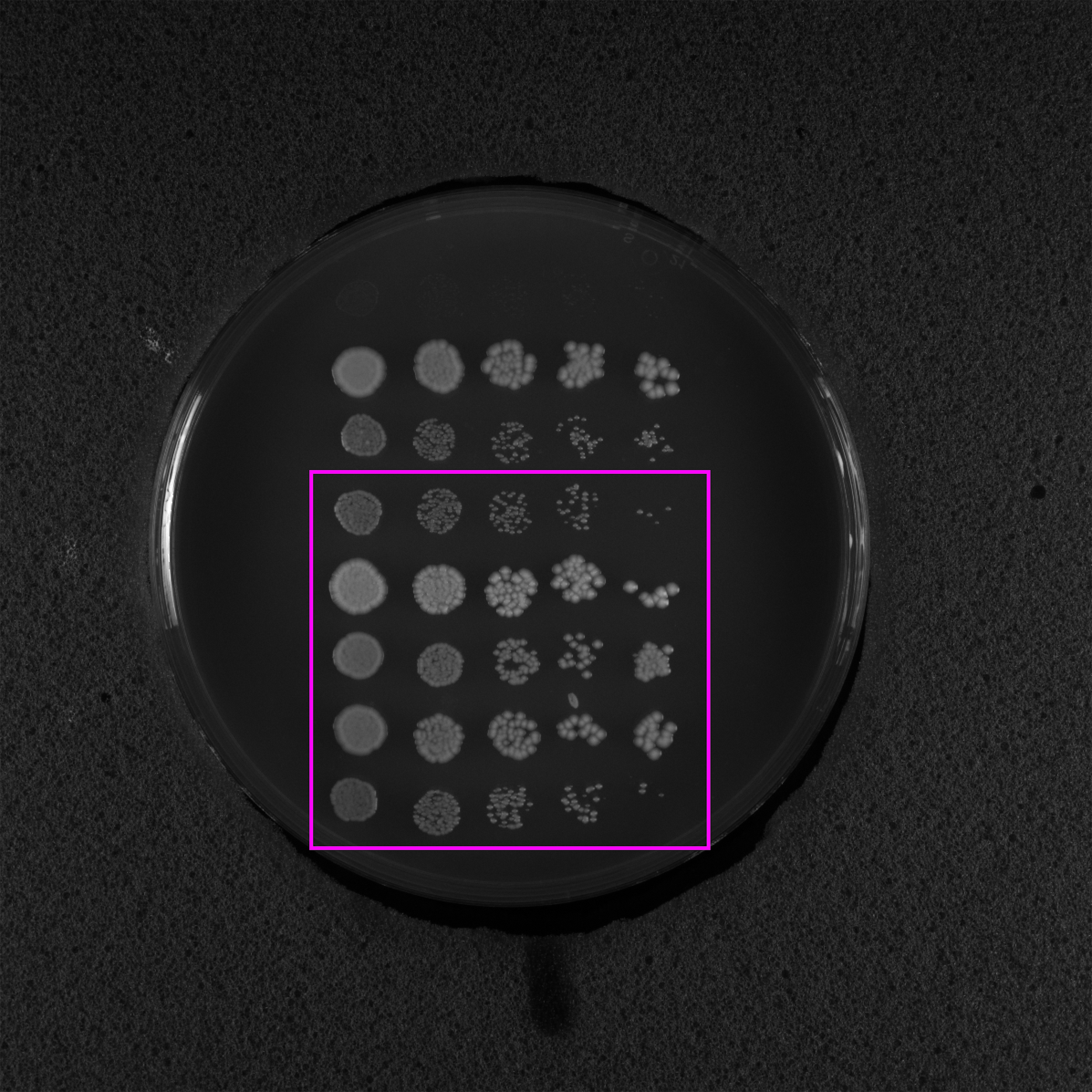

Supplement: Supplementary file 3 — Source data Fig. 1 [file 44318_2024_132_MOESM3_ESM.zip › Fig 1/1D/30C, YPEG, 5d (2- 191-69267).tif]

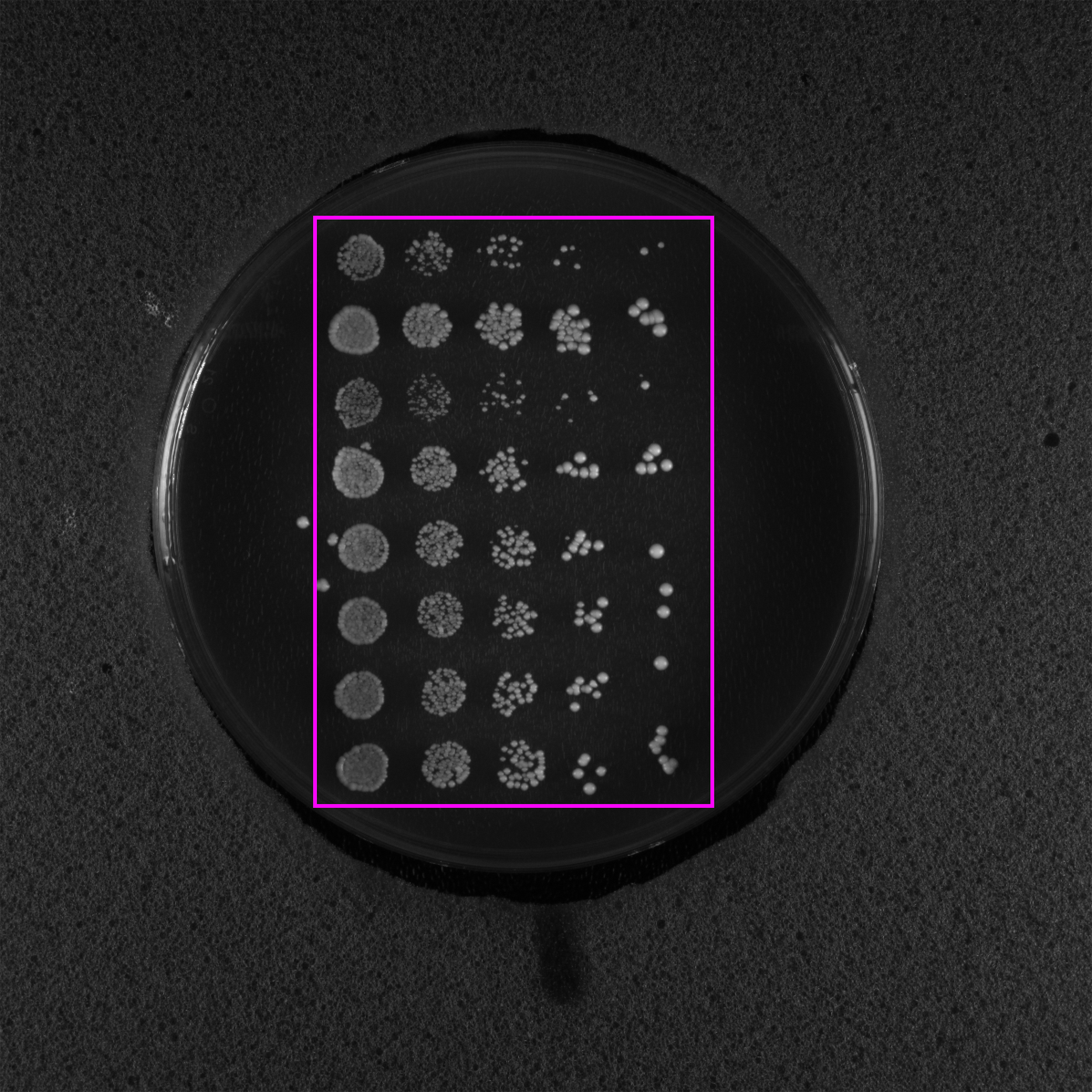

Supplement: Supplementary file 3 — Source data Fig. 1 [file 44318_2024_132_MOESM3_ESM.zip › Fig 1/1D/37C, YPD, 3d (1- aac2-172).tif]

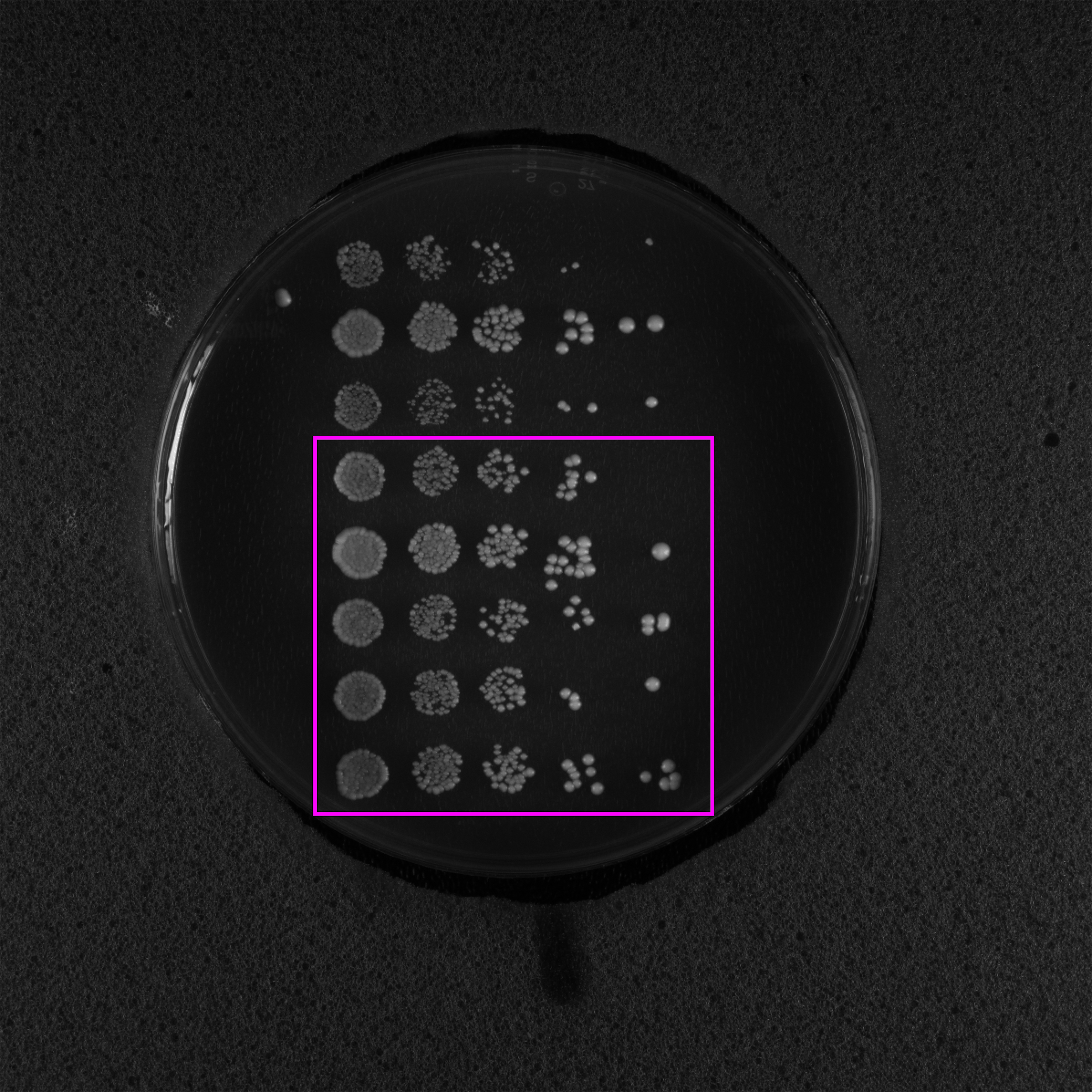

Supplement: Supplementary file 3 — Source data Fig. 1 [file 44318_2024_132_MOESM3_ESM.zip › Fig 1/1D/37C, YPD, 3d (2-191-69267).tif]

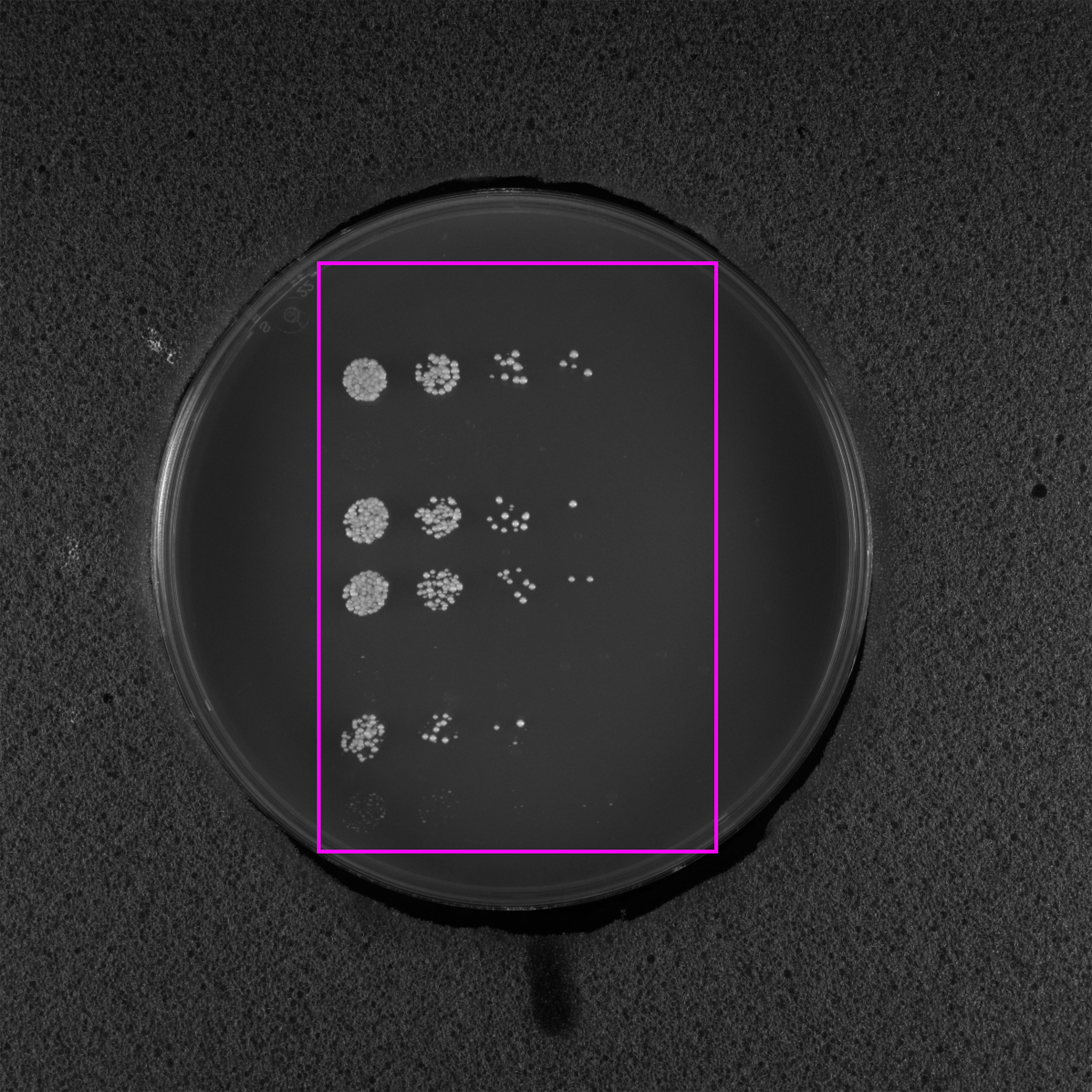

Supplement: Supplementary file 3 — Source data Fig. 1 [file 44318_2024_132_MOESM3_ESM.zip › Fig 1/1D/37C, YPEG, 5d (1- aac2-172).tif]

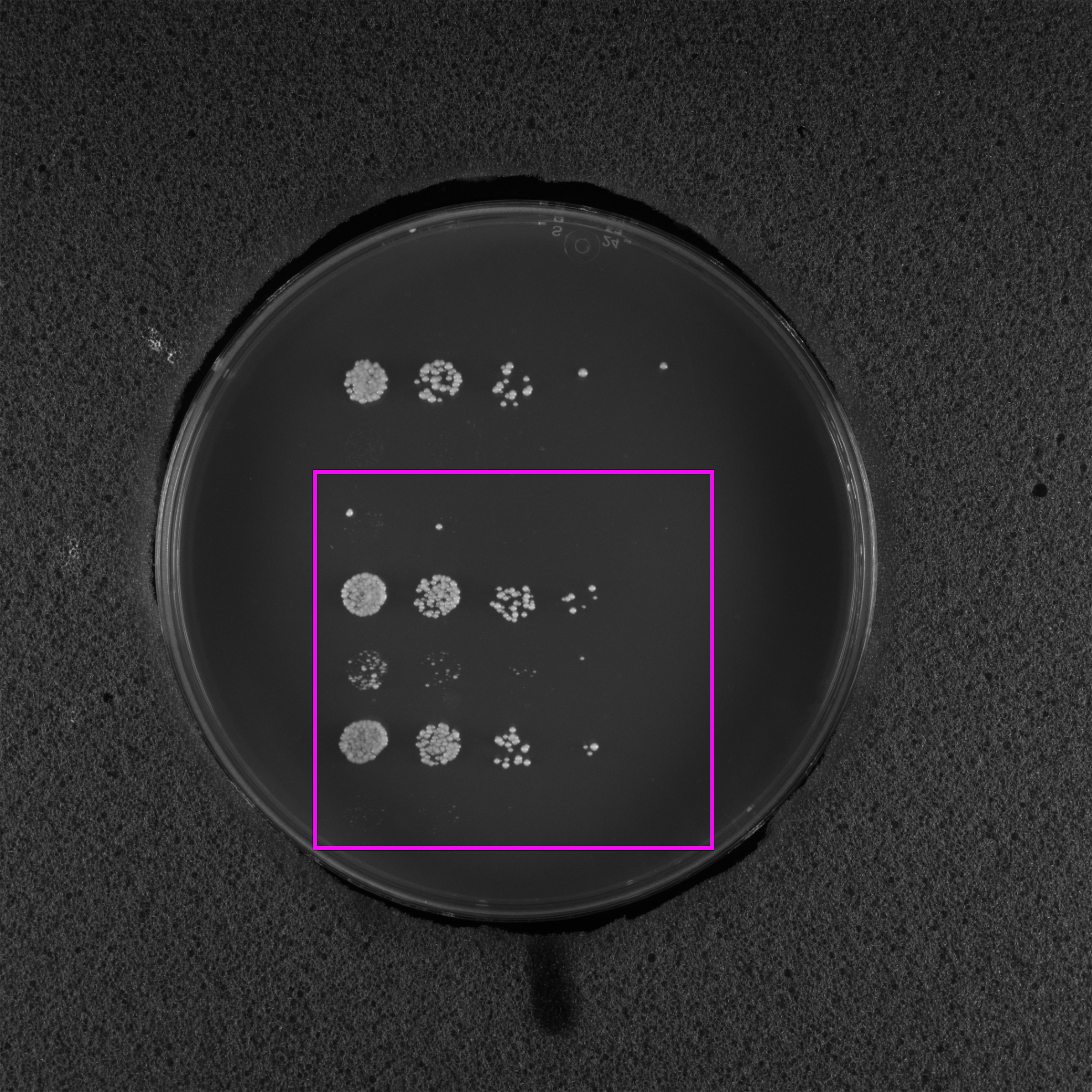

Supplement: Supplementary file 3 — Source data Fig. 1 [file 44318_2024_132_MOESM3_ESM.zip › Fig 1/1D/37C, YPEG, 5d (2- 191-69267).tif]

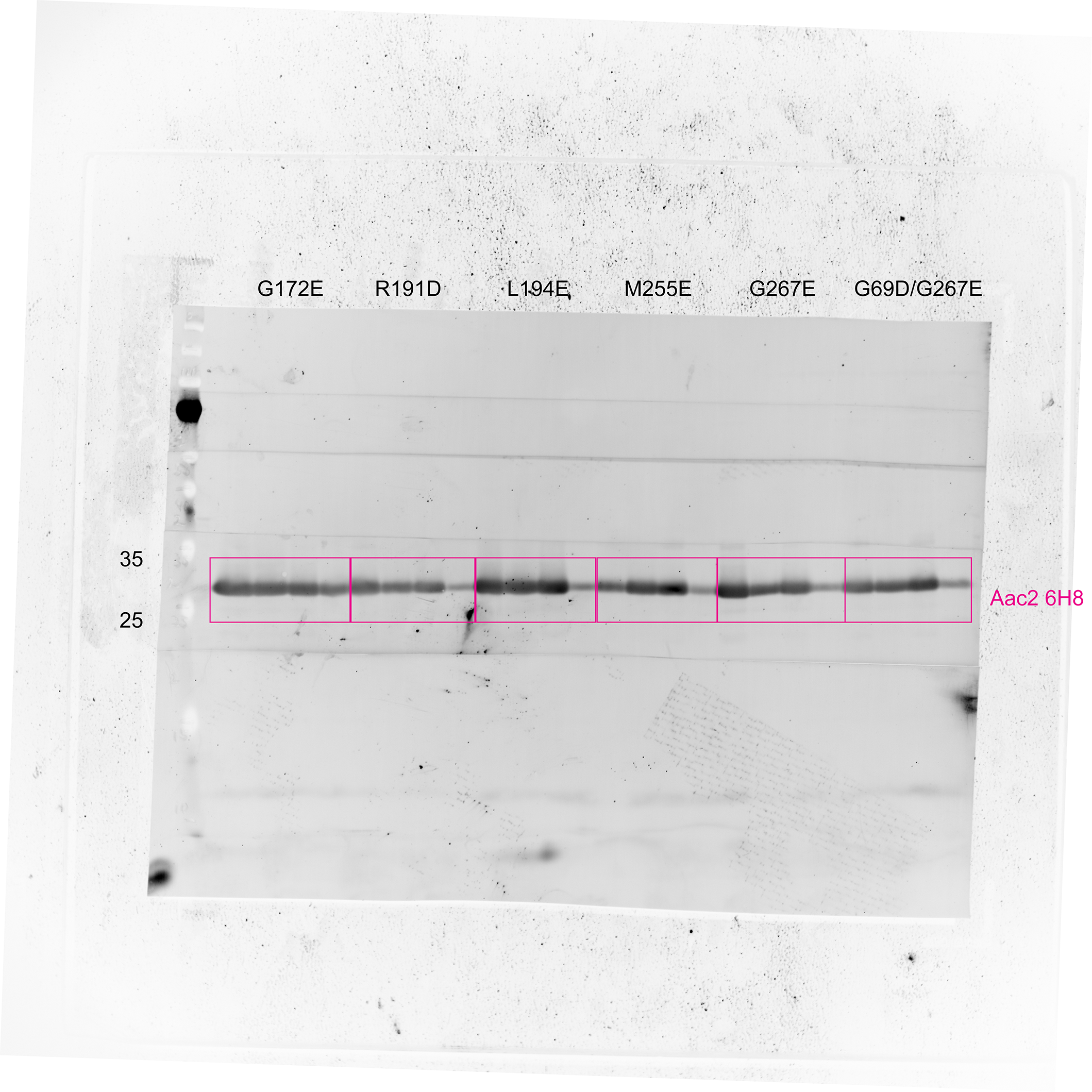

Supplement: Supplementary file 3 — Source data Fig. 1 [file 44318_2024_132_MOESM3_ESM.zip › Fig 1/1E/Aac2 6H8 (L155E-G69DG267E).tif]

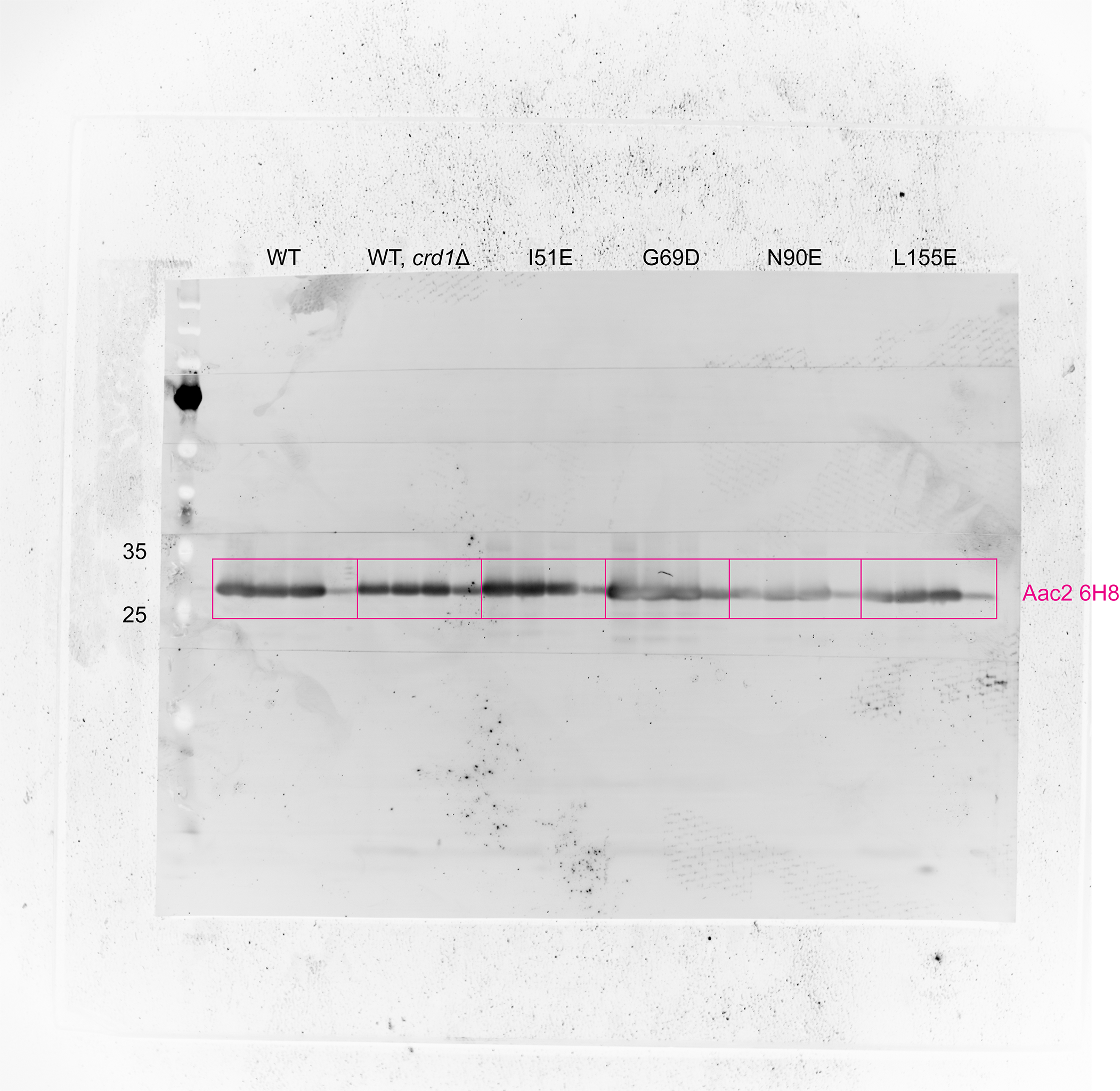

Supplement: Supplementary file 3 — Source data Fig. 1 [file 44318_2024_132_MOESM3_ESM.zip › Fig 1/1E/Aac2 6H8 (WT-N90E).tif]

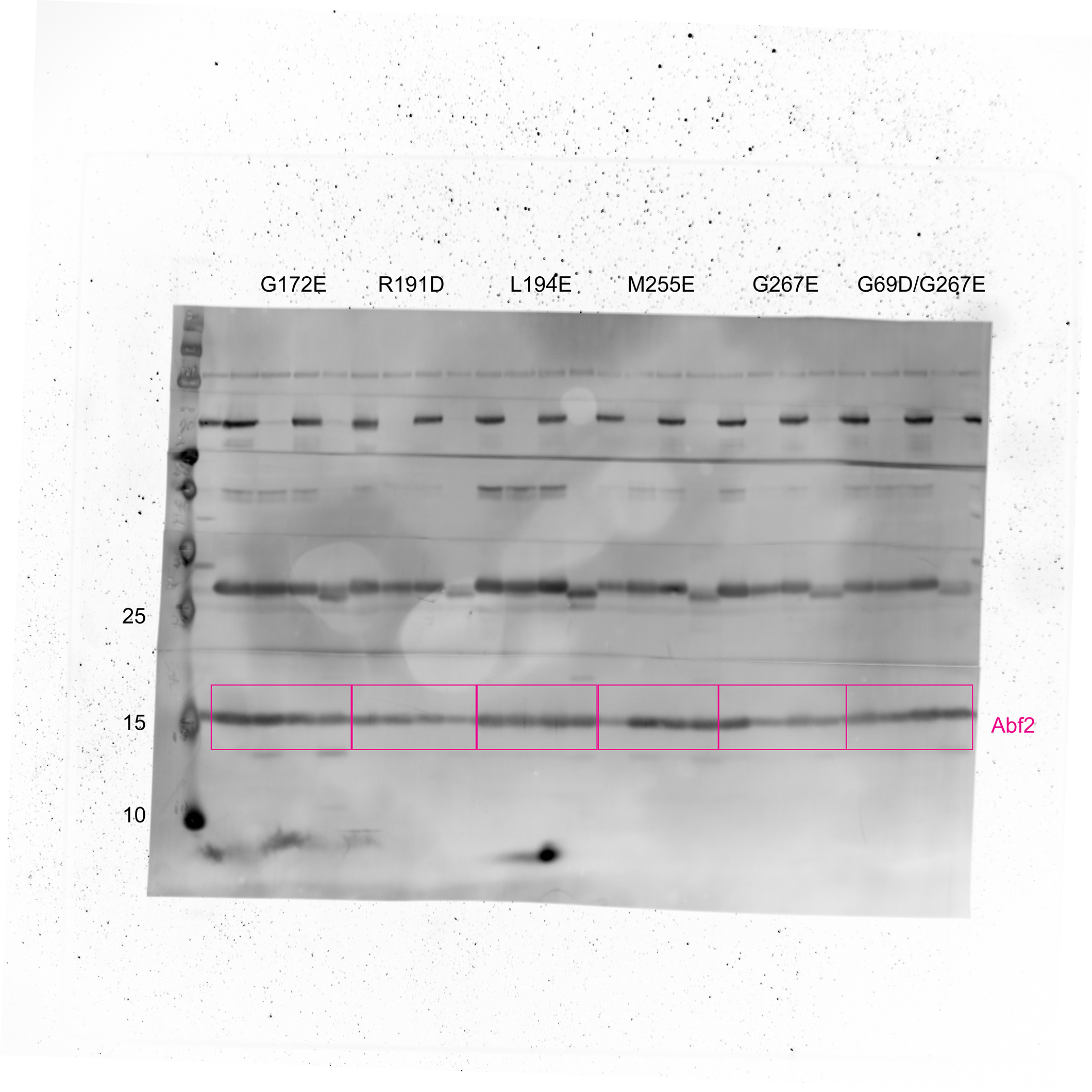

Supplement: Supplementary file 3 — Source data Fig. 1 [file 44318_2024_132_MOESM3_ESM.zip › Fig 1/1E/Abf2 (L155E-G69DG267E).tif]

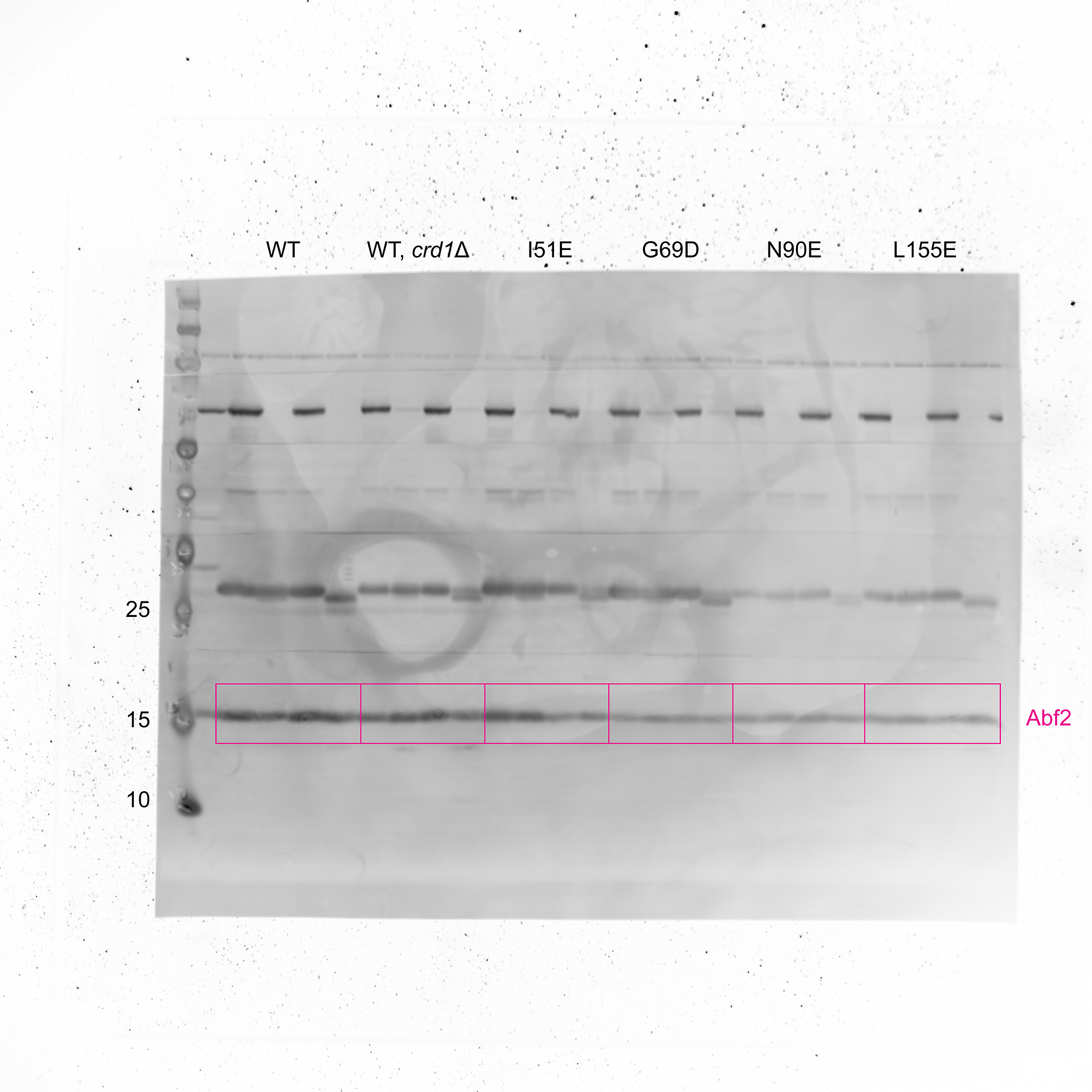

Supplement: Supplementary file 3 — Source data Fig. 1 [file 44318_2024_132_MOESM3_ESM.zip › Fig 1/1E/Abf2 (WT-N90E).tif]

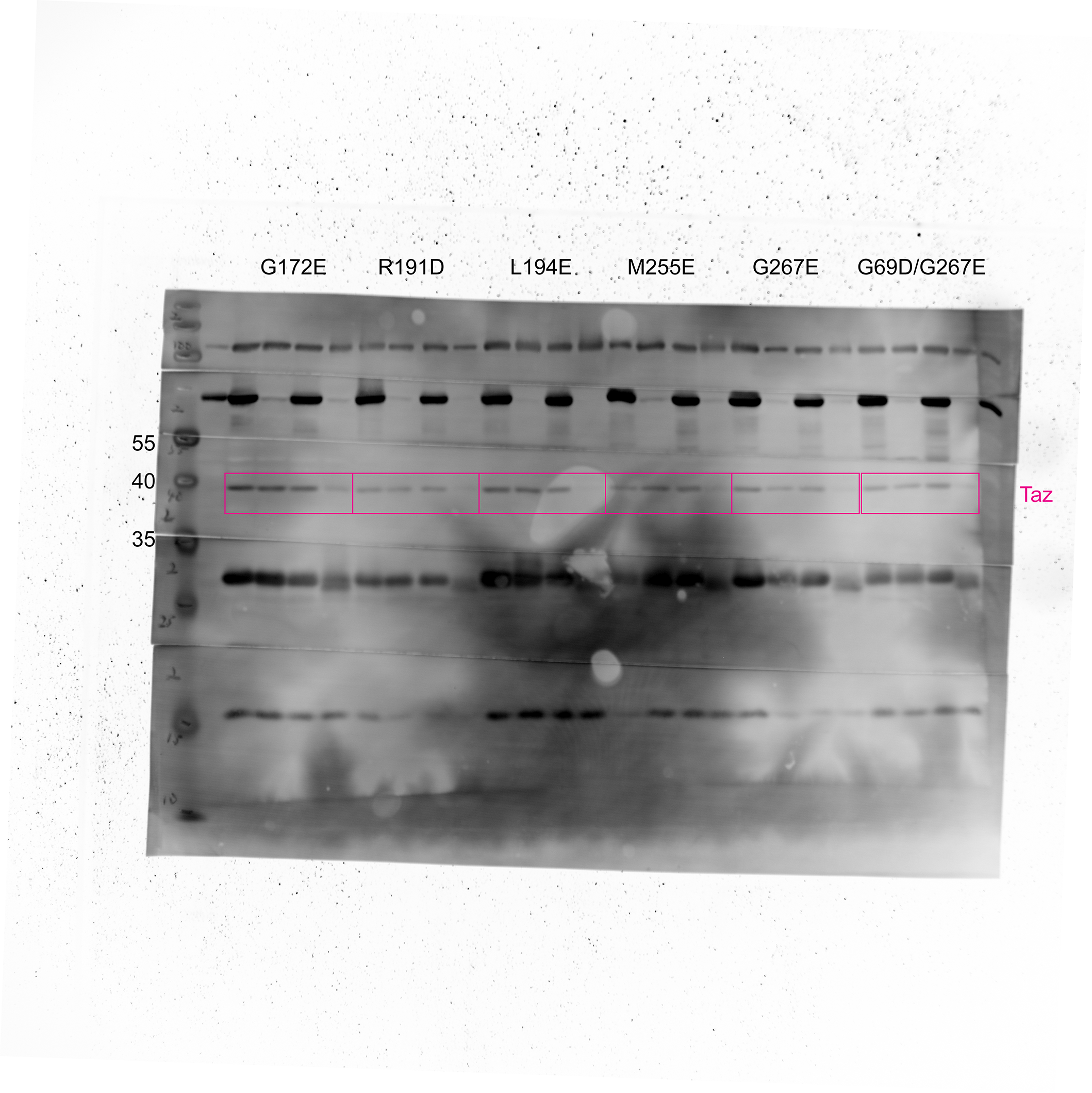

Supplement: Supplementary file 3 — Source data Fig. 1 [file 44318_2024_132_MOESM3_ESM.zip › Fig 1/1E/Taz (G172E-G69DG267E).tif]

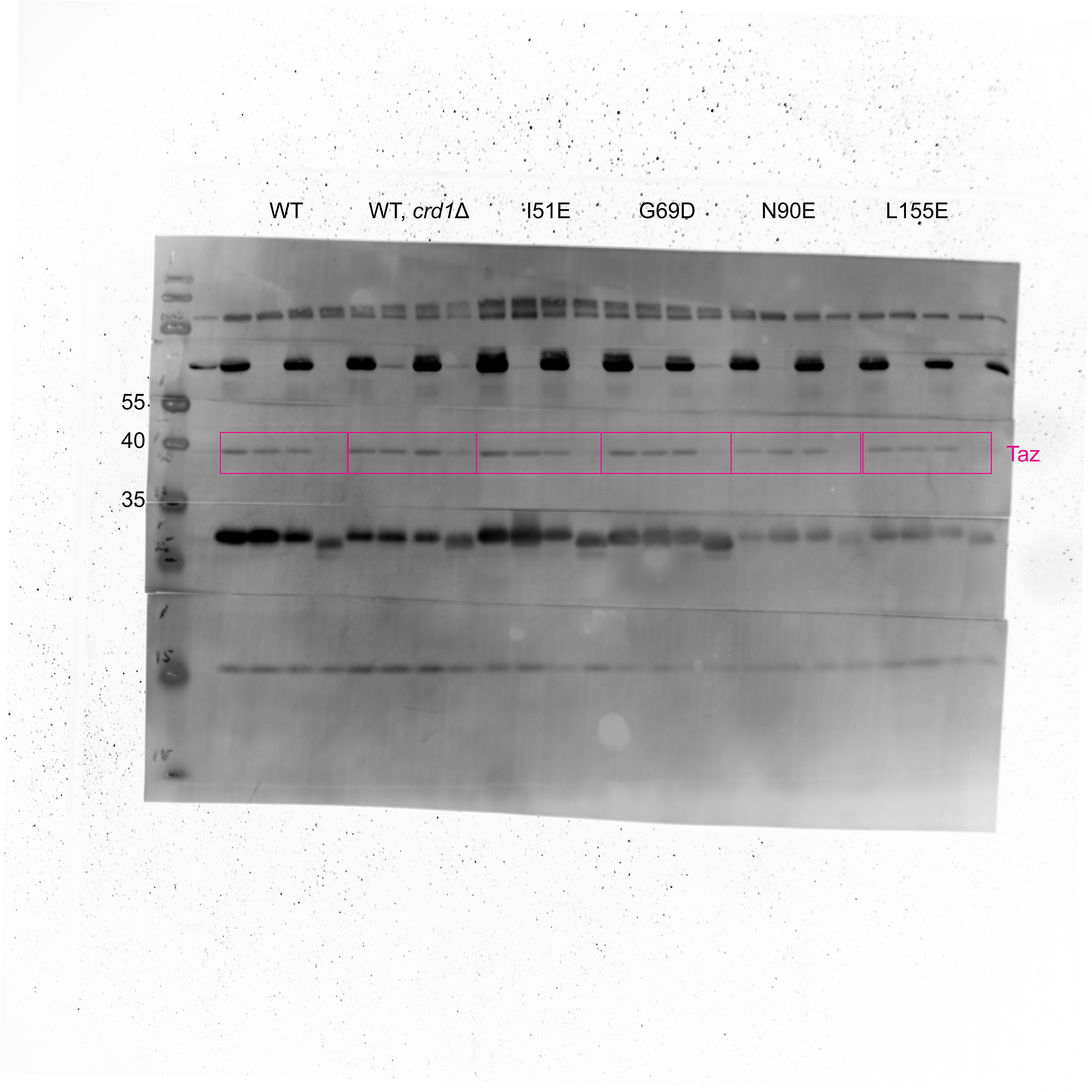

Supplement: Supplementary file 3 — Source data Fig. 1 [file 44318_2024_132_MOESM3_ESM.zip › Fig 1/1E/Taz (WT-L155E).tif]

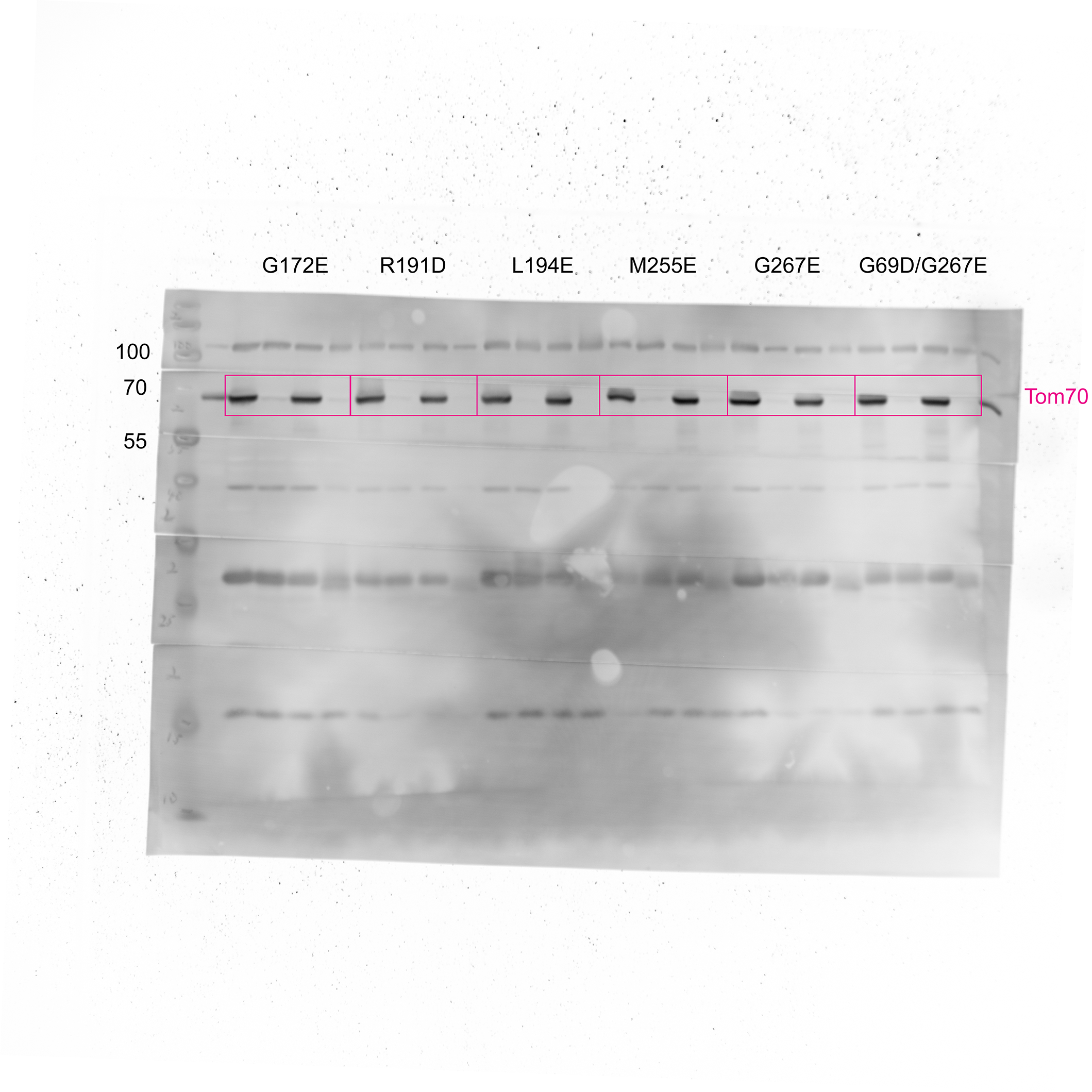

Supplement: Supplementary file 3 — Source data Fig. 1 [file 44318_2024_132_MOESM3_ESM.zip › Fig 1/1E/Tom70 (G172E-G69DG267E).tif]

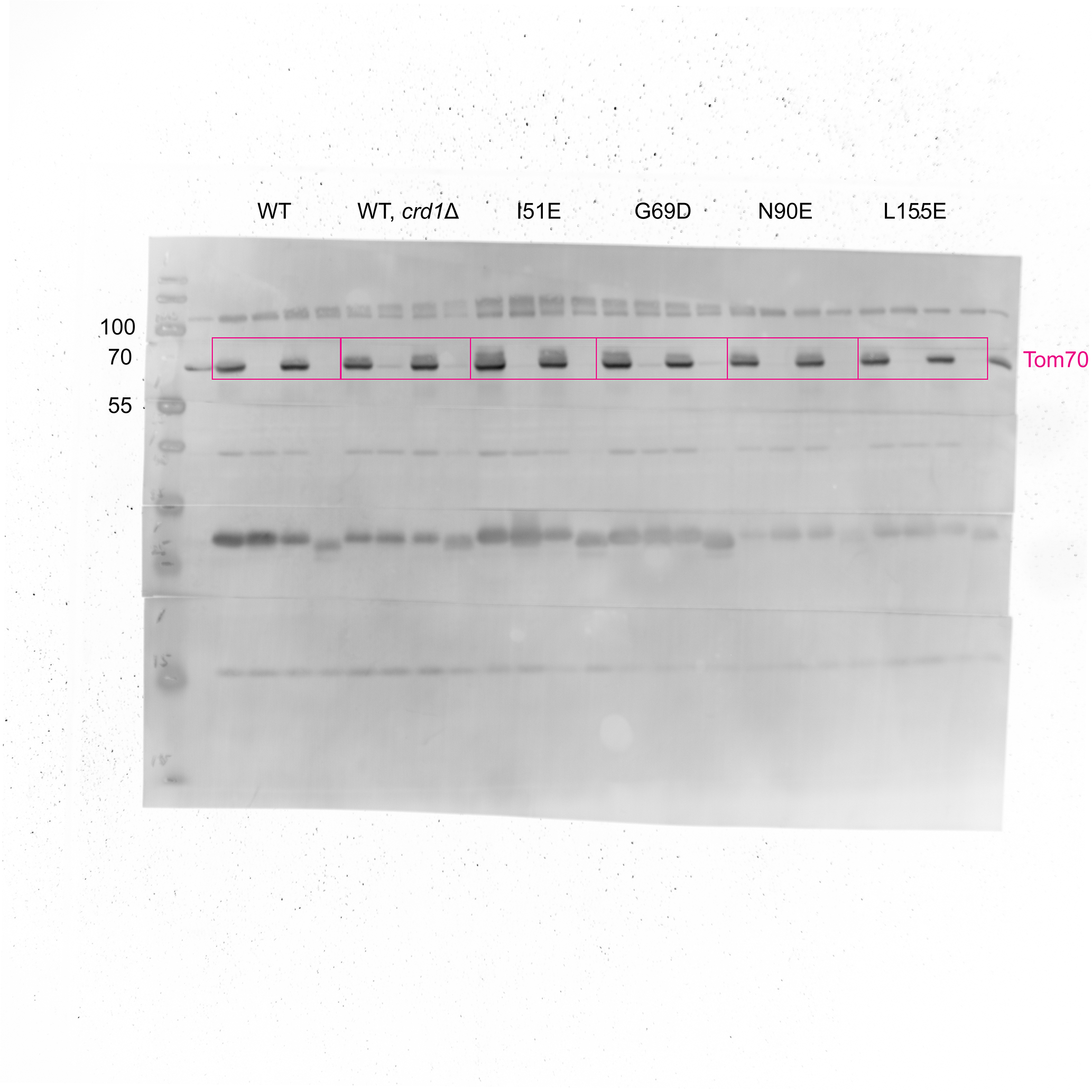

Supplement: Supplementary file 3 — Source data Fig. 1 [file 44318_2024_132_MOESM3_ESM.zip › Fig 1/1E/Tom70 (WT-L155E).tif]

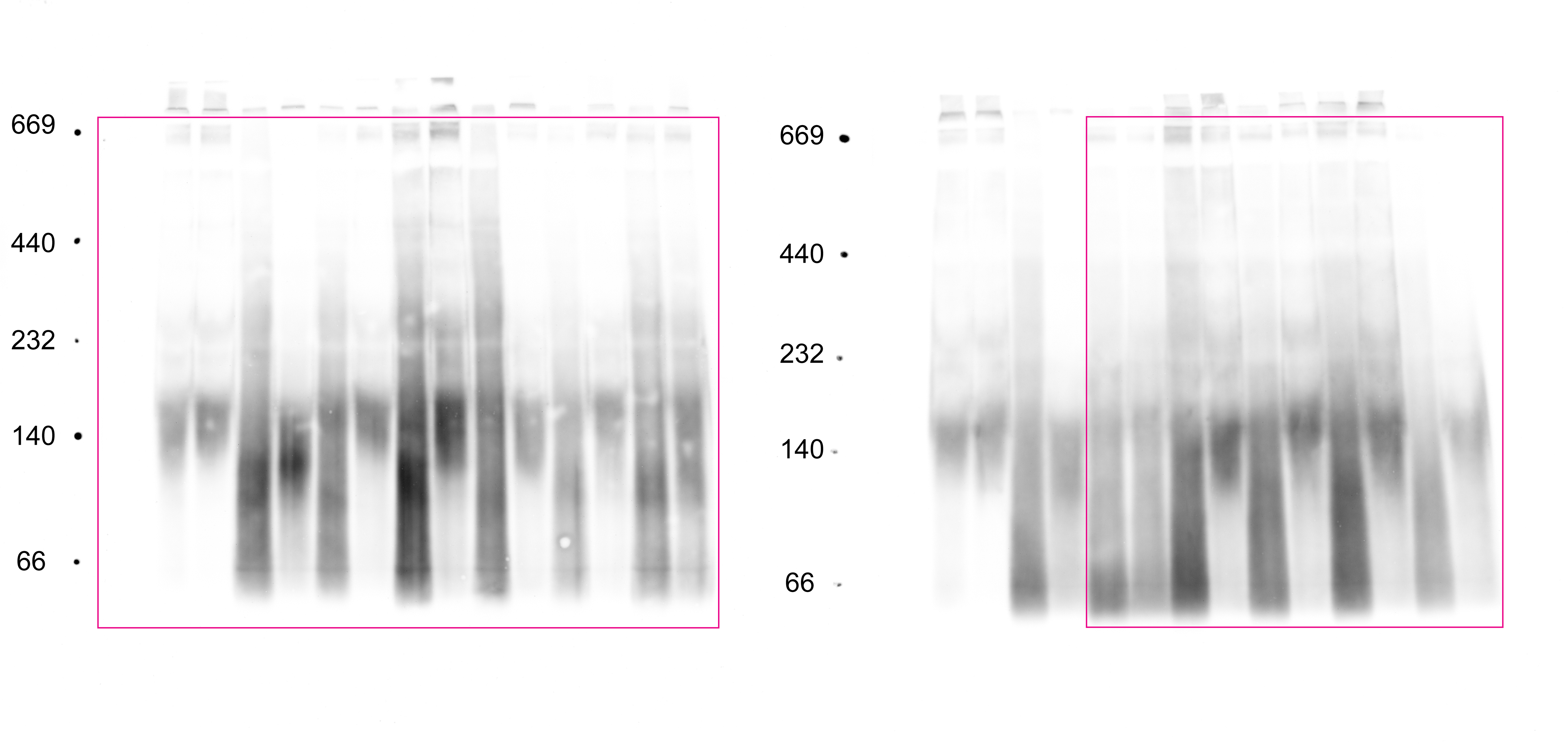

Supplement: Supplementary file 5 — Source data Fig. 3 [file 44318_2024_132_MOESM5_ESM.zip › Fig 3/3A/3A, +CATR.tif]

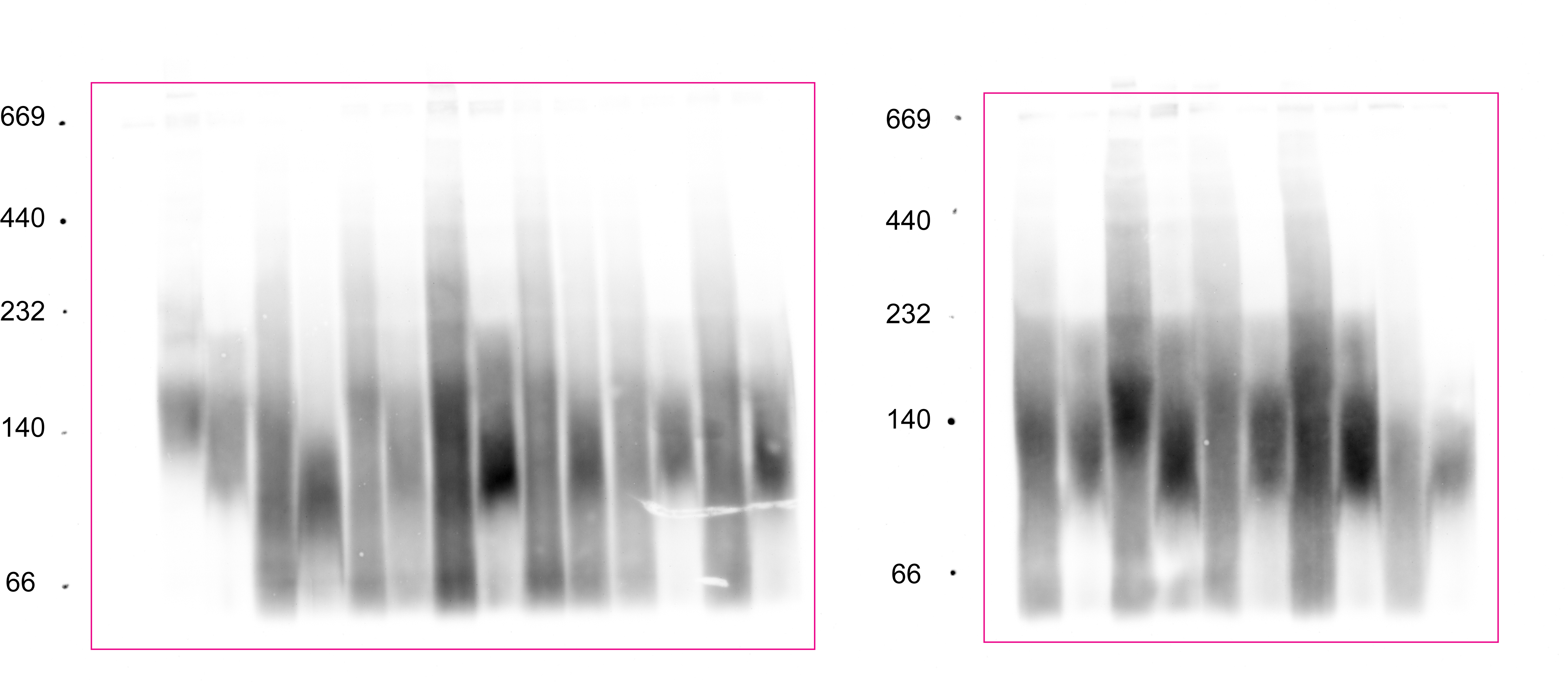

Supplement: Supplementary file 5 — Source data Fig. 3 [file 44318_2024_132_MOESM5_ESM.zip › Fig 3/3B/3B, +BKA.tif]

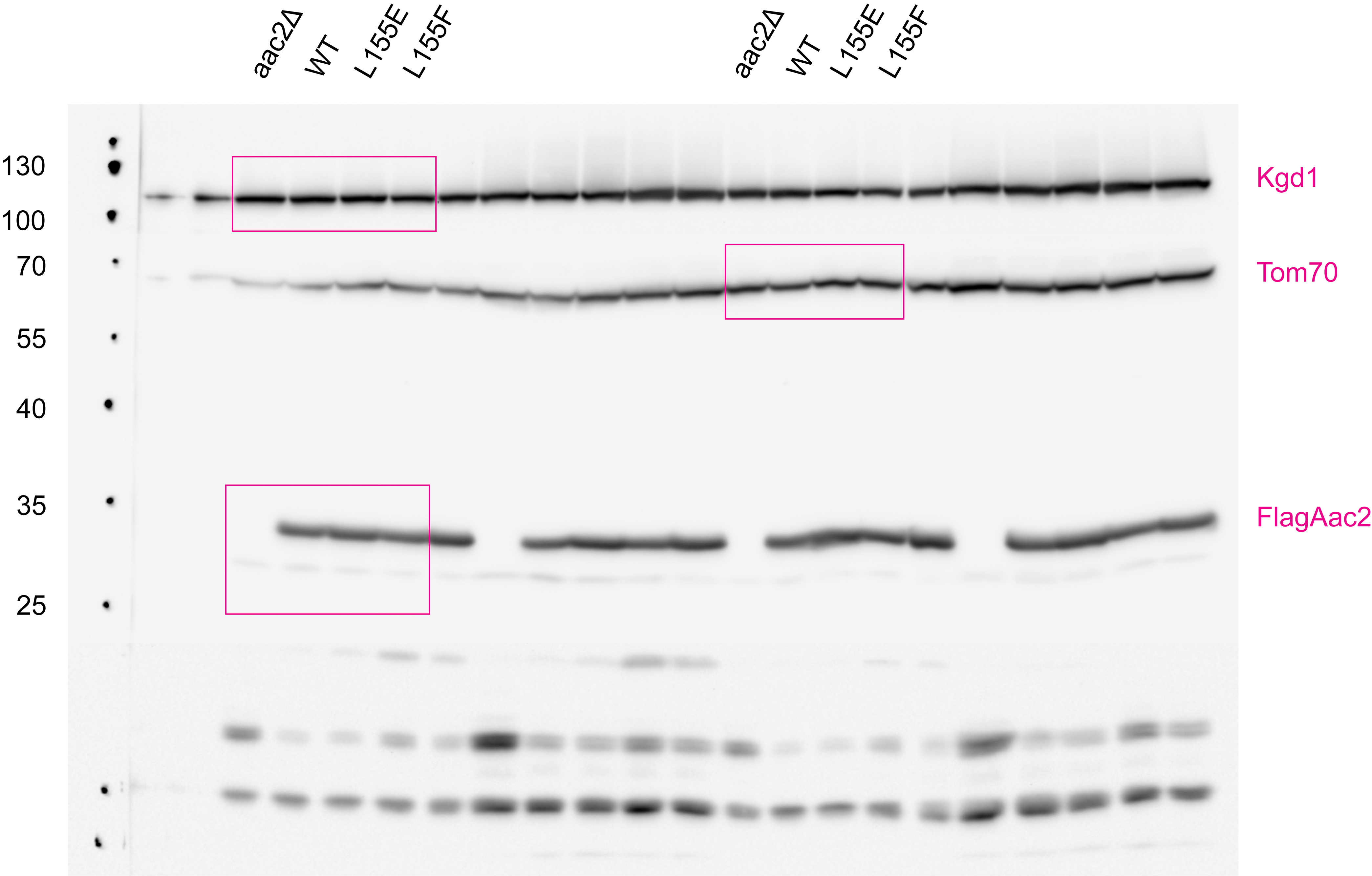

Supplement: Supplementary file 7 — Source data Fig. 5 [file 44318_2024_132_MOESM7_ESM.zip › Fig 5/5C/western.tif]

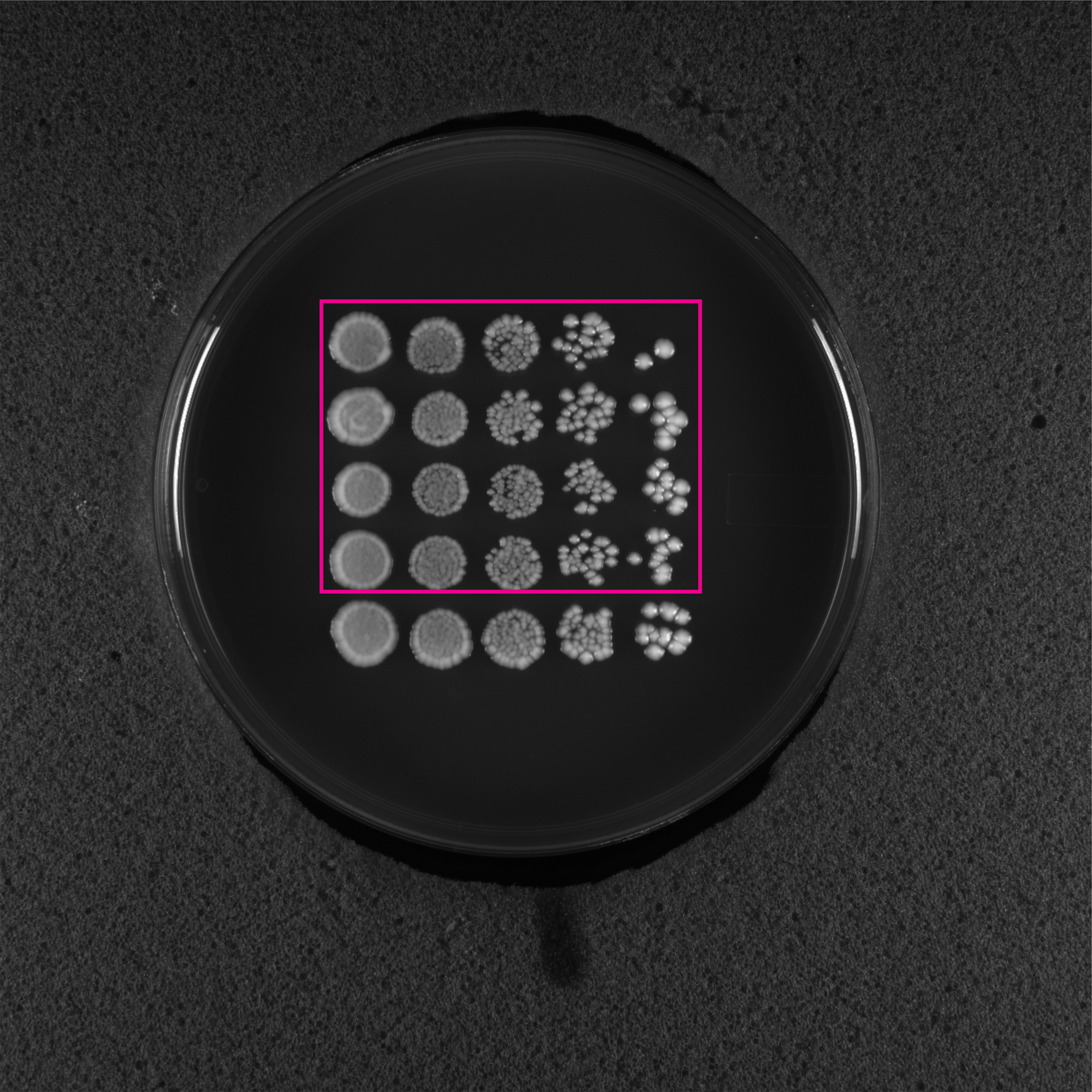

Supplement: Supplementary file 7 — Source data Fig. 5 [file 44318_2024_132_MOESM7_ESM.zip › Fig 5/5D/YPD, 3days, 30C.tif]

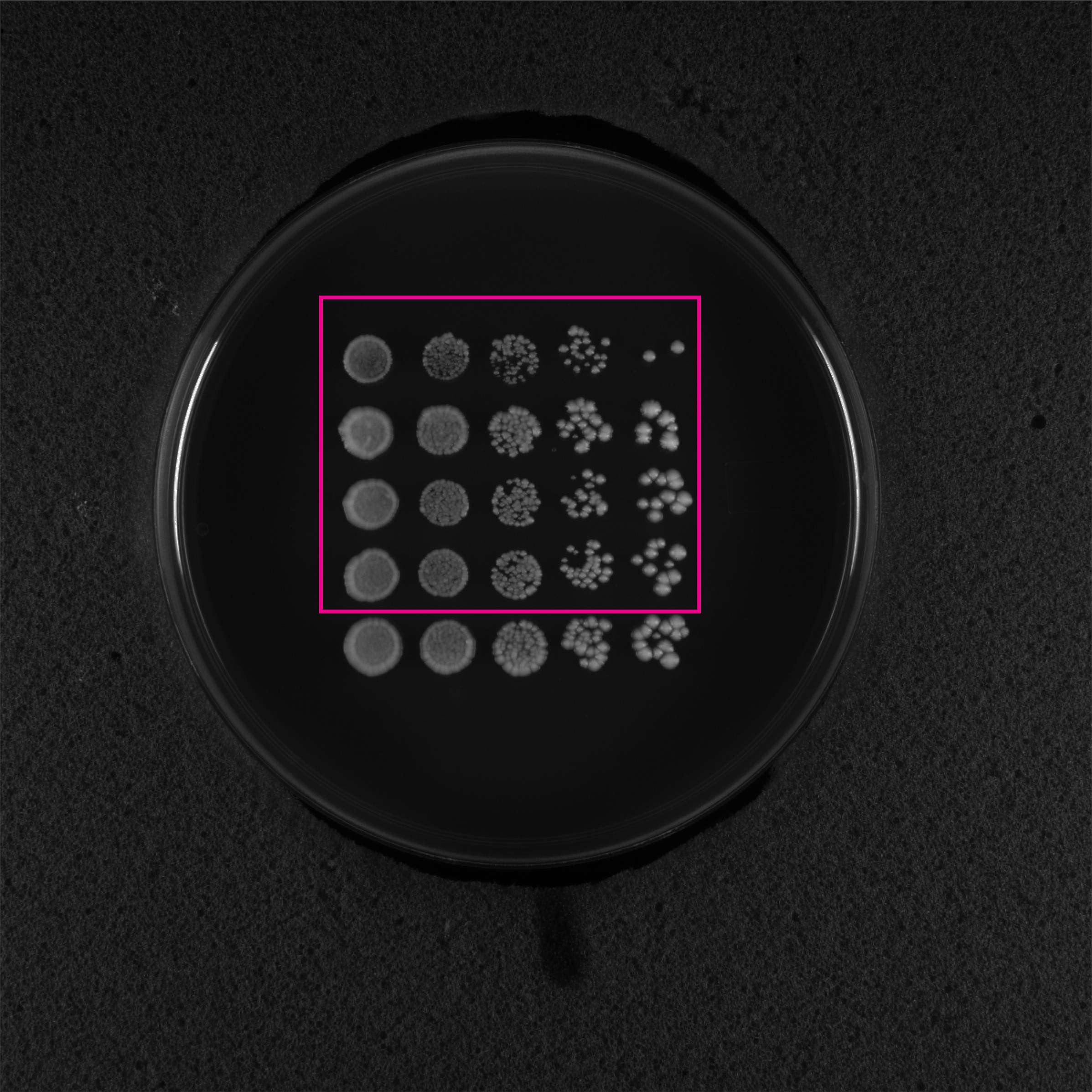

Supplement: Supplementary file 7 — Source data Fig. 5 [file 44318_2024_132_MOESM7_ESM.zip › Fig 5/5D/YPD, 3days, 37C.tif]

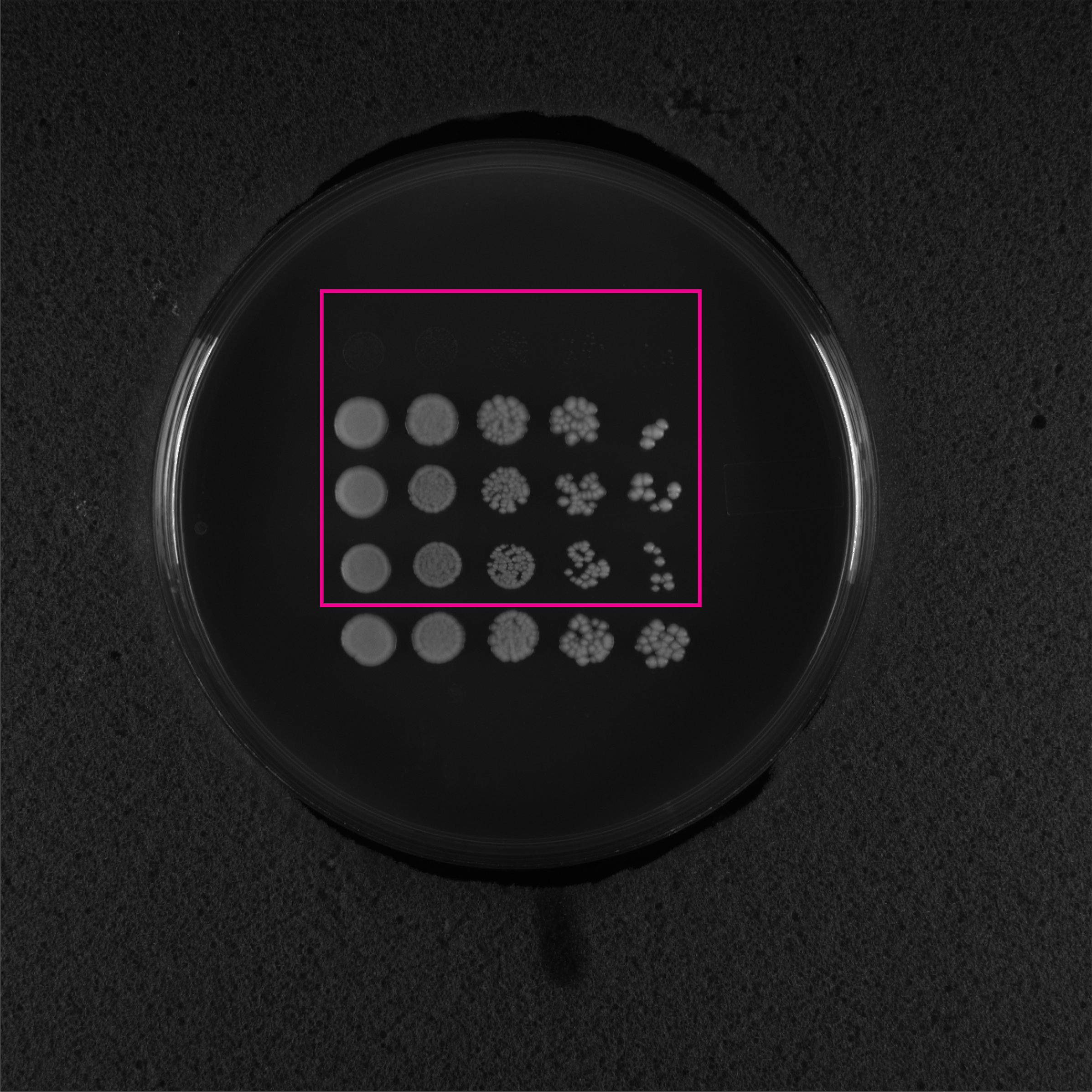

Supplement: Supplementary file 7 — Source data Fig. 5 [file 44318_2024_132_MOESM7_ESM.zip › Fig 5/5D/YPEG, 5days, 30C.tif]

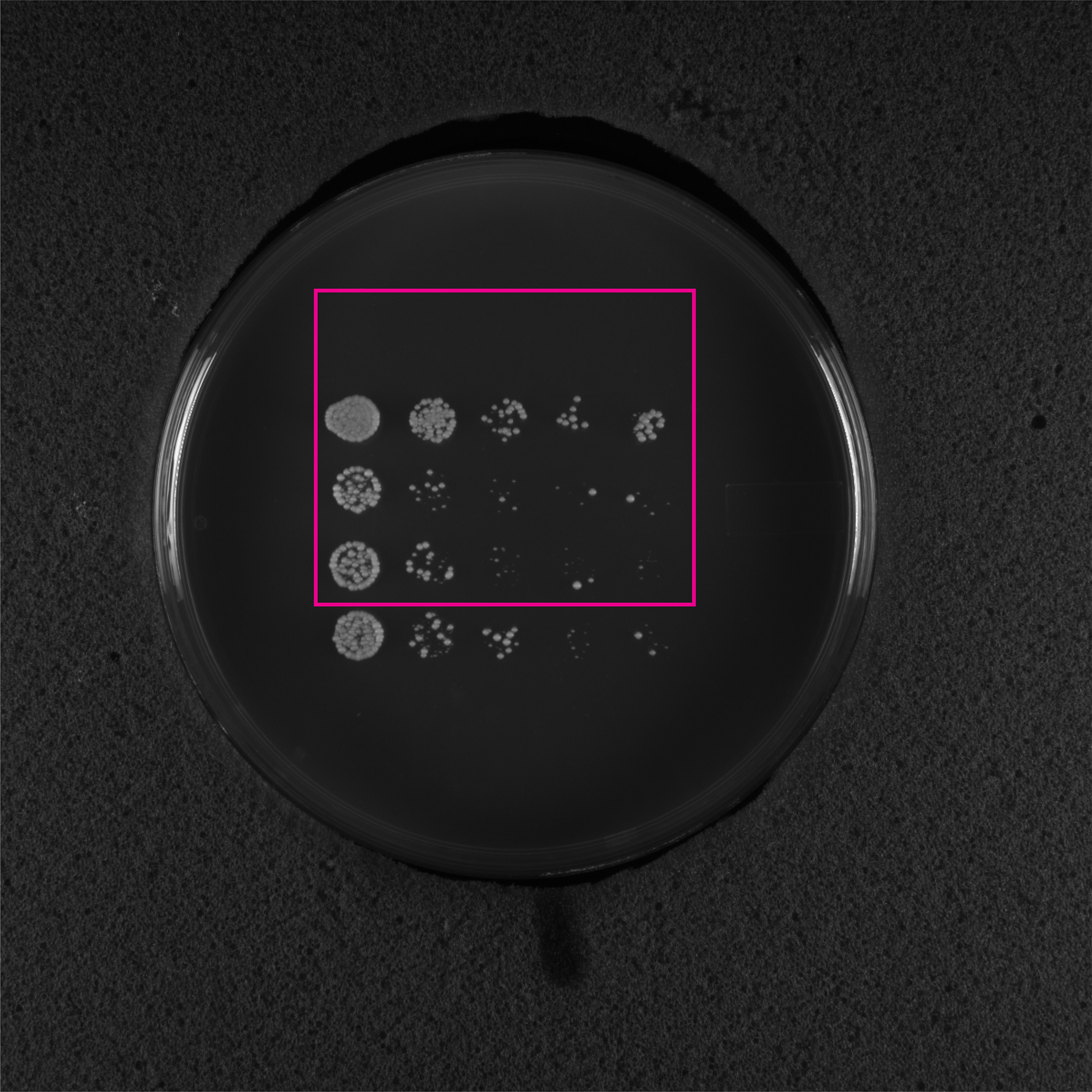

Supplement: Supplementary file 7 — Source data Fig. 5 [file 44318_2024_132_MOESM7_ESM.zip › Fig 5/5D/YPEG, 5days, 37C.tif]

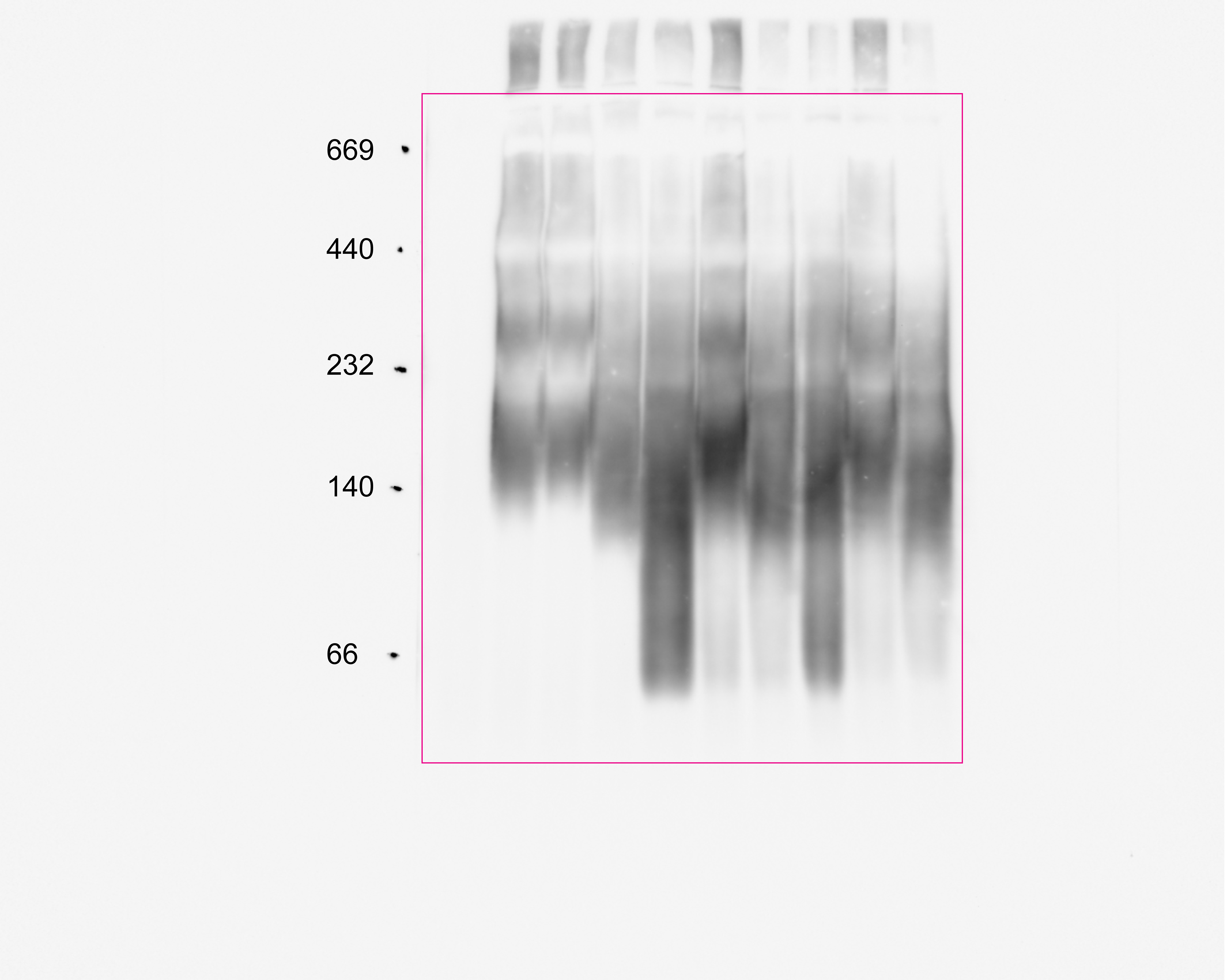

Supplement: Supplementary file 7 — Source data Fig. 5 [file 44318_2024_132_MOESM7_ESM.zip › Fig 5/5E/Blue native.tif]

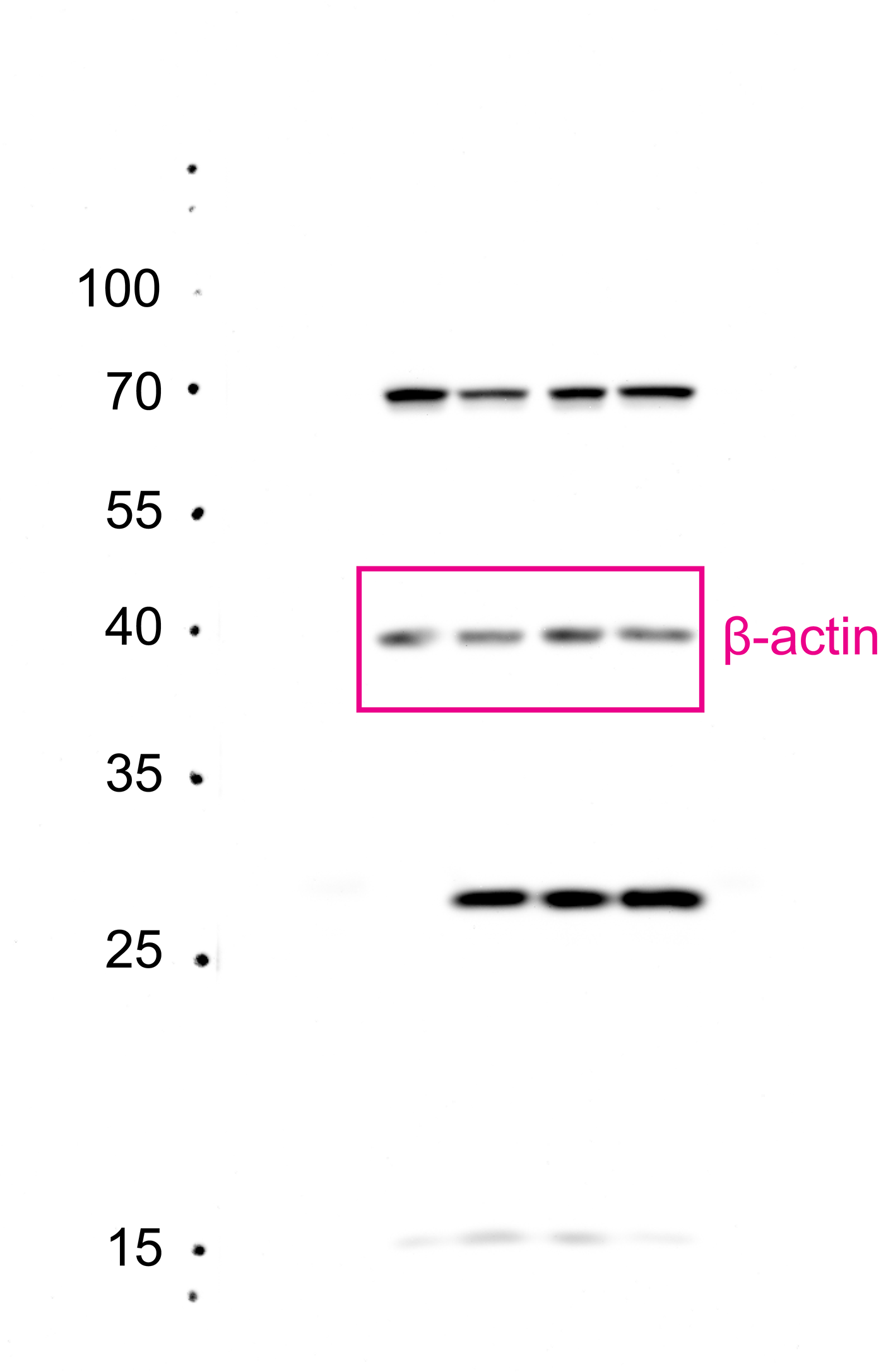

Supplement: Supplementary file 8 — Source data Fig. 6 [file 44318_2024_132_MOESM8_ESM.zip › Fig 6/6B/beta-actin.tif]

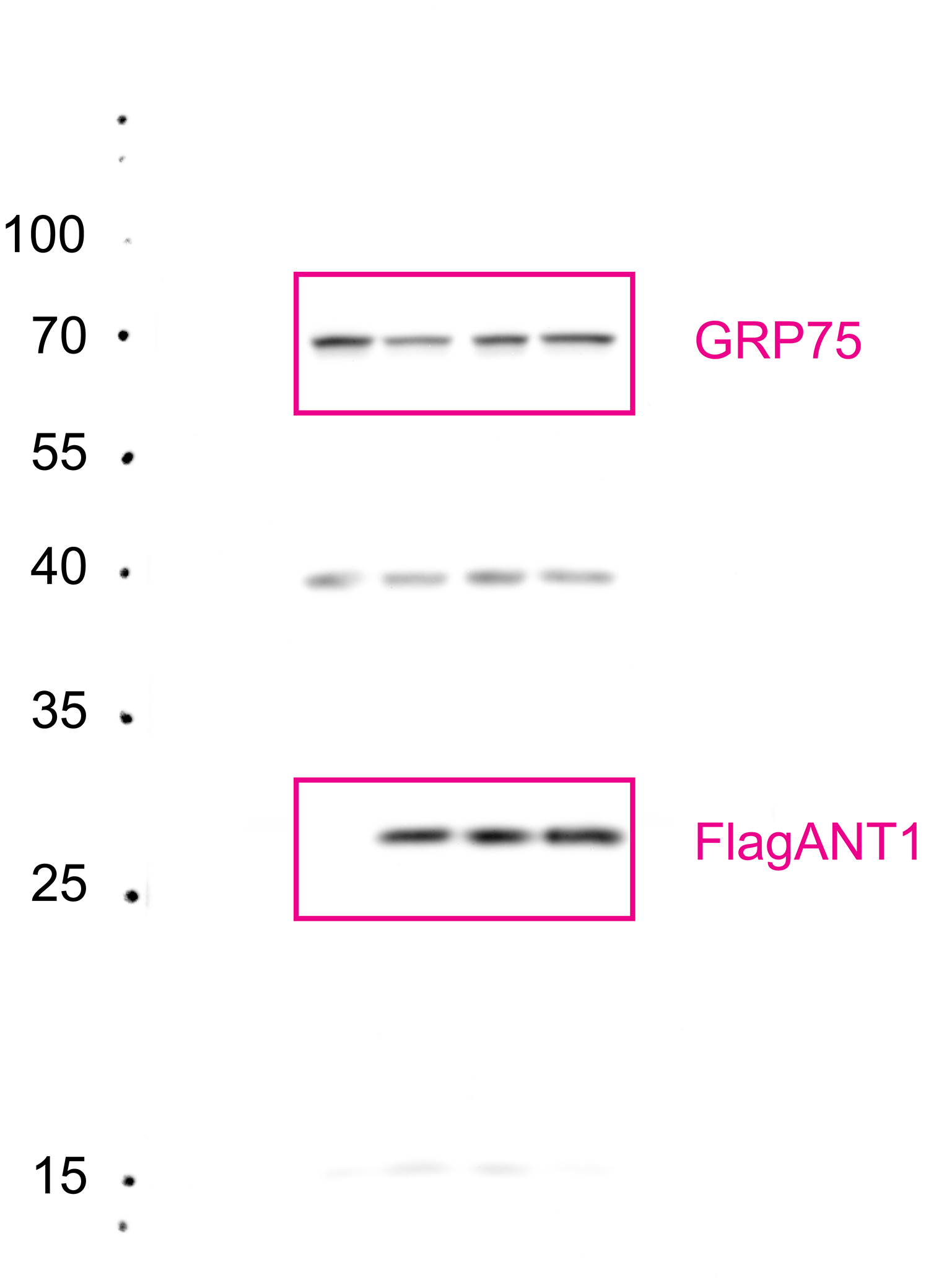

Supplement: Supplementary file 8 — Source data Fig. 6 [file 44318_2024_132_MOESM8_ESM.zip › Fig 6/6B/GRP75, ANT1.tif]

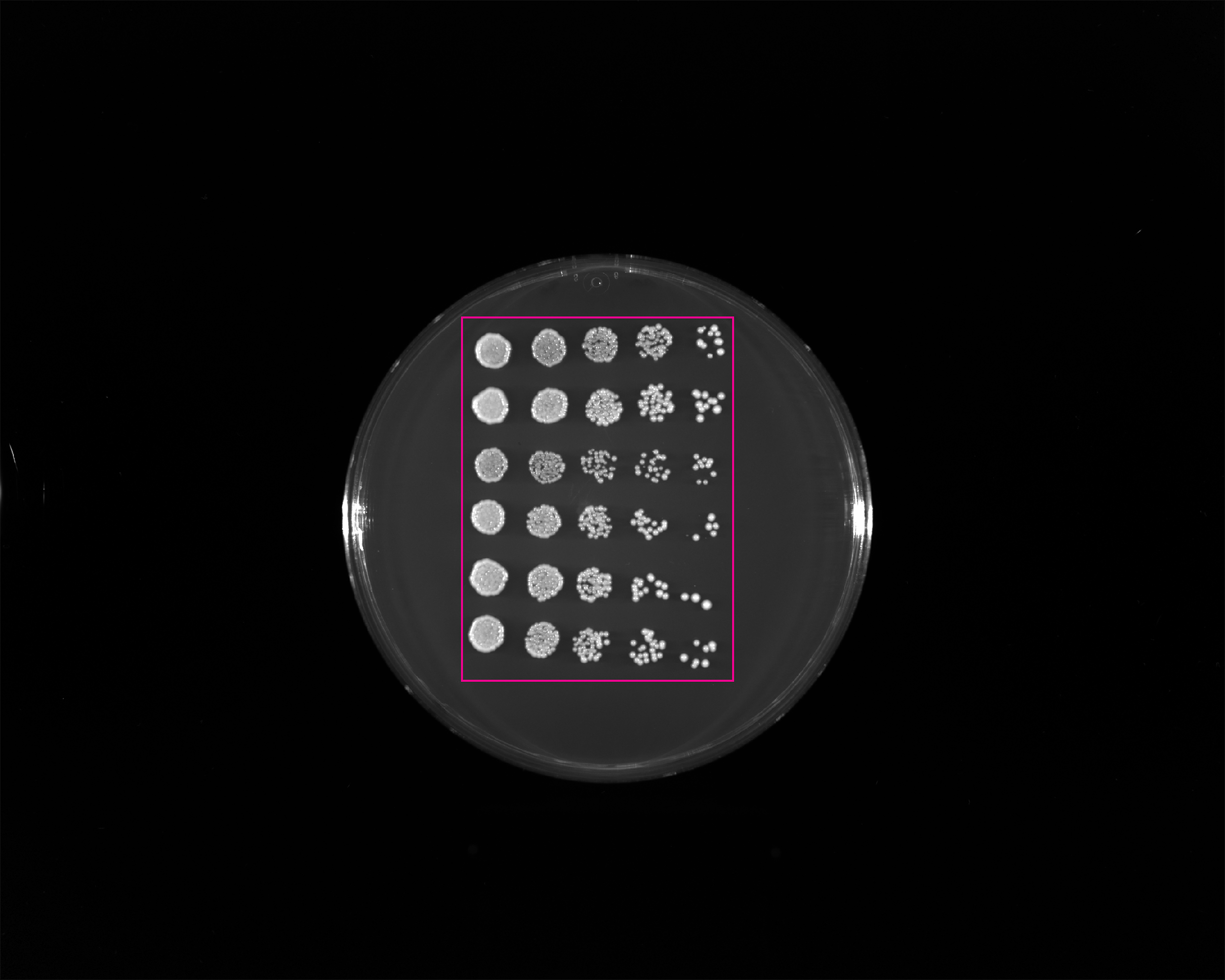

Supplement: Supplementary file 10 — Source data Fig. 8 [file 44318_2024_132_MOESM10_ESM.zip › Fig 8/8C/YPD, 2days, 30C.tif]

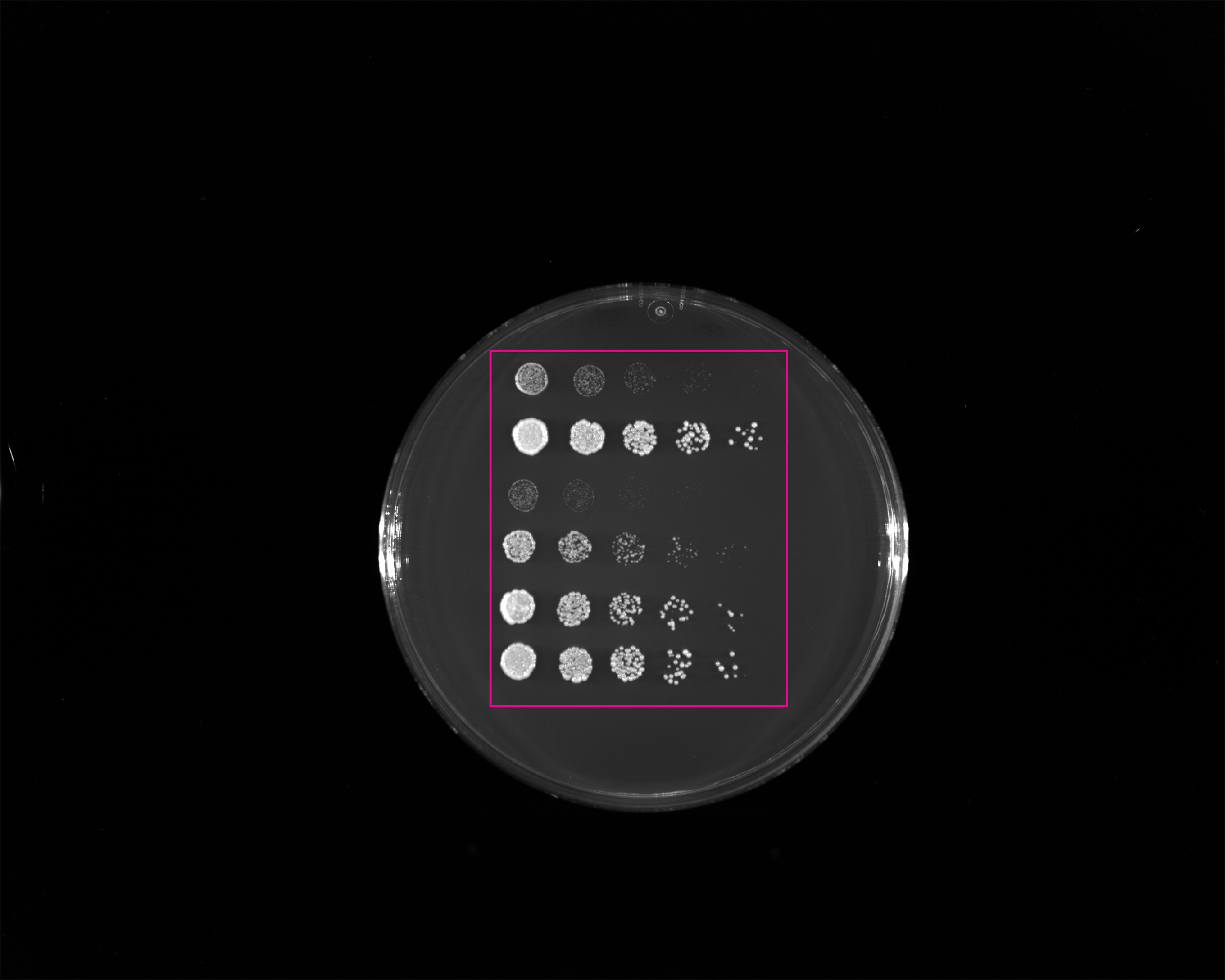

Supplement: Supplementary file 10 — Source data Fig. 8 [file 44318_2024_132_MOESM10_ESM.zip › Fig 8/8C/YPD, 3days, 37C.tif]

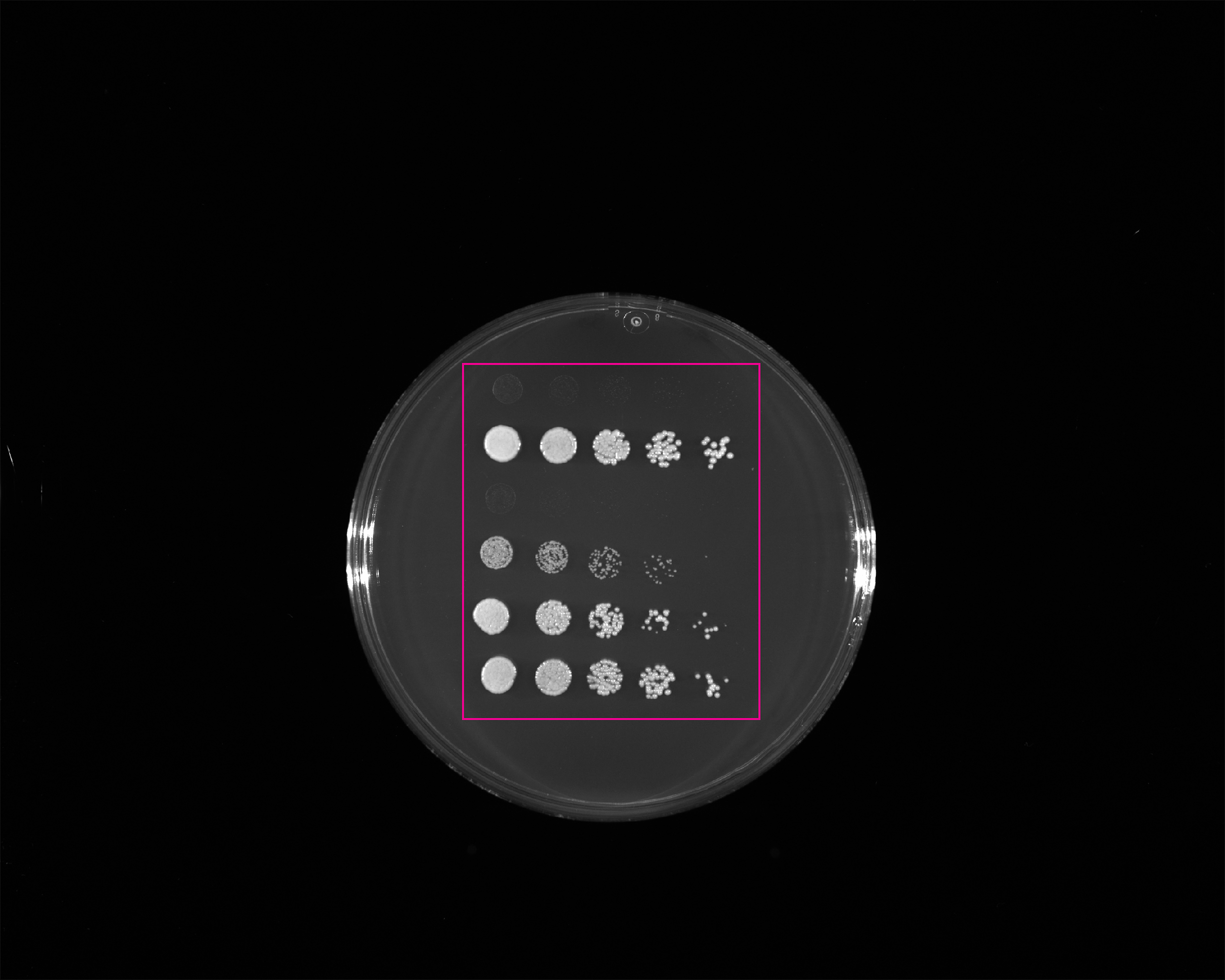

Supplement: Supplementary file 10 — Source data Fig. 8 [file 44318_2024_132_MOESM10_ESM.zip › Fig 8/8C/YPEG, 3days, 30C.tif]

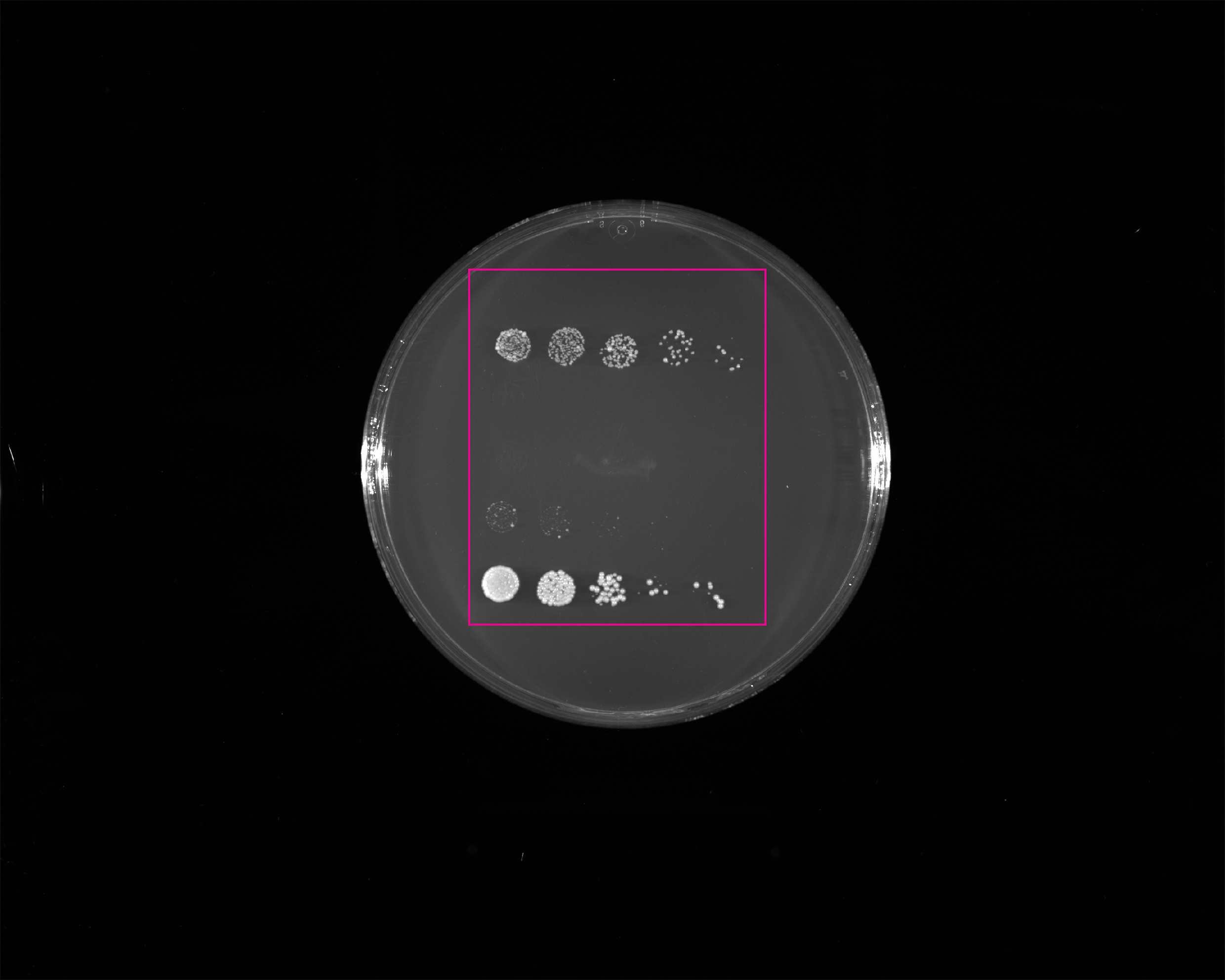

Supplement: Supplementary file 10 — Source data Fig. 8 [file 44318_2024_132_MOESM10_ESM.zip › Fig 8/8C/YPEG, 5days, 37C.tif]

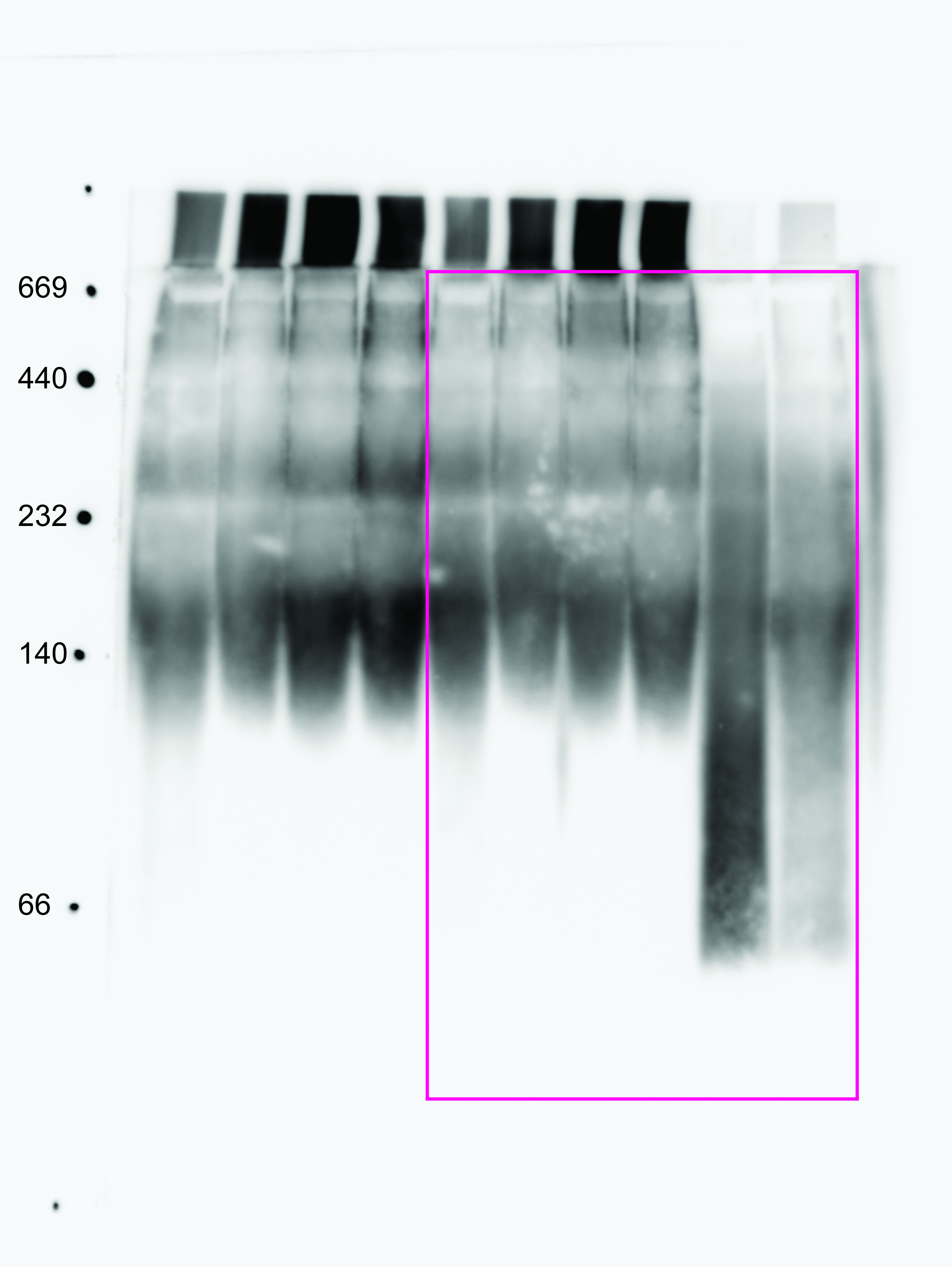

Supplement: Supplementary file 10 — Source data Fig. 8 [file 44318_2024_132_MOESM10_ESM.zip › Fig 8/8H/blue native.tif]
