# Supplementary material for: Functional diversity among cardiolipin binding sites on the mitochondrial ADP/ATP carrier
Source: EMBO J. 2024 Jun 5;43(14):2979–3008. doi: 10.1038/s44318-024-00132-2 (PMC11251061; doi:10.1038/s44318-024-00132-2)
Supplement: Supplementary file 11 — Expanded View Figures [file 44318_2024_132_MOESM11_ESM.pdf]

Expanded View Figures

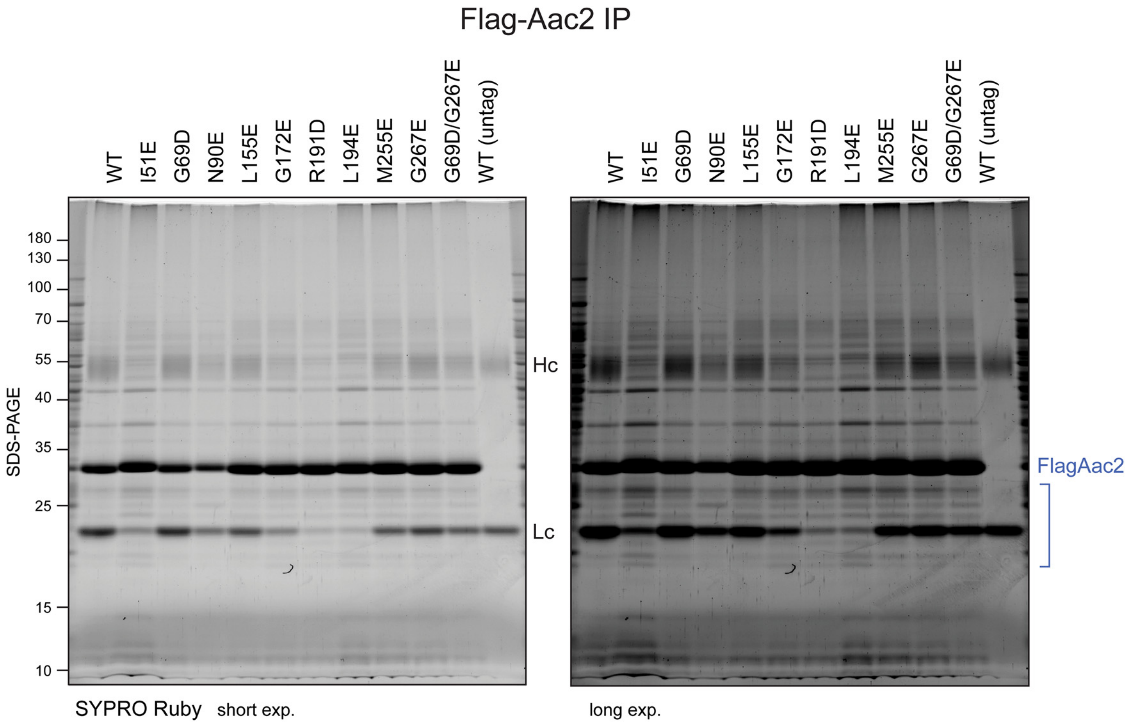

Figure EV1. Aac2 CL-binding mutants do not engage in aberrant protein interactions.

Isolated mitochondria were solubilized with 1.5% digitonin and subjected to FLAG immunoprecipitation. Co-purified extracts were resolved by 10–16% SDS-PAGE and resolved proteins detected by SYPRO Ruby staining.

 © The Author(s)

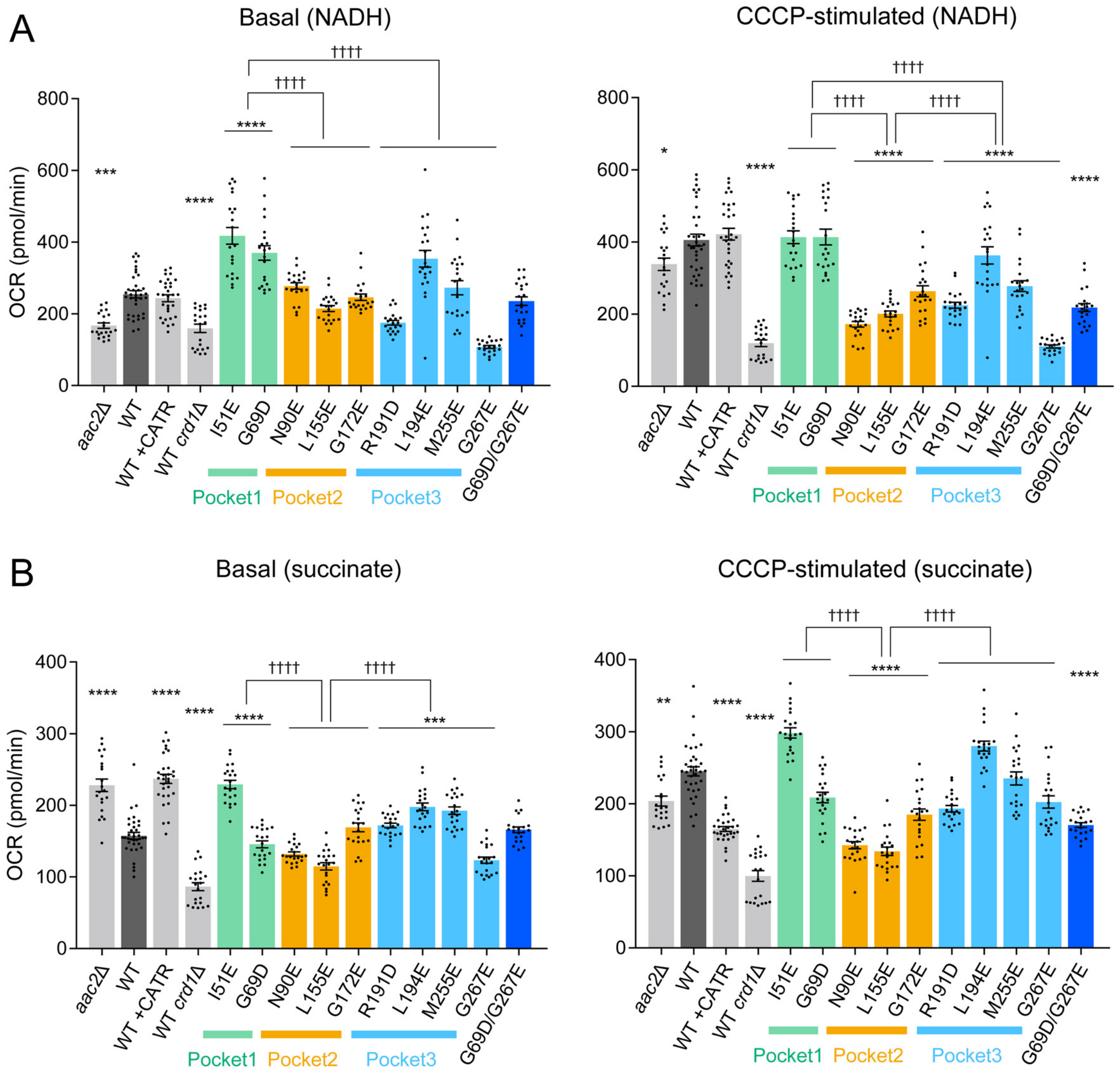

Figure EV2. Mitochondrial respiration of Aac2 CL-binding mutants.

Related to Fig. 4D–F, basal and CCCP-stimulated respirations of WT and mutant mitochondria in the presence of NADH (**A**) and succinate (**B**) were plotted as oxygen consumption rate (OCR) ($n = 21$–35, 3–5 biological replicates with 5–7 technical replicates). Mean with SEM. Significant differences obtained by two-way ANOVA followed by Tukey's multiple comparisons test are shown as * for comparison with WT and † for comparison between pockets; *$p < 0.05$, **$p < 0.01$, ***$p < 0.001$, ****$p < 0.0001$.

© The Author(s)

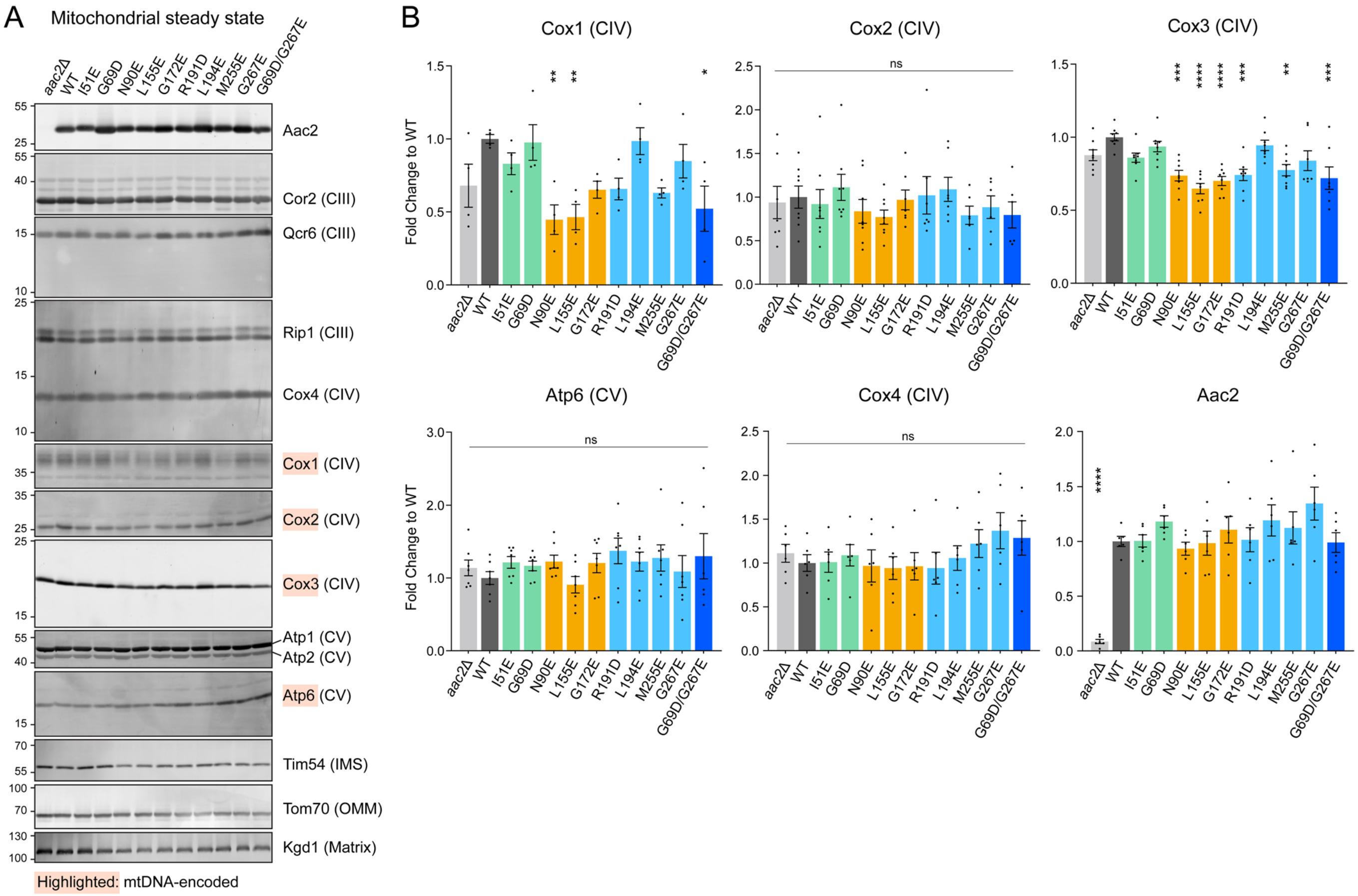

Figure EV3. The expression of respiratory complex subunits encoded in mitochondrial DNA is attenuated in Aac2 CL-binding mutants.

(**A**) Mitochondrial extracts were resolved by SDS-PAGE and immunoblotted for indicated proteins, including subunits of respiratory complexes III, IV, and V. (**B**) The expression of indicated respiratory complex subunits was quantified. Mean with SEM. Statistical differences were analyzed by one-way ANOVA followed by Dunnett's multiple comparison test; *$p < 0.05$, **$p < 0.01$, ***$p < 0.001$, ****$p < 0.0001$ (vs. WT). Representative images from the replicates ($n = 4$–8, biological replicates) are shown.

 © The Author(s)

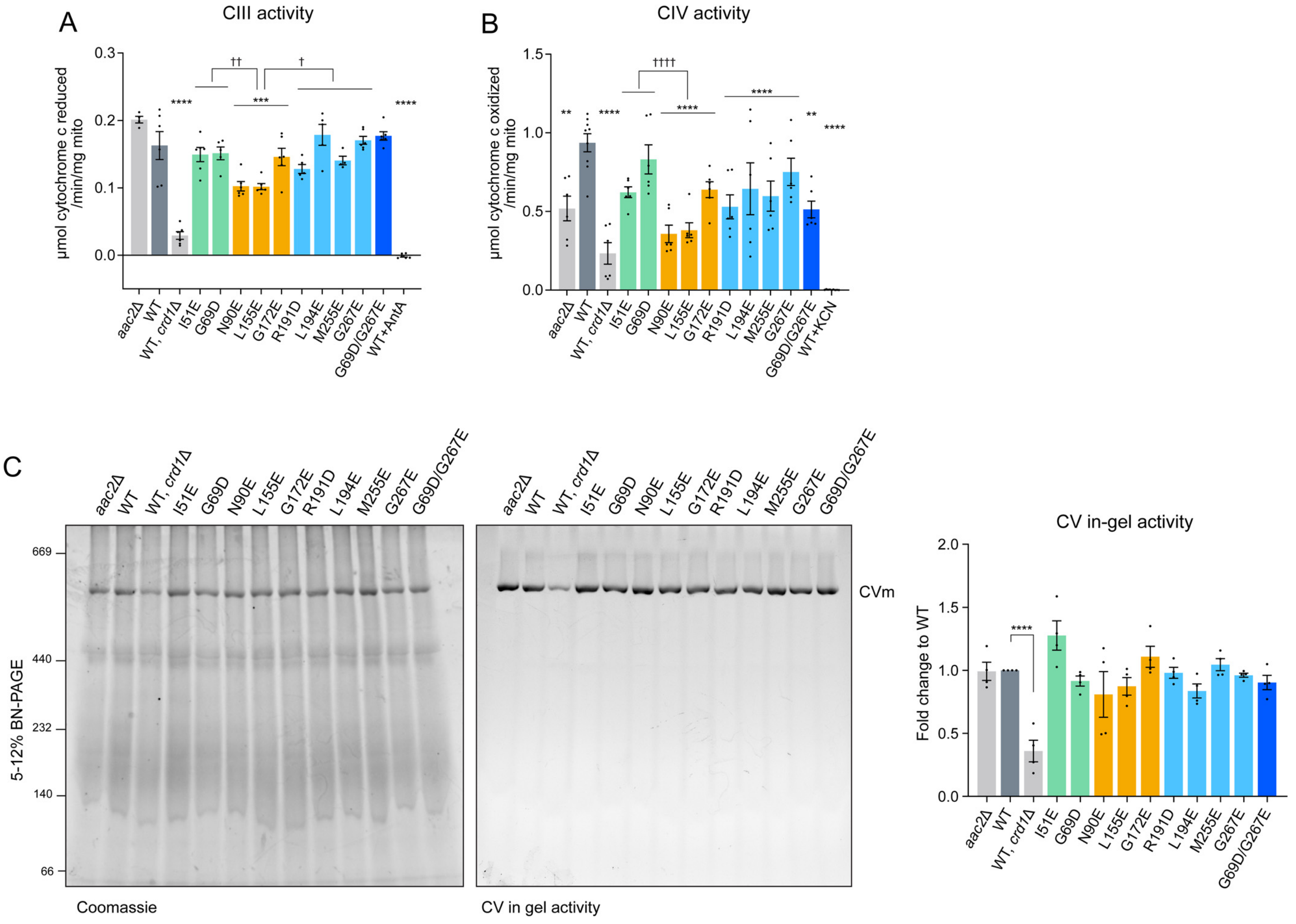

Figure EV4. Activities of respiratory complexes III, IV, and V of CL-binding mutants.

(**A**) Complex III activity in 0.5% (w/v) DDM-solubilized mitochondria ($n = 4$–6, biological replicates). (**B**) Complex IV activity in 0.5% (w/v) DDM-solubilized mitochondria ($n = 6$–9, biological replicates). (**C**) Complex V in-gel activity assay. Mitochondria were solubilized in 1% (w/v) DDM, resolved by 5–12% blue native-PAGE, and incubated with the substrate ($n = 5$, biological replicates). Mean with SEM. Significant differences obtained by two-way ANOVA followed by Tukey's multiple comparisons test are shown as * for comparison with WT and † for comparison between pockets; *$p < 0.05$, **$p < 0.01$, ***$p < 0.001$, ****$p < 0.0001$.

© The Author(s)

```
                                                                              I51                  G69
yAac2 MSSNAQVKTPLPPAPAPKKESNFLIDFLMGGVSAAVAKTAASPIERVKLLIQNQDEMLKQGTLDRKYAGILDCFKRTATQE
bANT1 ---------------MSDQALSFLKDFLAGGVAAAISKTAVAPIERVKLLLQVQHA-SKQISAEKQYKGIIDCVVRIPKEQ
hANT1 ---------------MGDHAWSFLKDFLAGGVAAAVSKTAVAPIERVKLLLQVQHA-SKQISAEKQYKGIIDCVVRIPKEQ
hANT2 ---------------MTDAAVSFAKDFLAGGVAAAISKTAVAPIERVKLLLQVQHA-SKQITADKQYKGIIDCVVRIPKEQ
hANT3 ---------------MTEQAISFAKDFLAGGIAAAISKTAVAPIERVKLLLQVQHA-SKQIAADKQYKGIVDCIVRIPKEQ
hANT4 MHREP---AKKKAEKRLFDASSFGKDLLAGGVAAAVSKTAVAPIERVKLLLQVQAS-SKQISPEARYKGMVDCLVRIPREQ

              N90                                                                      L155
yAac2 GVISFWRGNTANVIRYFPTQALNFAFKDKIKAMF--GFKKEEGYAKWFAGNLASGGAAGALSLLFVYSLDYARTRLAADSK
bANT1 GFLSFWRGNLANVIRYFPTQALNFAFKDKYKQIFLGGVDRHKQFWRYFAGNLASGGAAGATSLCFVYPLDFARTRLAADVG
hANT1 GFLSFWRGNLANVIRYFPTQALNFAFKDKYKQLFLGGVDRHKQFWRYFAGNLASGGAAGATSLCFVYPLDFARTRLAADVG
hANT2 GVLSFWRGNLANVIRYFPTQALNFAFKDKYKQIFLGGVDKRTQFWLYFAGNLASGGAAGATSLCFVYPLDFARTRLAADVG
hANT3 GVLSFWRGNLANVIRYFPTQALNFAFKDKYKQIFLGGVDKHTQFWRYFAGNLASGGAAGATSLCFVYPLDFARTRLAADVG
hANT4 GFFSFWRGNLANVIRYFPTQALNFAFKDKYKQLFMSGVNKEKQFWRWFLANLASGGAAGATSLCVVYPLDFARTRLGVDIG
                                                                                ↑
                                                                          hANT1 L141
                 G172              R191 L194
yAac2 SSKKGGARQFNGLIDVYKKTLKSDGVAGLYRGFLPSVVGIVVYRGLYFGMYDSLKPLLLTGSLEGSFLASFLLGWVVTTGA
bANT1 --KGAAQREFTGLGNCITKIFKSDGLRGLYQGFNVSVQGIIIYRAAYFGVYDTAKGMLPD-PKNVHIIVSWMIAQTVTAVA
hANT1 --KGAAQREFHGLGDCIIKIFKSDGLRGLYQGFNVSVQGIIIYRAAYFGVYDTAKGMLPD-PKNVHIFVSWMIAQSVTAVA
hANT2 --KAGAEREFRGLGDCLVKIYKSDGIKGLYQGFNVSVQGIIIYRAAYFGIYDTAKGMLPD-PKNTHIVISWMIAQTVTAVA
hANT3 --KSGTEREFRGLGDCLVKITKSDGIRGLYQGFSVSVQGIIIYRAAYFGVYDTAKGMLPD-PKNTHIVVSWMIAQTVTAVA
hANT4 --KGPEERQFKGLGDCIMKIAKSDGIAGLYQGFGVSVQGIIVYRASYFGAYDTVKGLLPK-PKKTPFLVSFFIAQVVTTCS

                 M255        G267
yAac2 STCSYPLDTVRRRMMMTSGQ---AVKYDGAFDCLRKIVAAEGVGSLFKGCGANILRGVAGAGVISMYDQLQMILFGKKFK
bANT1 GLVSYPFDTVRRRMMMQSGRKGADIMYTGTVDCWRKIAKDEGPKAFFKGAWSNVLRGMGGAFVLVLYDEIKKFV------
hANT1 GLVSYPFDTVRRRMMMQSGRKGADIMYTGTVDCWRKIAKDEGAKAFFKGAWSNVLRGMGGAFVLVLYDEIKKYV------
hANT2 GLTSYPFDTVRRRMMMQSGRKGTDIMYTGTLDCWRKIARDEGGKAFFKGAWSNVLRGMGGAFVLVLYDEIKKYT------
hANT3 GVVSYPFDTVRRRMMMQSGRKGADIMYTGTVDCWRKIFRDEGGKAFFKGAWSNVLRGMGGAFVLVLYDELKKVI------
hANT4 GILSYPFDTVRRRMMMQVINFLI--NYNSKLHL-------------KNLEW-----------------------------
```

yAac2 Pocket1

yAac2 Pocket2

yAac2 Pocket3

Figure EV5. CL-binding sites are conserved across species.

Amino acid sequence alignment of yeast Aac2, bovine ANT1, and human ANT isoforms. The residues designed for the Aac2 CL-binding mutants are highlighted as indicated.

 © The Author(s)
